# Novel Copper Chelators Enhance Spatial Memory and Biochemical Outcomes in Alzheimer’s Disease Model

**DOI:** 10.1021/acschemneuro.5c00291

**Published:** 2025-08-15

**Authors:** Mariana L. M. Camargo, Augusto B. Farias, Giovana B. Bertazzo, Rafael N. Gomes, Kaio S. Gomes, Lucas M. Bosquetti, Silvia H. Takada, Felipe C. Braga, Caroline C. Augusto, Bruno L. Batista, Kleber T. de Oliveira, Giselle Cerchiaro

**Affiliations:** † Metal Biochemistry and Oxidative Stress Laboratory, Center for Natural Sciences and Humanities, 488580 Federal University of ABC-UFABC, Santo André, São Paulo 09210-580, Brazil; ‡ Laboratory of Neurogenetics, Center for Mathematics, Computing and Cognition, 74362 Federal University of ABC-UFABC, São Bernardo do Campo, São Paulo 09606-045, Brazil; § Departament of Chemistry, 67828 Federal University of São Carlos-UFSCar, São Carlos, São Paulo 13565-905, Brazil

**Keywords:** Alzheimer’s disease, preclinical trials, copper, copper chelators, imine, quinoline, medicinal chemistry, neuroscience, bioinorganic chemistry, drug development, oxidative stress, neurodegeneration

## Abstract

This study explores the potential of novel molecules that can act as copper chelators to treat Alzheimer’s disease. Eight imines **L03–10** and one quinoline-based compound **L11** were synthesized, characterized, and evaluated as compounds that can act to reverse neurodegeneration in vivo. Their ability to extract copper from the Cu-β-amyloid complex, a key factor in Alzheimer’s pathology, was assessed, achieving a remarkable in vitro activity for **L09**, **L10**, and **L11**. They effectively extracted it from the Cu-β-amyloid complex, which was confirmed using electron paramagnetic resonance (EPR) spectroscopy. In silico studies predicted that compounds **L09**, **L10**, and **L11** demonstrated favorable absorption, distribution, metabolism, and excretion (ADME) properties, suggesting suitability for oral administration and blood-brain barrier permeability. Cellular studies showed that compounds **L09** and **L10** (at concentrations up to 500 μM) exhibited low cytotoxicity. They reduced lipid peroxidation and DNA damage induced by beta-amyloid oligomers at lower concentrations. Compound **L11** showed more significant cytotoxicity but reduced beta-amyloid-induced DNA damage. In vivo studies (STZ-induced Alzheimer’s rat model) proved that compound **L10** significantly reduced neuroinflammation, oxidative stress, and restored copper homeostasis in the hippocampus. This was accompanied by improved spatial memory performance in the Barnes maze test. Compounds **L09** and **L11** showed less impact on these parameters. The study presents compelling evidence that specifically designed copper chelators could offer a new therapeutic strategy for Alzheimer’s disease. Compound **L10** is an up-and-coming candidate and warrants further investigation. The detailed in silico, in vitro, and in vivo analyses provide a solid motivation for future research and drug development efforts.

## Graphical abstract

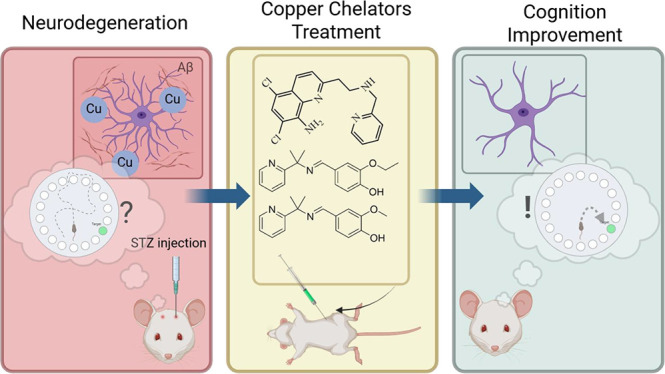

## Introduction

Alzheimer’s disease (AD) is a progressive and irreversible neurodegeneration characterized by symptoms such as cognitive impairment, memory loss, and language difficulties. Despite its high prevalence, the etiology of the disease remains unknown, with no cure and limited therapeutic options that provide only symptomatic relief^1^ (e.g., three acetylcholinesterase inhibitors and memantine, an *N*-methyl-d-aspartate receptor antagonist).^2,3^ After nearly 20 years without prospects for new Alzheimer’s drugs, the FDA granted accelerated approval to monoclonal antibodies targeting protofibrils in brains with dementia. However, the use of these drugs is associated with a range of severe side effects.^4–6^ Therefore, exploring new approaches and focusing on alternative therapeutic targets is essential.^7^

In this context, studies have emerged focusing on developing selective copper chelators as a new therapeutic strategy.^8–11^ To be effective, chelators need specific characteristics, such as low molar mass (preferably less than 500 Da) and high lipophilicity, allowing them to cross the blood-brain barrier.^9^ The first in vivo study on using a selective copper chelator derived from 8-hydroxyquinoline, clioquinol (Figure 1a), demonstrated a significant reduction in amyloid plaques and insoluble beta-amyloid in the brains of Alzheimer’s mice.^12–14^ However, despite promising results in phase II clinical trials, the development of clioquinol was halted due to manufacturing issues, contamination with carcinogenic compounds, and neurotoxicity linked to zinc chelation.^15,16^ The compound PBT2 (Figure 1b) was developed to overcome these limitations, offering better brain penetration, higher solubility, and efficacy. Like clioquinol, PBT2 inhibits the interaction between beta-amyloid and metals but with controlled affinity to avoid interfering with the functions of cuproproteins. Despite advances, the development of PBT2 was also discontinued due to difficulties in large-scale production and the absence of significant effects on beta-amyloid plaque clearance^8,17,18^

**Figure 1:**
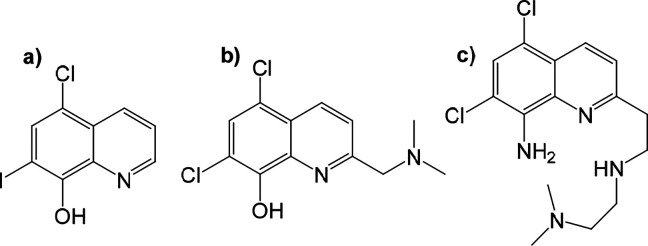
Structures of (a) clioquinol (b) PBT2 and (c) TDMQ20.

To obtain molecules with specific characteristics as promising candidates for Alzheimer’s therapy testing, Meunier and colleagues developed a series of tetradentate chelators with a mono­(8-amino)­quinoline-based structure called TDMQ (Figure 1c). In vitro tests showed that these chelators efficiently extract Cu­(II) from the Cu-Aβ complex, acting catalytically. In vivo tests demonstrated that the compounds can inhibit memory loss in nontransgenic amyloid-deficient mice.^19^

The quinoline compound synthesized in this work was inspired by the tetradentate chelators TDMQ, which, as previously reported, are chelators with high affinity for Cu­(II), form a stable complex with the metal ion, and are capable of inhibiting memory loss in a nontransgenic Alzheimer’s animal model, all without modifying metalloenzymes or metal-containing cofactors^19,20^

The iminic compounds obtained in this work were considered for several reasons, such as the fact that they are nontoxic, are good chelators of metal ions, including Cu­(II), are easy to obtain from relatively uncomplicated syntheses,^21–23^ and are still little explored as Cu­(II) chelators for the treatment of Alzheimer’s disease, with only the work of Gharai et al.^24^ having this purpose, but still without in vivo tests.

Although not the only target, the regulation of copper homeostasis is becoming a central focus for Alzheimer’s treatment.^19,25–27^ Herein, we report the synthesis and characterization of nine novel copper chelators, eight imine and one quinoline-based, and the results of in silico, in vitro, and in vivo studies in a streptozotocin (STZ) model of Alzheimer’s are also presented.

## Results

Aiming to obtain new copper­(II) chelators able to be used in vivo to reverse AD symptoms and neurodegeneration in an animal model, eight novel imine compounds (**L03–L10**) were synthesized through the classical condensation reaction between amines and aldehydes in ethanol,^23,28^ overnight (Figure 2a). Detailed methodology for the synthesis of each compound is available in the Supporting Information. Also, a quinoline-based compound, **L11**, was synthesized following a five-step synthetic route (Figure 2b). The first three steps of the synthetic route were performed according to protocols described in the literature.^29^ ^1^H and ^13^C NMR spectroscopy and high-resolution mass spectrometry confirmed the chemical structure of all new compounds (Supporting Information).

**Figure 2:**
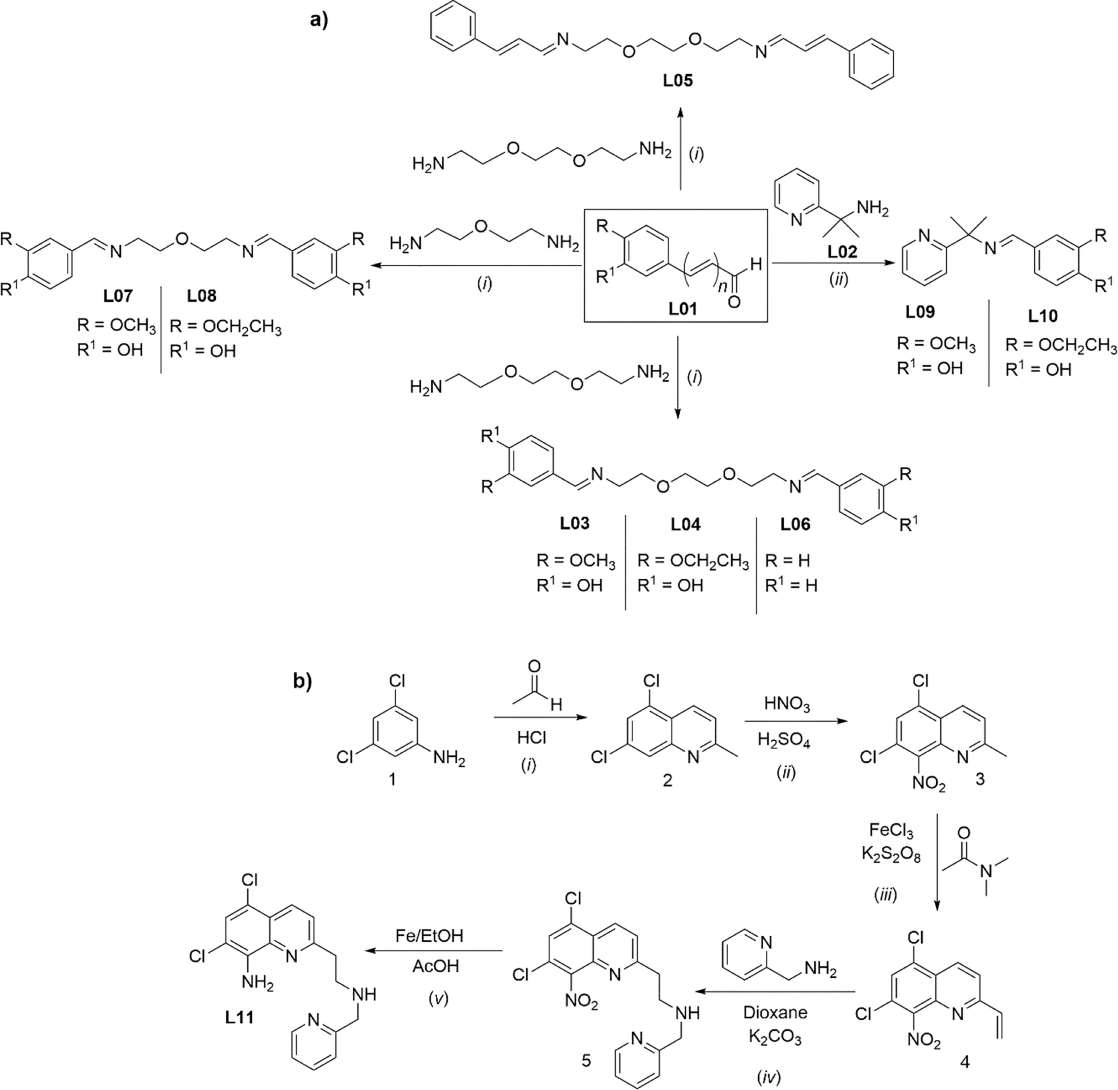
Synthesis scheme for the ligands (a) **L03–L10**. Reagents and reaction conditions: (i) amine (1.0 equiv), aldehyde (2.0 equiv), EtOH, reflux, overnight. (ii) Amine (1.05 equiv), aldehyde (1.0 equiv), toluene, reflux, and (b) **L11**. Reagents and reaction conditions: (i) acetaldehyde (4.1 equiv), HCl 12 M 0–80 °C, 4 h; (ii) HNO_3_ (4.8 equiv), H_2_SO_4_, r.t., 2 h; (iii) FeCl_3_ (3 mol %), K_2_S_2_O_8_ (2.0 equiv), dimethylacetamide (DMA), 110 °C, overnight; (iv) 2-picolylamine (2.0 equiv), K_2_CO_3_ (1.2 equiv), 1,4-dioxane, r.t., 1 h; (v) Fe° (3.0 equiv), AcOH (10 equiv), EtOH, reflux, 4 h.

Specific properties are essential for a compound to act as an effective drug. These properties must ensure that the compound reaches the target at an adequate concentration and remains sufficiently stable in the biological environment to perform in its active form. An ADME (absorption, distribution, metabolism, and excretion) study was conducted to evaluate these parameters. In this study, the evaluation of such parameters was performed in silico using the SwissADME platform.^30,31^ The results obtained from the bioavailability radar (Figure 3a) suggest good adherence of compounds **L07–L11** (especially **L09** and **L10**) to the evaluated parameters (lipophilicity, size, polarity, solubility, degree of unsaturation, and flexibility). The ADME study also contributed to selecting compounds that advanced to in vivo assays. As shown in Table 1, all new compounds exhibit a log *P*_o/w_ in the range between 0 and 5, indicating the ideal balance between lipophilicity, necessary to facilitate absorption and penetration into tissues, and hydrophilicity, necessary for good drug excretion. The compounds also demonstrated high gastrointestinal absorption in the in silico studies, suggesting oral administration as a promising route, along with the same bioavailability score (0.85) and no alerts for PAINS (pan-assay interference compounds). However, some parameters distinguish the compounds **L09**, **L10**, and **L11** from the others. These three compounds could cross the blood-brain barrier, as confirmed by their calculated topological polar surface area (TPSA) values, less than 90 Å^2^, a characteristic shared by only two other compounds.

**Figure 3:**
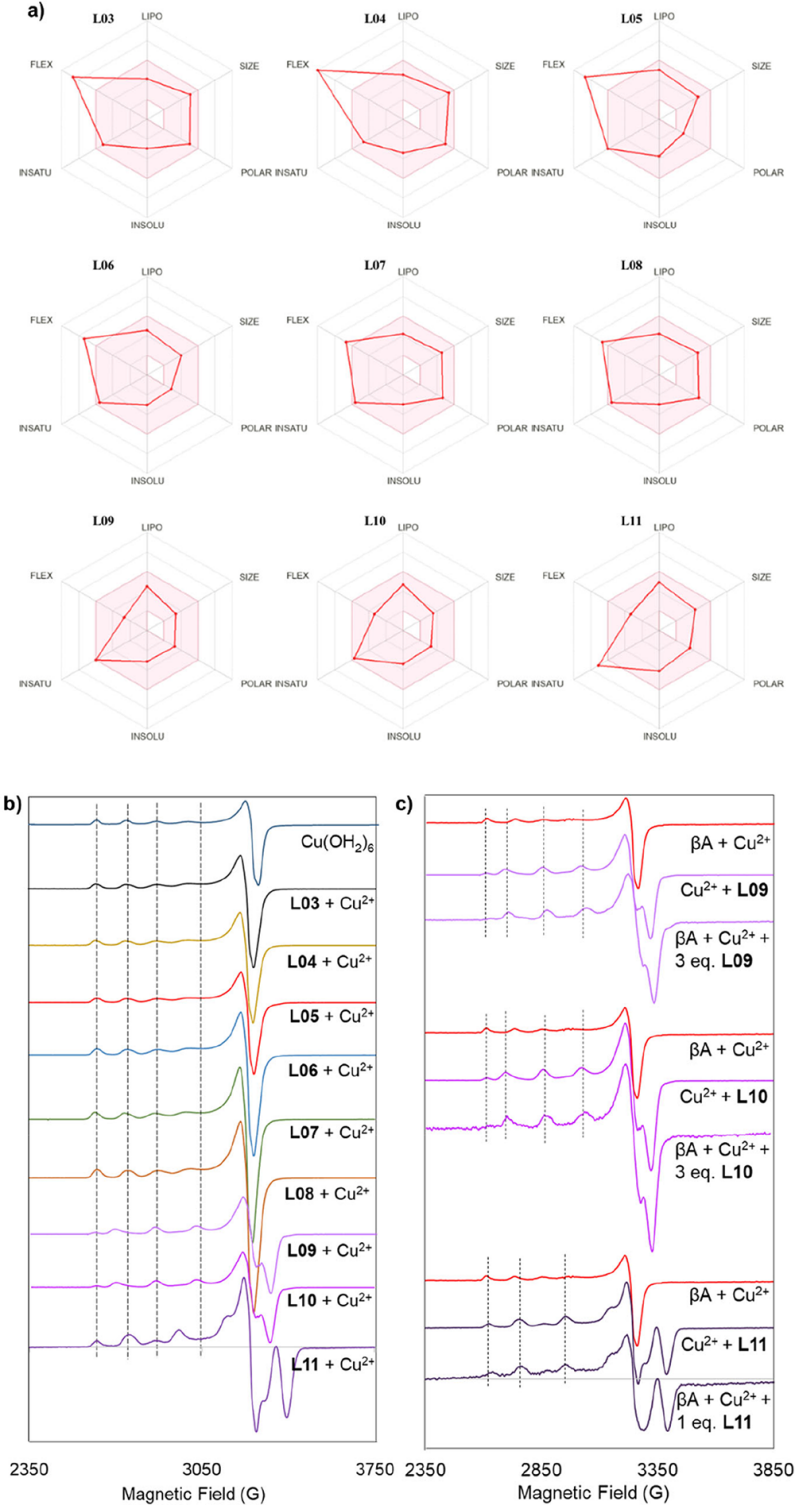
Panel showing (a) in silico bioavailability study using the SwissADME tool to demonstrate the potential of compounds **L03–L11** as successful drug candidates (the red area represents the optimal range for each property). ADME: absorption, distribution, metabolism, and excretion. (b) EPR experiments to confirm the formation of Cu­(II)­L complexes and comparison with the EPR spectrum of Cu­(H_2_O)_6_. [L] = [Cu­(II)] = 1.5 mM. 10% glycerol was used as a cryoprotectant. *T* = 77K. (c) Competition experiments for Cu­(II) between the Aβ_1–42_ peptide and ligands **L09**, **L10**, and **L11**. [L] = [Aβ_1–42_] = [Cu­(II)] = 200 μM. 10% glycerol was used as a cryoprotectant. *T* = 77 K.

**Table 1:** Physicochemical Properties and ADME Predictions for Compounds **L03–L11**

**physical**–**chemical properties**	L03	L04	L05	L06	L07	L08	L09	L10	L11
Parameter									
number of heavy atoms	30	32	28	24	27	29	20	21	23
number of aromatics heavy atoms	12	12	12	12	12	12	12	12	16
fraction Csp^3^	0.36	0.42	0.25	0.30	0.30	0.36	0.25	0.29	0.18
number of rotatable bonds	13	15	13	11	10	12	4	5	5
number of H-bond acceptors	8	8	4	4	7	7	4	4	3
number of H-bond donors	2	2	0	0	2	2	1	1	2
molar refractivity	116.11	125.73	118.95	99.08	105.41	115.03	80.19	85.00	95.83
TPSA (Å^2^)	102.10	102.10	43.18	43.18	92.87	92.87	54.71	54.71	63.83
log *P*_o/w_	2.79	3.45	4.39	3.47	2.70	3.38	2.52	2.94	3.33
Pharmacokinetics									
Gl absorption	high	high	high	high	high	high	high	high	high
BBB permeation	no	no	yes	yes	no	no	yes	yes	yes
P-gp substrate	no	no	no	no	no	no	no	no	yes
CYP1A2 inhibitor	no	no	yes	yes	no	no	yes	yes	yes
CYP2C19 inhibitor	no	no	yes	yes	no	no	yes	yes	yes
CYP2C9 inhibitor	no	no	no	no	no	no	no	no	no
CYP2D6 inhibitor	yes	yes	yes	yes	yes	yes	no	yes	yes
CYP3A4 inhibitor	no	no	yes	yes	yes	yes	no	no	yes
log *K*_p_ (skin permeation) (cm s^–1^)	–7.63	–7.28	–6.24	–6.53	–7.26	–6.90	–6.29	–6.12	–6.22
Bioavailability									
Lipinski	yes	yes	yes	yes	yes	yes	yes	yes	yes
Ghose	yes	yes	yes	yes	yes	yes	yes	yes	yes
Veber	no	no	no	no	no	no	yes	yes	yes
Eggan	yes	yes	yes	yes	yes	yes	yes	yes	yes
Muegge	yes	yes	yes	yes	yes	yes	yes	yes	yes
bioavailability score	0.55	0.55	0.55	0.55	0.55	0.55	0.55	0.55	0.55
Medicinal Chemistry									
PAINS	0 alert	0 alert	0 alert	0 alert	0 alert	0 alert	0 alert	0 alert	0 alert
Brenk’s	1 alert	1 alert	1 alert	1 alert	1 alert	1 alert	1 alert	1 alert	1 alert
synthetic aceessibility	3.62	3.88	3.94	3.31	3.26	3.52	2.72	2.85	2.36

The passage outlines the drug candidacy evaluation for a series of chemical compounds based on two critical criteria: Lipinski’s Rule of Five and the Veber filter. Lipinski’s Rule of Five is a set of guidelines predicting whether a compound has the right properties to be an orally active drug in humans. All evaluated compounds meet this rule, indicating potential suitability for oral administration. Veber filter is another criterion that focuses on oral bioavailability, considering factors like molecular flexibility and polar surface area. Only the compounds **L09**, **L10**, and **L11** fulfill this filter, suggesting these compounds are more likely to succeed as oral drugs. The compounds **L09**, **L10**, and **L11** do not inhibit the enzyme CYP2C9 but inhibit CYP1A2 and CYP2C19. These enzymes are involved in drug metabolism, affecting how drugs are processed in the body. The ability to inhibit these enzymes may influence the pharmacokinetics of the compounds.

The overall analysis from the in silico ADME (absorption, distribution, metabolism, and excretion) study implies that **L09**, **L10**, and **L11** have promising profiles for potential Alzheimer’s treatment. They were prioritized for in vivo testing involving biological assays to confirm their therapeutic activity. This step is crucial for progressing toward drug development.

The Electron Paramagnetic Resonance (EPR) technique is crucial for in vitro examinations to assess copper’s binding affinity with specific molecules.^32,33^ EPR signature analysis determined whether the synthesized ligands could successfully extract Cu­(II) from the Aβ oligomer and the particular quantity of chelator required to achieve this.^34^ For compounds **L03–L08**, the spectral profiles of copper salts mixed with these compounds remained unchanged compared to [Cu­(OH_2_)_6_] (refer to Figure 3b), even with an excess of each compound, indicating no complex formation with Cu­(II). Therefore, **L03–L08** were not studied further in competition with Aβ. Conversely, compounds **L09–L11** exhibited significantly different spectral profiles when mixed with copper salts, compared to the EPR signature of [Cu­(OH_2_)_6_], indicating the formation of Cu-ligand complexes. Consequently, **L09–L11** were subjected to competition studies against Aβ. For these compounds, the spectra of Cu-Aβ-L competition closely resembled the Cu-L spectra, distinct from the Cu-Aβ spectrum (see Figure 3c), illustrating a stronger affinity of Cu­(II) for **L09–L11** over Aβ peptide. **L09** and **L10** extracted nearly all Cu­(II) from Aβ at three equivalents, whereas **L11** accomplished this with just one equivalent.

Based on the results of the in silico and in vitro studies, the new synthesized chelators **L09**, **L10**, and **L11** were selected for the in vivo assays. UV–vis stability tests were performed on the compounds in potential administration solvents. It is suggested that there is greater aqueous stability than in the imines. For **L09** and **L10**, DMSO solutions showed only concentration decay over time, whereas water and phosphate buffer induced spectral changes (degradation/product formation) after 2 and 4 days, respectively. The citrate buffer (used for streptozotocin delivery) was tested briefly for **L10** due to expected imine instability in acidic media; its spectral profile matched aqueous degradation at 28 days, prompting rejection. A 2% DMSO/98% PBS mixture was selected to balance solubility and stability (Figures S34 and S35).

Cellular studies are vital in Alzheimer’s research, particularly concerning drug safety and efficacy. These studies allowed us to investigate the effects of potential therapeutic compounds chosen **L09**, **L10**, and **L11** on neuronal cell lines. By assessing cytotoxicity and identifying possible side effects at the cellular level, we can ensure that new drugs are safe before moving to more complex models or clinical trials. So, in the cellular study, all analyses were performed after 24 h of compound mHippoE-2 cell culture exposure. It was observed that for compounds **L09** and **L10**, only concentrations above 500 μM reduced cell viability to less than 50%. For the compound **L11**, concentrations above 50 μM reduced cell viability to less than 50%. Based on this, the EC_50_ for each ligand was determined (Figure 4a). These data indicate that, up to these concentrations, the ligands are not cytotoxic to the mHippoE-2 cell culture used here as a model of the hippocampus.

**Figure 4:**
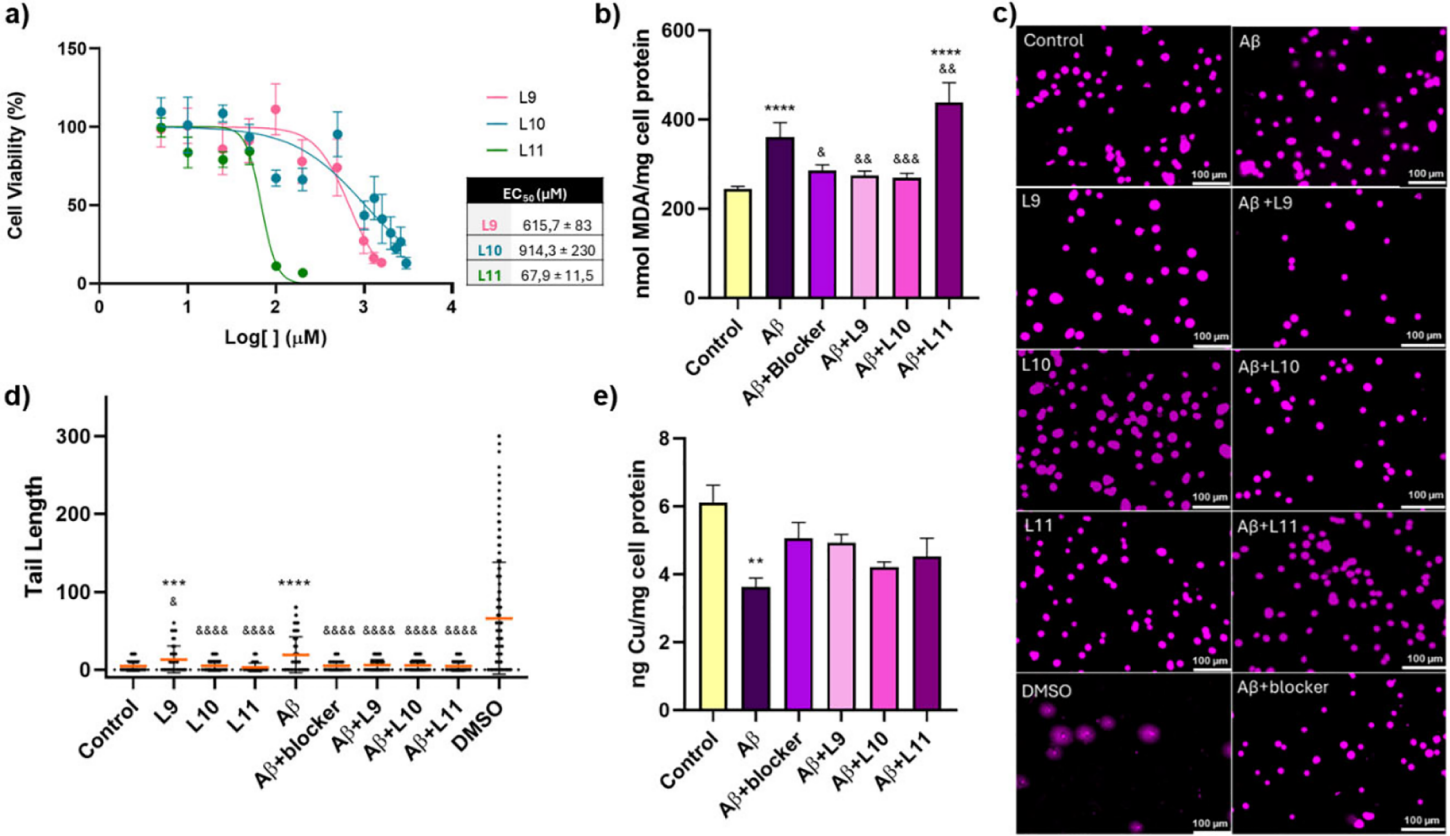
Results of the cellular study with compounds **L09**, **L10**, and **L11** in the mHippoE-2 cell line. All analyses were performed after 24 h of treatment exposure. The concentrations used in the assays after the MTT test were 50 nM of beta-amyloid oligomer, 0.1 μM of amyloid blocker, 500 μM of **L09**/**L10**, 50 μM of **L11**, and 20% DMSO. (a) Cell viability of the mouse hippocampal cell line exposed to different concentrations of the synthesized compounds **L09**, **L10**, and **L11**, with the respective EC_50_ values obtained. (b) Lipid oxidation measurements using malondialdehyde formation as a parameter. Results of Aβ/Aβ+compounds in nmol MDA per mg of protein. (c) Images were obtained from the comet assay using a Leica AF6000 inverted wide-field microscope. (d) Graphical representation of DNA damage represented by Tail Length using 20% DMSO as a positive control. (e) Determination of intracellular copper levels obtained by ICP-MS in studies with Aβ/Aβ+Compounds. ****p < 0.0001, ***p < 0.001, **p < 0.01 compared to the control. &&&&*p* < 0.0001, &&&*p* < 0.001, &&*p* < 0.01, and &*p* < 0.05 compared to Aβ (one-way ANOVA: Turkey’s test, *n* = 4).

Compound L09 exhibited more cytotoxic action than **L10**, as cell viability at 1000 μM was 22% for **L09** and 40% for **L10**. The only structural difference between **L09** and **L10** is that **L09** has a methoxy group, while **L10** has an ethoxy group. Thus, it is observed that increasing the carbon chain reduces cellular cytotoxicity for this group of ligands. The quinoline-based compound (**L11**) was the most cytotoxic among those studied, with an EC_50_ of 67.9 ± 11.5 μM. Cell viability at 100 μM was 11%. Based on these results, concentrations of 500 μM for **L09/L10** and 50 μM for **L11** were selected for subsequent experiments to verify copper uptake from cells and molecular marks of oxidative stress.

Cellular studies also help elucidate how candidate drugs interact with cellular pathways and biological processes involved in AD, such as amyloid-beta accumulation. At a concentration of 50 nM, beta-amyloid oligomer was found to increase lipid peroxidation, which is a marker of oxidative stress and cellular damage (Figure 4b). Both compounds **L09** and **L10**, used at a concentration of 500 μM, were effective in reducing the oxidative damage that was associated with Aβ-treated cells. This effect was significant because the levels of lipid peroxidation in cells treated with these ligands were comparable to those in control, healthy cells, indicating their potential protective role. Contrary to **L09** and **L10**, the **L11** compound increased oxidative damage at a concentration of 50 μM. The effects were statistically different from the control and Aβ groups, suggesting that **L11** might exacerbate oxidative stress rather than mitigate it. These results are essential for understanding potential therapeutic interventions, as they indicate that while some compounds can counteract Aβ-induced oxidative damage, others may worsen it. This highlights the necessity for carefully selecting and testing candidate compounds in developing Alzheimer’s treatments. The comet assay is a technique used to assess DNA damage in individual cells by quantifying strand breaks. The degree of DNA migration in the assay reflects the extent of DNA breakage within the cell. Results are indicated by the Tail Length, measured in pixels, representing the tail length formed by DNA fragmentation. The presence of this tail is evident in the positive control (20% DMSO), as shown in Figure 4c, compared to the positive control and the beta-amyloid oligomer, compounds **L09**, **L10**, and **L11** exhibited minimal DNA damage (Figure 4d). When applied as treatments for the damage induced by beta-amyloid, all studied compounds successfully reversed the DNA damage, bringing it in line with the control conditions (showing no statistical difference from the control). Therefore, these ligands effectively prevented DNA damage caused by beta-amyloid.

The copper levels were determined using ICP-MS (Figure 4e) and expressed as ng of Cu per mg of protein. Treatment with the beta-amyloid oligomer resulted in a significant decrease in copper levels compared to the control. However, the copper levels were restored to those observed in the control when beta-amyloid was combined with either the compounds or the amyloid blocker, a small peptide of 6 amino acid residues (Ac-Gln-Lys-Leu-Val-Phe-Phe-NH_2_), used as a control due to the capability of both binding to the Aβ-peptide and preventing its polymerization into fibrils.^35^

For animal studies, male Wistar rats were used and treated with streptozotocin (STZ) in intracerebroventricular (ICV) injection as a model for AD,^19,36,37^ thus evaluating both biochemical and behavioral changes before and after the treatment with the compounds to determine if they were capable of reversing AD similar symptoms in this model. Following the animal studies, in the immunofluorescence experiments of the rat hippocampus, the CA1, CA3, and dentate gyrus (DG) regions were analyzed, as these are fundamental regions for memory formation, where they are among the areas with the highest copper levels in the entire brain. It was possible to determine the action of compounds **L09**, **L10**, and **L11** on neuroinflammation, oxidative stress, and the regulation of copper levels. In the immunofluorescence analysis (Figure 5a,b), the STZ animal group showed high levels of beta-amyloid and GFAP, indicating that these animals were subjected to a neurodegenerative condition characterized by astrocytic reactivity with significant beta-amyloid plaque accumulation, leading to impaired memory storage and retrieval. Due to ATP7B levels being significantly different from the control, the STZ group exhibited a copper imbalance, revealing that the metabolism of this metal is affected during the degenerative condition induced by STZ. It was observed that groups treated with the compound **L10** displayed a significant reversal of this condition in the CA3 region of the hippocampus (Figure 5c,d,e), with a considerable reduction in the mean intensity of astrocytes, beta-amyloid, and ATP7B. This result demonstrates lower cytotoxicity, neuroinflammation, and oxidative stress in animals, probably due to the chelating effect of copper in the CA3 region. Although the STZ+**L09** and STZ+**L11** groups showed no changes in beta-amyloid and GFAP levels compared to the STZ group, both **L09** and **L11** were able to restore and control ATP7B expression levels to those of the control, suggesting that these compounds actively chelate and modulate hippocampal copper (Figure 5c) levels but without neuroprotective effects. No statistical difference was observed in CA1 and DG in the treatment groups compared to the STZ animal group.

**Figure 5:**
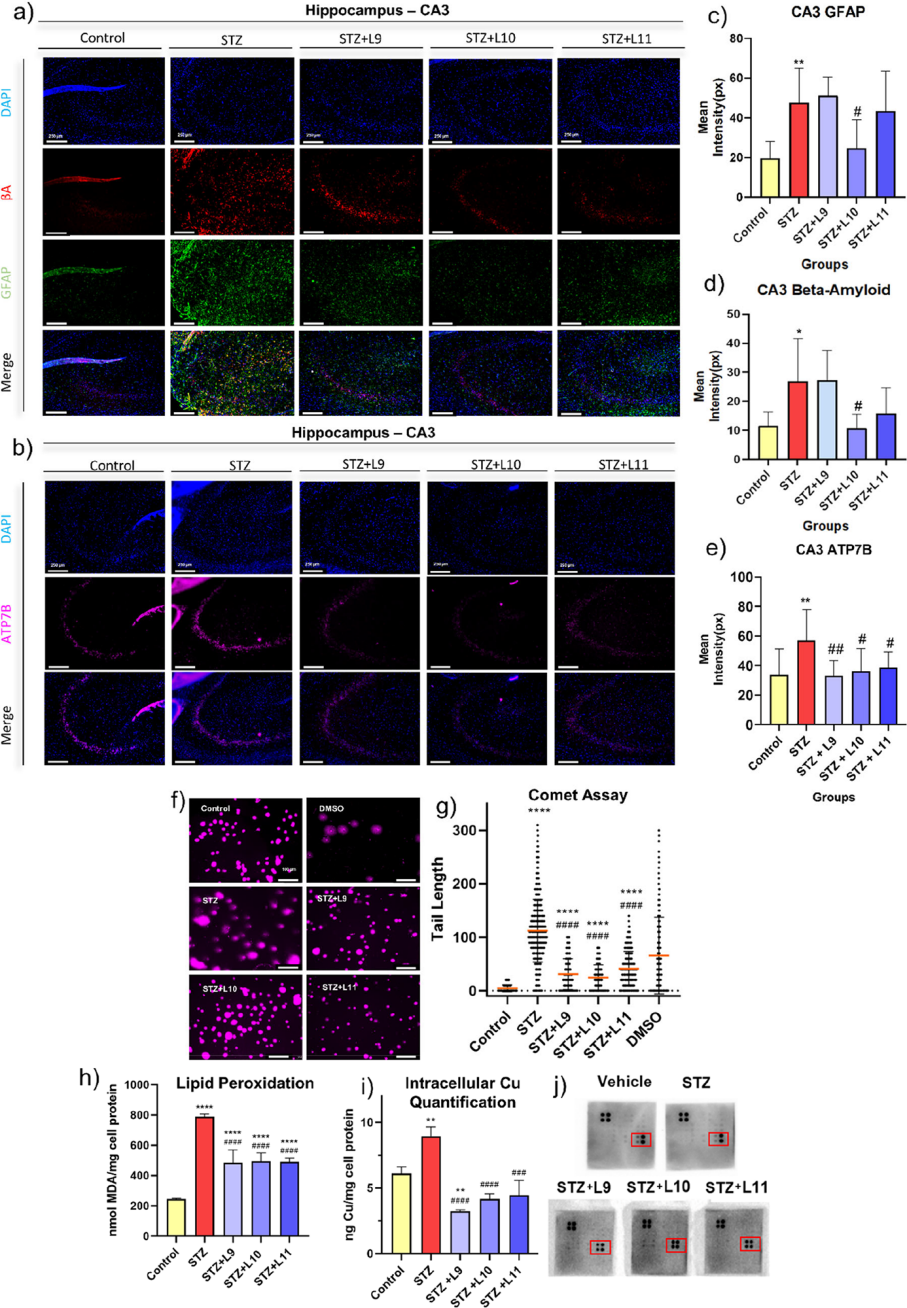
Improvement in the control of neuroinflammation and oxidative stress, regulation of ATP7B levels, and reduced Aβ plaques caused by treatment with the compounds. (a) Representative immunofluorescence images of the Control, STZ, STZ+L09, STZ+L10, and STZ+L11 groups in the CA3 region of the hippocampus for Aβ (red), GFAP (green), and DAPI (blue), and (b) ATP7B (pink) (*n* = 3). (c–e) Signal intensity was quantified using graphs, where the mean fluorescence intensity is represented by bars, with their standard error bars shown. (f) Representative images of the comet assay performed on cells. (g) The graph represents the tail lengths of the comets. (h) The MDA (malondialdehyde) assay assessed oxidative stress levels. (i) Quantification of intracellular copper levels, expressed as ng of copper per mg of cellular protein. (j) Images of the BDNF detection kit. The red areas show the BDNF signal intensity (two on the left) and beta-actin (two on the right). Statistical analysis was performed using Two-Way ANOVA with Bonferroni post hoc. * Represents the statistical difference compared to the control group, and # represents the statistical difference compared to the STZ group. **p < 0.01, ***p < 0.001, ****p < 0.0001, #*p* < 0.05, ##*p* < 0.01, ###*p* < 0.001.

DNA damage caused by STZ in cells was evaluated using the comet assay, revealing longer comet tails in the STZ group (Figure 5f). All treated groups reduced DNA damage, though not sufficiently to return to control levels. One contributing factor to DNA depletion is oxidative stress. Thus, malondialdehyde (MDA) levels, an indicator of lipid peroxidation, were measured (Figure 5h). Similarly, all treatment groups significantly reversed the effects of STZ but did not fully restore control levels.

In the ICP-MS analysis of cells (Figure 5i), the STZ group exhibited excessive intracellular copper accumulation compared to the control, reflecting disrupted copper metabolism during the action of STZ in treated cells. In contrast, all treated groups showed reduced copper levels, where these findings suggests that the ligands can modulate copper homeostasis in hippocampus cell culture (Figure 5i), acting in a way that removes copper from the cell and returns it to the extracellular space.

The results of protein expression analysis conducted on the rats’ hippocampus revealed high sensitivity in areas enriched with Brain-Derived Neurotrophic Factor (BDNF). BDNF is a vital neurotrophic factor crucial for neuroplasticity and the formation of new memories, and it plays a significant role in promoting the growth and survival of neuronal cells.^38,39^ Within the study, the group of rats treated with Streptozotocin (STZ) displayed a noticeable reduction in BDNF signal intensity compared to the control group. This reduction suggests a compromised level of neurotrophic support, which could impair cognitive functions and neuronal health. Interestingly, the administration of **L11** in animals resulted in a partial restoration of BDNF levels, indicating some improvement in neurotrophic conditions, although not fully. In contrast, treatment with **L09** exhibited minimal effect, barely altering the BDNF levels. Significantly, treatment with **L10** led to a substantial increase in BDNF expression. This enhancement ensured that the memory functionality in these rats was not disrupted by the STZ-induced damage, consequently promoting better neuronal survival. Throughout the study, beta-actin served as an internal control to monitor and confirm the consistency of experimental conditions. It remained stable across all groups, affirming that the observed changes in BDNF expression were specific and not due to experimental variability. This consistency underpins the reliability of the results detailed in Figure 4j, highlighting the differential impact of the treatments tested.

The Barnes Maze is a widely used experimental tool in neuroscience to assess spatial learning and memory in rodents, primarily mice and rats, and it is valued for its ability to provide insights into the neurological mechanisms underlying spatial navigation, learning, and memory. This maze is especially valuable for studying the effects of genetic modifications, pharmacological treatments, or neurological diseases on cognitive functions in animal models.^40,41^ The maze consists of a circular platform with multiple holes around its perimeter. One of these holes leads to an escape box or a safe area, representing the target for the animal. During trials, the animal is placed in the maze’s center and encouraged to find the escape hole using spatial cues around the testing room. These cues serve as landmarks that the animal must learn to navigate effectively to locate the escape box. Initially, during the acquisition phase, the animal explores the maze, gradually learning to associate specific spatial cues with the location of the escape hole. Over subsequent trials, the time taken to find the escape box is recorded. As the animal learns, the time it takes to locate the target generally decreases, reflecting improvements in spatial learning. After the acquisition phase, a probe trial is often conducted. The escape box is removed in this test, and the animal’s search strategy is observed. The focus during this trial is on the amount of time the rodent spends near the previously correct hole, which indicates its memory recall abilities. Here, as a final evaluation of the effectiveness of the three compounds selected (**L09**, **10**, and **11**) from in vitro and in vivo studies, we performed the Barnes Maze records and analysis to conclude the study.

In the analysis of learning curves (Figure 6a), escape latencies were evaluated over 4 days of rat training after 1 month of STZ or STZ/compound treatment, comparing groups of administered compounds via intraperitoneal (IP) injection and oral gavage. In the upper graph (IP-treated groups), it was observed that initially, the STZ+**L10**, STZ+**L11**, and control groups (Sham animals, only with ICV injection of buffer) exhibited similar latencies. In contrast, the STZ and STZ+**L09** groups showed reduced performance. By the end of the training period, all groups achieved equivalent latencies, suggesting a potential improvement in memory recall during the initial trials for the IP compounds-treated groups. In the lower graph (gavage-treated groups), all groups performed worse than their respective IP groups. The STZ+**L10**, STZ+**L09**, and STZ+**L11** groups displayed high escape latencies, close to those of the STZ group, while the control group maintained superior performance, albeit lower than the IP controls. These results suggest that the administration method directly influences learning and spatial memory in the animals, with the IP route showing a more significant therapeutic potential than gavage for these compounds. The entire experimental design, including treatment stages, Barnes maze training, and probe trials, was conducted as outlined (Figure 6b).

**Figure 6:**
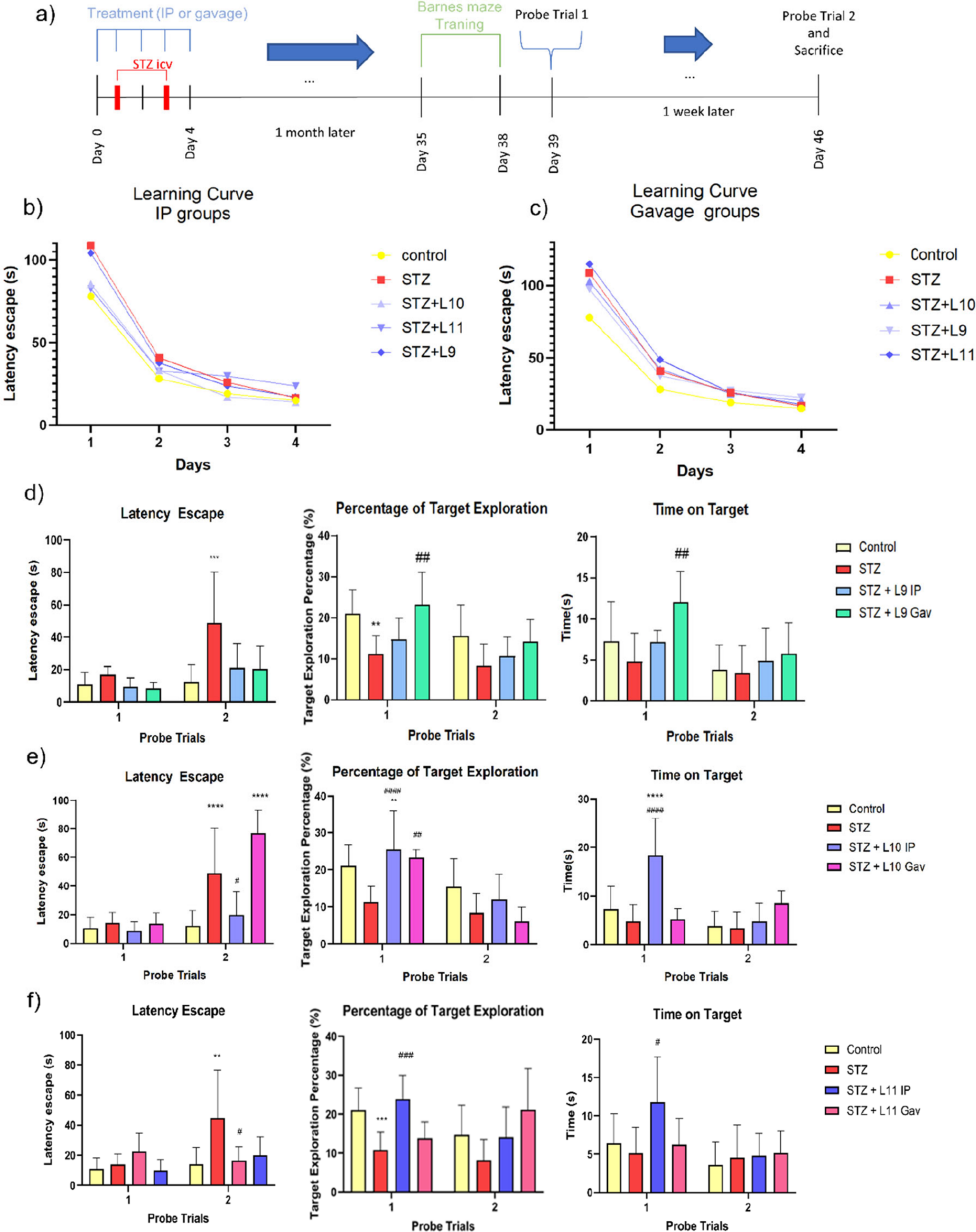
Treatment with the compounds reverses animal spatial memory decline. (b, c) Escape time during training days for the IP and gavage groups. (a) Experimental overview of treatment administration days, STZ application, and behavioral testing. (d–f) Graphs of probe trials 1 and 2, including escape time, percentage of target hole exploration, and time in the target zone. Statistical analysis was performed using Two-Way ANOVA with Bonferroni post hoc. * Represents the statistical difference compared to the control group, and # represents the statistical difference compared to the STZ group. **p < 0.01, ***p < 0.001, ****p < 0.0001, #*p* < 0.05, ##*p* < 0.01, ###*p* < 0.001.

On the first day of the Probe Trial, comparisons were made between the control and STZ groups for the parameters of escape time, target exploration, and time spent in the target zone, using the two proposed compound treatment routes: gavage and IP (Figure 6c). For escape time, no differences were observed between the groups on the first probe trial, which aligns with the findings from the learning curve (Figure 6a), where escape times were statistically equal for all groups by day 4 of training. However, a statistical difference was noted between the control and treatment groups compared to the STZ group for the parameters of time spent in the target zone and percentage of target exploration. On the second day of the probe trial, the opposite was observed: a statistical difference was found between the control and treatment groups compared to the STZ group for escape time, but not for the other parameters. When analyzing the treatment routes, it was observed that STZ+**L10** Gav and STZ+**L11** Gav were unable to reverse the memory impairment caused by STZ, while the IP administration of these compounds showed promise. In contrast, STZ+**L09** Gav demonstrated a more positive influence on memory preservation and recall than its IP administration.

## Discussion

The novel copper chelators investigated in this study exhibit a different mechanism of action and potential advantages compared to existing Alzheimer’s drugs, primarily acetylcholinesterase inhibitors (AChEIs) and memantine.^42,43^ Acetylcholinesterase Inhibitors (AChEIs) (e.g., donepezil, rivastigmine, galantamine) inhibit the breakdown of acetylcholine, a neurotransmitter crucial for memory and cognitive function.^44^ They provide symptomatic relief, primarily improving cognitive function in mild to moderate Alzheimer’s, but do not address the underlying disease pathology.^2,45^ Memantine is an *N*-methyl-d-aspartate (NMDA) receptor antagonist. It modulates the effects of glutamate, another neurotransmitter involved in memory and cognition. It is typically used in moderate to severe Alzheimer’s, often in combination with AChEIs.^3,46^ Side effects can include dizziness, headache, and constipation.^46^ Nowadays, we have monoclonal antibodies (e.g., lecanemab, aducanumab) as newer drugs that target amyloid-beta plaques in the brain, aiming to slow disease progression. They show some promise in reducing amyloid plaques but have significant side effects (brain swelling and bleeding) requiring close monitoring. Their effectiveness remains a topic of ongoing debate.^5,47,48^

Furthermore, it is worth emphasizing that monoclonal antibodies exhibit various adverse effects. Still, the key issue is that their therapeutic efficacy has proven to be quite limited, even though they can significantly reduce beta-amyloid levels.^5,49^

Herein, we propose that copper chelators work by modulating copper homeostasis in the brain. It is known that excess copper contributes to amyloid-beta plaque formation and oxidative stress, both implicated in Alzheimer’s pathogenesis.^13,27,50,51^ By chelating (binding) excess copper, these compounds aim to address the underlying disease mechanisms rather than just treating symptoms. They could offer disease-modifying potential rather than just symptomatic relief if proven effective. They might also avoid the side effects associated with AChEIs, memantine, or monoclonal antibodies.

This study offers a different approach by focusing on copper control as a therapeutic target. It investigates copper chelation, a less-explored mechanism in Alzheimer’s treatment, potentially offering a new avenue for disease modification beyond targeting amyloid-beta or tau.^13,52,53^ The rationale is grounded in the fact that excess copper contributes to amyloid-beta aggregation,^54,55^ oxidative stress,^56,57^ and neuroinflammation^58,59^ —key players in Alzheimer’s pathology. Combining in silico absorption, distribution, metabolism, and excretion (ADME) predictions and in vitro and in vivo efficacy testing facilitates rational drug design. This approach helps identify and refine compounds with improved therapeutic properties and reduced side effects. These predicted favorable ADME properties for the most promising compounds (**L09**, **L10**, and **L11**), suggesting suitability for oral administration and blood-brain barrier permeability, an essential feature for effective central nervous system (CNS) drug delivery.

Using Electron Paramagnetic Resonance (EPR) spectroscopy, we demonstrated that **L09**, **L10**, and **L11** effectively extracted copper from the Cu-β-amyloid complex among all 9 compounds. This shows a direct impact on a key aspect of Alzheimer’s pathogenesis. Furthermore, these compounds showed low cytotoxicity at therapeutically relevant concentrations, suggesting good safety profiles. They reduced lipid peroxidation and DNA damage induced by beta-amyloid.

As indicated by EPR analysis, no copper complex is formed with compounds **L03–L08**. According to Pearson’s theory, nitrogen has a greater affinity for copper than oxygen, although chelation with oxygen also occurs. Furthermore, chelation resulting in structures with 5- or 6-membered rings is more favorable due to the stability of these rings, as are chelators that can provide a square-planar or tetrahedral geometry for Cu­(II), most commonly adopted for the metal ion.^19^ To achieve a square planar or tetrahedral geometry for Cu­(II) would require a 1:2 (M:L) stoichiometry for **L9** and **L10**, while chelation with **L11** can already provide this geometry with a 1:1 (M:L) stoichiometry, which possibly explains why **L11** can remove the metal ion from Aβ with 1 equiv, while **L9** and **L10** need more equivalents. Chelators **L09**, **L10**, and **L11**, unlike the others, have nitrogen atoms available to chelate copper in strategic positions to form a 5-membered ring with the metal. Furthermore, Table 1 shows that **L09**, **L10**, and **L11** have a significantly lower number of rotatable bonds, indicating that they are more rigid structures compared to **L03–L08**. Possibly, in this case, the lower flexibility may favor a more defined conformation, with the binding sites ideally positioned and organized for chelation. Chelators **L09–L11**, therefore, share some structural features not observed for compounds **L03–L08**, which appear to be important for complex formation with Cu­(II).

A streptozotocin (STZ)-induced Alzheimer’s rat model was utilized to evaluate the in vivo efficacy of **L09**, **L10**, and **L11**. In the Barnes maze test, the imine **L10** demonstrated the most significant improvements, including reduced neuroinflammation, oxidative stress, restored copper homeostasis, and improved spatial memory performance. **L09** and **L11** showed less impact, highlighting the importance of careful structural optimization in drug design.

It is important to highlight one of the key findings of our research: the alteration of ATP7B in STZ-treated CA3 and the ability of chelators to restore its levels to those of the control group. Squitti et al. have previously demonstrated that genetic variations in Alzheimer’s patients are associated with ATP7B mutations.^60^ Additionally, there is growing consensus in the literature that Alzheimer’s disease may present distinct subtypes.^61^

Concomitantly, all tested compounds induced changes in ATP7B levels, specifically in CA3, with no significant effects in other hippocampal regions. CA3 is a critical structure for pattern completion and associative memory,^62^ which in the Barnes maze, is responsible for linking visual cues on the walls to the correct escape location.^63,64^ Thus, the observed ATP7B dysregulation and reduced beta-amyloid levels in this region may directly impact this process. This explains why the parameters measured during the first and second probe trials did not exhibit a universal improvement pattern. Although spatial memory improvement was detected, overall maze performance remained impaired, as neuroinflammation and beta-amyloid toxicity were alleviated only in CA3, with no effects in other hippocampal subregions (CA1 and DG), which are essential for spatial navigation.

Regarding the structure–activity relationship, although quinoline compounds are already extensively studied as treatments for Alzheimer’s disease, imines have the potential to provide better results. **L10** was able to inhibit memory loss and the overexpression of GFAP and Aβ in an STZ model of the disease. Regarding imine chelators, the substituent group also directly impacts the pharmacological activity of the compounds. The insertion of an ethoxyl group in place of a methoxyl group significantly increased the efficacy of **L10** compared to **L09** in in vivo assays. Modifying substituent groups alters a molecule’s physicochemical characteristics, directly impacting its ADME profile. For membrane permeability, a Log *P* value near 3 is often considered optimal. L10s Log *P* value is 0.42 units higher than that of L9, making it approximately 2.6 times more lipophilic and placing it closer to the optimal value. This increased lipophilicity suggests L10 has superior permeability, which could enhance its oral absorption and blood–brain barrier penetration, potentially leading to improved efficacy in in vivo models. In conclusion, this study presents a promising new direction in Alzheimer’s treatment, focusing on copper chelation. Combining in silico, in vitro, and in vivo studies, its multifaceted approach strongly suggests that specifically designed copper chelators could offer a novel, effective, and well-tolerated therapeutic strategy. However, further research and clinical trials are critical before these compounds can become a viable treatment option for Alzheimer’s disease.

## Experimental Procedures

### General Procedure for the Synthesis of Imines L03–L08

The respective aldehyde (2 equiv) dissolved in ethanol was added to a round-bottom flask. Then, the respective amine (1 equiv) was added, and the reaction continued with stirring overnight at room temperature. The products were obtained in solid or oily forms. The synthetic details are described in the Supporting Information.

### General Procedure for the Synthesis of Imines L09 and L10

In a round-bottom flask fitted with a Dean-Stark apparatus were added the respective aldehyde (1 equiv) and 2-(pyridin-2-yl)­isopropyl amine (1 equiv) were added in toluene. The reaction was heated at reflux until no further water was collected in the trap. Then, evaporation of the solvent on a rotary evaporator yielded the product as an oil. The synthetic details are described in the Supporting Information.

### General Procedure for the Synthesis of Quinoline L11

The synthesis of L11 was achieved through a five-step^29^ sequence starting with the reaction of 3,5-dichloroaniline with acetaldehyde in HCl (0 → 80 °C) to form 5,7-dichloro-2-methylquinoline (2), followed by nitration with fuming HNO_3_/H_2_SO_4_ (0 °C → RT) to afford 5,7-dichloro-2-methyl-8-nitroquinoline (3). Subsequent oxidation of the methyl group using FeCl_3_/K_2_S_2_O_8_ in DMA at 110 °C yielded 5,7-dichloro-8-nitro-2-vinylquinoline (**4**), which was then coupled with 2-picolylamine in dioxane/K_2_CO_3_ to give 2-(5,7-dichloro-8-nitroquinolin-2-yl)-N-(pyridin-2-ylmethyl)­ethan-1-amine (**5**). Finally, the reduction of the nitro group with Fe/AcOH in EtOH under reflux produced L11. All intermediates were purified by silica gel chromatography and the final compound was isolated as a greenish oil. The synthetic details are described in the Supporting Information.

### Nuclear Magnetic Resonance Spectroscopy (NMR)

Nuclear magnetic resonance (NMR) spectra were recorded on a Varian INOVA spectrometer, operating at 500 MHz (^1^H) and 125 MHz (^13^C), using CDCl_3_ or DMSO-d6 as solvent and TMS as internal standard. Chemical shifts (δ) are given in ppm, with coupling constant (*J*) in Hz.

### High Resolution Mass Spectrometry (HR-MS)

For mass spectrometry analysis, a 1 mL sample of the compounds (1 mg/1 mL) solubilized in MeOH was analyzed via high-resolution (5000) UPLC-MS analysis by direct infusion. The UPLC-MS analysis was performed using a UHPLC (Acquity Waters) coupled to a mass spectrometer (Q-ToF microTM Micromass, Waters) using electrospray ionization (ESI). The mobile phase was composed of H_2_O:MeOH 1:9 with 0.1% formic acid (FA).

### In Silico ADME Properties Evaluation

The in silico ADME properties of ligands L03–L11 were calculated through the SwissADME platform (http://www.swissadme.ch/).^30^ Chemical structures of the respective compounds were drawn in a molecular sketcher interface and converted to SMILES notation. Bioavailability radar, physicochemical properties, pharmacokinetic parameters, and medicinal chemistry alerts were observed. It is important to note that SMILES input exclusively considers the relative stereochemistry of each tested compound, and the calculated values are the same for two enantiomers.

### Ultraviolet/Visible Spectroscopy (UV/vis)

Electronic spectra in the Ultraviolet/Visible (UV/vis) region were obtained using a Shimadzu UV-1800 spectrophotometer, employing quartz cuvettes with a 1.000 cm path length. For stability tests, the compound solutions were prepared at a concentration of 150 μM in DMSO, distilled water, 10 mM phosphate buffer pH 7.2, and 50 mM citrate buffer pH 4.5. These solutions were read on the UV–vis spectrophotometer daily for 1 week and then every 7 days until a month had passed. Measurements were performed from 190 to 800 nm.

### Cell Culture

The immortalized hippocampal mHippoE-2 cell line were cultured in high glucose Dulbecco’s Modified Eagle’s Medium (DMEM; Gibco/Life Technologies, Grand Island, NY, USA) and supplemented with 10% fetal bovine serum (FBS; Gibco/Life Technologies), 100 U/mL penicillin and 10.0 mg/mL streptomycin (Sigma-Aldrich, St. Louis, MO, USA). Cell cultures were maintained at 37 °C in a controlled atmosphere of 5% CO_2_ (cell culture incubator—Forma Series II, Hepa Class 200, Thermo Scientific). Culture flasks with a confluence of 80% cell growth were plated according to the density required for each assay.

### Preparation of Aβ1–42 Oligomers

The neurodegenerative condition was induced with β-amyloid protein oligomers of the toxic peptide segment 1–42 (AβO) at a concentration of 50 nM (in DMEM culture medium) for cell lines, with an exposure time of 24 h. The oligomers were prepared according to the protocol described by Stine and collaborators.^65^ The method consists of the solubilization of the commercial aliquot of oligomeric fragments 1–42 of the β-amyloid protein in Hexafluoroisopropanol (HFIP), in sequence, a resting time of 30 min, and after drying the aliquots in the desired concentration with argon gas. After the procedure, the aliquots were stored at −80 °C and thawed just before the cell incubation.

### Storage and Dilution for Amyloid Blocker Solution

The Amyloid Blocker (Sigma-Aldrich SCP0017) solution was stored in aliquots at −20 °C and subsequently diluted in a culture medium. The aliquot added to the cell culture medium should reach a final concentration of 0.1 μM.

### Cytotoxicity Evaluation

Cell viability was determined by MTT assay. mHippoE-2 cells were cultured and plated in 96-well plates at densities of 4.0 × 104 cells/cm^2^ and exposed to treatments with ligands at final concentrations of 5, 10, 20, 50, 100, 200, 500, 1000, 1300, 1600, and 2000 μM for 24 h. The compounds were diluted in DMSO/H_2_O so that the final concentration of DMSO in cells would not exceed 2%, including the control. After an incubation time of 24 h, 30 μL of a 5 mg/mL solution of MTT salt (3-(4,5-dimethylthiazol-2-yl)-2,5-diphenyltetrazole bromide) was added directly to the medium culture and incubated for 45 min in the dark. After this time, the culture medium was removed entirely, and 150 μL of DMSO was added for incubation with agitation and sheltered from light for 15 min. Then, the readings of the samples were performed in a microplate reader (CELER Polaris) at a wavelength of 570 nm. From the absorbances found, the percentage of viable cells was calculated using the negative control as 100% viability. Each treatment concentration was tested in quintuplicate.

### Comet Assay

Comet assay was performed by the method described by Collins et al.^66^ Cells (1 × 10^5^) were plated on 24-well plates and exposed to treatments with ligands at EC_50_ concentration; DMSO 20% was used as a positive control. The treated cells were mixed with 100 μL of 1.0% low-melting-point agarose. The cell suspension was layered on the frosted side of fully frosted slides and kept on ice for solidification of agarose. Then, the slides were kept in lysis buffer for 1 h and then transferred to alkaline buffer for 20 min. Electrophoresis was conducted for 30 min at 19 V and 300 mA. Then, the slides were washed with 0.4 M Tris (pH 7.5), and were distributed to a tank with pure ethanol for 10 min for fixation. SYBR Gold was used for DNA staining at a 1:10,000 dilution in TE buffer. Images of 100 randomly selected cells (50 cells from each of the two replicate slides) were analyzed by a fluorescent microscope, and the comet parameter tail length (TL) was analyzed by ImageJ software with the plug-in OpenComet.

### Determination of Metal Levels—Inductively Coupled Plasma Mass Spectrometry (ICP-MS)

For the determination of intracellular metal levels, the inductively coupled plasma mass spectrometry (ICP-MS) was used, according to the protocol described by Lago et al.^67^ The cell lines were plated at a density of 4 × 10^4^ cells/cm^2^ in 75 cm^2^ flasks and after 24 h of ligand treatment, the cells were trypsinized, washed with PBS (an aliquot was separated for protein quantification by the Lowry method) and freeze-dried. Then, the samples (in triplicate per cell line) were predigested with 50 μL double-distilled concentrated (65% w/v) HNO_3_ for 24 h at room temperature (25 °C). After that, the samples were heated in a water bath at 90 °C for 2 h. After, 950 μL of ultrapure water (Milli-q RiOs D, Millipore) was added, homogenized and heated in a water bath at 90 °C for 2 h. All analytical standard calibration curves and blanks were made by serial dilution of stock standard solutions in 5% v/v HNO_3_. Finally, the samples were analyzed by an inductively coupled plasma mass spectrometer. The results were converted using the protein concentration of each sample and the dilution factor, yielding the result in ng/metal per mg of sample protein. Samples were analyzed by ICP-MS (Agilent 7900, Hachioji, Japan). The results were converted using the protein concentration of each sample and the dilution factor, yielding the result in ng/metal per mg of sample protein. The efficiency of the analysis was evaluated by the recovery of reference material SRM 1643f for the 98.02% by ^63^Cu. The limit of detection was calculated based on the mean concentration (MC) and standard deviation (SD) for each element from 10 different blanks from sample preparation. The instrumental limit of detection (iLOD) was calculated as MC + 3 × SD. The instrumental limit of quantification (iLOQ) was calculated as 3.3 x iLOD, respectively.^68^

### Lipid Peroxidation

The degradation of lipids was determined by measuring malondialdehyde (MDA), which is the end product of lipid peroxidation, using the thiobarbituric acid reactive substance (TBARS) assay.^69,70^ mHippoE-2 cells that had been plated and incubated for 24h in the presence or absence of the ligands under the above-mentioned conditions were washed with 0.9% NaCl, resuspended in 1 mL of 50 mM potassium phosphate buffer (pH 7.4) and frozen (−20 °C) overnight. Cells were subsequently thawed, collected using a cell scraper, sonicated for 10 s and transferred to Falcon tubes. The TBARS assay was carried out by adding 1.0 mL of 20% (w/v) trichloroacetic acid that contained 0.8% (w/v) TBA to each tube and boiling the mixture for 45 min. After cooling to room temperature and centrifugation (250–300 × *g*, 5 min), the absorbance of the supernatant at 535 nm was recorded. By considering the molar extinction coefficient of the MDA-TBA complex to be 1.49 × 105/M·cm,^71^ the amount of TBARS was calculated in terms of mol MDA equivalents that were formed per mg of cell protein.

### Electron Paramagnetic Resonance Spectroscopy (EPR)

Electron Paramagnetic Resonance (EPR) spectra were obtained on a Bruker EMX 10-2.7 Plus spectrometer, operating at a microwave frequency of 9.33 GHz, using a 20 mW microwave power and 4 mm quartz tubes. Experiments were conducted at 77 K using a liquid nitrogen cryostat. EPR samples were prepared from stock solutions of ligands and Cu^2+^ diluted to 1.5 mM in DMSO and water, respectively. In the EPR tube, 1 equiv of Cu^2+^ was mixed with the chelating agent, and 10% glycerol was added as a cryoprotectant to obtain Cu-L spectra. EPR samples were prepared for competition experiments from stock solutions of ligands diluted to 200 μM in DMSO. One equivalent of oligomer and Cu^2+^ was added, as well as 10% glycerol, to obtain Cu-L-Aβ spectra. More equivalents of ligands were added as needed until it was sufficient to start removing copper from the Aβ oligomer.^34^ Before EPR competition experiments, Aβ1–42 oligomers were prepared following the protocol described by Stine et al.^65^ and aliquots, at the desired concentration, were stored at −80 °C and thawed immediately before analysis.

### Immunofluorescence

Immunofluorescence images were obtained using a Leica AF6000 inverted wide-field microscope. For each histological slide subjected to immunofluorescence analysis, photos of the dentate gyrus (DG), CA1, and CA3 regions of the hippocampus were obtained (*n* = 3). Antigen retrieval was performed with 0.3% sodium citrate solution at pH 6.0 for 1 h at 60 °C. After 3 washes with PBS, slides were blocked with 5% BSA in 0.3% Triton X-100 for 40 min. Then, primary antibodies Anti-GFAP (G3893 Sigma-Aldrich, 1:200) and Anti-Aβ1–42 (BS-0107R Bioss, 1:100) or Anti-ATP7B (SAB4502224 Sigma-Aldrich, 1:100) were incubated for 12 h in the same blocking solution. After 12 h, following washes with PBS, secondary antibodies Alexa Fluor 488 (Anti-Mouse, 1:1000) and Alexa Fluor 647 (Anti-Rabbit, 1:1000) were incubated for 2 h in 0.3% Triton X-100 solution with DAPI 1:1000. After the incubation period and washes, slides were mounted with coverslips, visualized, and stored in the refrigerator. The entire procedure was repeated for the negative control without primary antibody incubation. Immunofluorescence images were processed using LAS X Office software. For image quantification, images were extracted from LAS X software without any brightness or contrast adjustments and analyzed using ImageJ software.

### Protein Expression

The expression of neuronal-related proteins was investigated by semiquantitative detection of 19 human neuronal proteins on a Rat Neuro Discovery Array C1 (RayBiotech; Norcross, GA, USA). Briefly, after lysis, each sample was incubated on the membranes with the primary antibody at 4 °C. The following day, each membrane, after repeated washes with Wash Buffers I and II (provided with the kit), was incubated with a cocktail of secondary antibodies for 2 h at room temperature. Then, after washing with the aforementioned Wash Buffers, the membranes were incubated with HRP for 2 h at room temperature. After additional washes, each membrane was incubated with a detection buffer and chemiluminescence was detected using a ChemiDoc MP imager. The intensity of the protein array signals was analyzed using IMAGE LAB software and each protein spot was normalized against Positive Controls printed on each membrane. Data analysis was conducted following the protocol instructions. Raw densitometry data were subtracted from the background (negative control signals) and normalized to the positive control signals

### Animals and Housing

Male Wistar rats (2 months old; weight 250–300 g) were obtained from ICB-USP (São Paulo, SP, Brazil) and housed in white PVC (40 × 33 × 17 cm) in a room with controlled temperature (24 °C), 40–55% relative humidity, 12/12 light–dark cycle and free access to fresh water and food. All procedures were approved by and are following the local ethics committee from the Universidade Federal do ABC (CEUA/UFABC, protocol number 5751061119).

### Surgery and Injection Procedure

For cannula implantation into the ventricle, rats were anesthetized with isoflurane (4% for induction, 1–2% for maintenance with 0.8 mmHg O_2_). The top of the animal’s head was shaved and cleaned with povidone-iodine solution. Lidocaine was injected into the top of the skull, and after 5 min, an incision (3–4 cm) was made with a scalpel. The top of the skull was cleaned with hydrogen peroxide. The cannula was fixed with stereotaxic coordinates for unilateral intracerebroventricular injection: 0 mm posterior to bregma, 1.4 mm lateral to the sagittal suture, and 3.5 mm below the cerebral surface.^72^ After the surgical procedure, the animals rested for 5 days. After this period, they received two rounds of ICV injections, the first on day 1 and the second on day 3 of the experiment. Control group rats received 4 μL of 50 mM citrate buffer, pH 4.5. The STZ group received 4 μL of 3 mg/kg STZ freshly dissolved in citrate buffer.^73,74^ Treated animals received STZ injections as per the STZ group, and treatment administration was performed via intraperitoneal (i.p.) or oral (by gavage) routes, once a day, from day 1 to day 5, 12.5 mg/kg in 700 μL of the respective chelator freshly dissolved in 98% PBS 10 mM and 2% DMSO.^19^ During the ICV injection procedure, animals were anesthetized to minimize stress.

### Barnes Maze Behavior Test

The protocol used for the Barnes maze experiment was adapted from three sources.^73,75,76^ The experiment was performed in the fourth week after injection on a circular platform (120 cm diameter) with 20 peripheral holes (10 cm diameter) evenly spaced around the perimeter. In the experimental room, visual clues were located on every wall. A dark escape box was positioned under the target hole. The acquisition phase took place over 4 days, with four trials per day and a minimum 15 min intertrial interval. Before each trial, the animals were placed inside a dark cylinder on the platform for 30 s. The stopwatch was initiated when the cylinder was removed. The platform was cleaned with alcohol before each trial. During each trial, the animals had 3 min to explore the holes and platform while an aversive sound was playing (1550 Hz, 80–90 dB). If the animals entered the escape box, the sound was stopped. If the rat did not enter the box within 3 min, it was led gently to the target hole. On the fifth day, a probe trial was performed where the escape box was removed from the platform, and the maximum exploration time was reduced to 90 s. In the acquisition phase, escape latency, roamed distance, and average speed were analyzed. In the probe trial, the quadrant from which the quadrant, and the percent of quadrant time, the percent of target exploration, elapsed time to target, time spent at target, and distance were counted. Videos were recorded using EthoVision XT (Noldus, Leesburg, VA, USA) and analyzed with Kinovea Software version 9.5. During the intracerebroventricular injections (icv) procedure, the animals were anesthetized to minimize stress. Any wounds that occurred after ICV were treated with veterinary care.^77^

### Statistical Analysis

Each set of biochemical and behavioral assays was performed in triplicate (*n* = 3) and decuplicate (*n* = 10), respectively. All treated samples were compared to their respective negative and positive controls using Analysis of Variance (ANOVA) with Bonferroni post hoc testing. Analyses were conducted using GraphPad Prism software (Version 8.0.1), with the obtained assay means evaluated for statistical significance and standard deviation (±SD). Significance thresholds were set at *p* ≤ 0.05 (*), *p* ≤ 0.01 (**), *p* ≤ 0.001 (***), and *p* ≤ 0.0001 (****) for differences between experimental samples.

## Supplementary Material



## Notes

Spectroscopic data (^1^H and ^13^C NMR and HR-ESI-MS) of the obtained compounds **L03–L11**; immunofluorescence panels with GFAP, Aβ, and ATP7B markings in the CA1, CA3 and DG regions of the hippocampus of rodents in the control, STZ and chelator-treated groups; panels containing the negative control of the immunofluorescence markings; bioavailability radars obtained in the in silico tests of compounds **L03**–**L11**; and stability test of **L09** and **L10**, in different solvents, in UV–vis (PDF)
