# Supplementary material for: Novel Copper Chelators Enhance Spatial Memory and Biochemical Outcomes in Alzheimer’s Disease Model
Source: ACS Chem Neurosci. 2025 Aug 15;16(17):3267–81. doi: 10.1021/acschemneuro.5c00291 (PMC12412111; doi:10.1021/acschemneuro.5c00291)
Supplement: Supplementary file 1 [file cn5c00291_si_001.pdf]

Supplementary Information

Novel Copper Chelators Enhance Spatial Memory and Biochemical Outcomes in Alzheimer's Disease Model

Mariana L. M. Camargo[1], Augusto B. Farias[1], Giovana B. Bertazzo[1], Rafael N. Gomes[1], Kaio S. Gomes[1], Lucas M. Bosquetti[1], Silvia H. Takada[2], Felipe C. Braga[3], Caroline C. Augusto[1], Bruno L. Batista[1], Kleber T. de Oliveira[3], and Giselle Cerchiaro[1*]

[1]Metal Biochemistry and Oxidative Stress Laboratory. Center for Natural Sciences and Humanities, Federal University of ABC – UFABC, Santo André, São Paulo, Brazil, 09210-580. *Corresponding author: giselle.cerchiaro@ufabc.edu.br Tel.+55 11 49960160

[2]Laboratory of Neurogenetics. Center for Mathematics, Computing and Cognition, Federal University of ABC – UFABC, São Bernardo do Campo, São Paulo, Brazil, 09606-045.

[3]Departament of Chemistry, Federal University of São Carlos – UFSCar, São Carlos, São Paulo, Brazil, 13565-905.

Index of figures

Figure S1: ^{1}H NMR Spectrum of L03 (500 MHz, $CDCl_3$)....................S13

Figure S2: ^{13}C NMR Spectrum of L03 (125 MHz, $CDCl_3$) S13

Figure S3: HR-ESI-MS Spectrum of L03....................S14

Figure S4: ^{1}H NMR Spectrum of L04 (500 MHz, $CDCl_3$)....................S14

Figure S5: ^{13}C NMR Spectrum of L04 (125 MHz, $CDCl_3$) S15

Figure S6: HR-ESI-MS Spectrum of L04....................S15

Figure S7: ^{1}H NMR Spectrum of L05 (500 MHz, $CDCl_3$)....................S16

Figure S8: ^{13}C NMR Spectrum of L05 (125 MHz, $CDCl_3$) S16

Figure S9: HR-ESI-MS Spectrum of L05....................S17

Figure S10: ^{1}H NMR Spectrum of L06 (500 MHz, $CDCl_3$)....................S17

Figure S11: ^{13}C NMR Spectrum of L06 (125 MHz, $CDCl_3$) S18

Figure S12: HR-ESI-MS Spectrum of L06....................S18

Figure S13: ^{1}H NMR Spectrum of L07 (500 MHz, $CDCl_3$)....................S19

Figure S14: ^{13}C NMR Spectrum of L07 (125 MHz, $CDCl_3$) S19

Figure S15: HR-ESI-MS Spectrum of L07....................S20

Figure S16: ^{1}H NMR Spectrum of L08 (500 MHz, $CDCl_3$)....................S20

Figure S17: ^{13}C NMR Spectrum of L08 (125 MHz, $CDCl_3$) S21

Figure S18: HR-ESI-MS Spectrum of L08....................S21

Figure S19: ^{1}H NMR Spectrum of L09 (500 MHz, $CDCl_3$)....................S22

Figure S20: ^{13}C NMR Spectrum of L09 (125 MHz, $CDCl_3$) S22

Figure S21: HR-ESI-MS Spectrum of L09....................S23

Figure S22: ^{1}H NMR Spectrum of L10 (500 MHz, $CDCl_3$)....................S23

Figure S23: ^{13}C NMR Spectrum of L10 (125 MHz, $CDCl_3$) S24

Figure S24: HR-ESI-MS Spectrum of L10....................S24

Figure S25: ^{1}H NMR (400MHz, $CDCl_3$) of intermediate 2. ....S25

Figure S26: ^{1}H NMR (400MHz, $CDCl_3$) of intermediate 3. ....S25

Figure S27: ^{13}C NMR (400MHz, $CDCl_3$) of intermediate 3. ....S26

Figure S28: ^{1}H NMR (400MHz, $CDCl_3$) of intermediate 4. The signals that were not picked belong to the starting material leftover. S26

Figure S29: ^{1}H NMR (400MHz, $CDCl_3$) of intermediate 5. Signals at 3.92 and 1.13 ppm are attributed to isopropanol used in the column..S27

Figure S30: ^{13}C NMR (400MHz, $CDCl_3$) of intermediate 5. Signals at 64.13 and 25.20 ppm are attributed to isopropanol used in the column..S27

Figure S31: ^{1}H NMR (400MHz, $CDCl_3$) of L11. ....S28

Figure S32: ^{13}C NMR (400MHz, $CDCl_3$) of L11. ....S28

Figure S33: HR-ESI-MS Spectrum of L11..S29

Figure S34: Panel containing the bioavailability radars of compounds L03–L11, obtained in in silico tests. S30

Figure S35: Stability study of the L10 ligand, by UV-Vis spectroscopy, in (**a**) DMSO, (**b**) water, (**c**) PBS and (**d**) citrate buffer S31

Figure S36: Stability study of the L09 ligand, by UV-Vis spectroscopy, in (**a**) DMSO, (**b**) water and (**c**) PBS..S32

Figure S37: Improvement in the control of neuroinflammation and reduced Aβ plaques caused by treatment with the compounds. (**a**) Representative immunofluorescence images of the Control, STZ, STZ+L9, STZ+L10, and STZ+L11 groups in the DG region of the hippocampus for Aβ (red), GFAP (green), and DAPI (blue). (**b**), (**c**) Signal intensity was quantified using graphs, where the mean fluorescence intensity is represented by bars, with their standard error bars shown. S33

Figure S38: Improvement in the control of neuroinflammation and reduced Aβ plaques caused by treatment with the compounds. (**a**) Representative immunofluorescence images of the Control, STZ, STZ+L9, STZ+L10, and STZ+L11 groups in the CA1 region of the hippocampus for Aβ (red), GFAP (green), and DAPI (blue). (**b**), (**c**) Signal intensity was quantified using graphs, where the mean fluorescence intensity is represented by bars, with their standard error bars shown. S34

Figure S39: Improvement in the control of neuroinflammation and reduced Aβ plaques caused by treatment with the compounds. (**a**) Representative immunofluorescence images of the Control, STZ, STZ+L9, STZ+L10, and STZ+L11 groups in the CA3 region of the hippocampus for Aβ (red), GFAP (green), and DAPI (blue). S35

Figure S40: Regulation of ATP7B expression levels resulting from treatment with the new ligands. Representative immunofluorescence images of the Control, STZ, STZ+L9, STZ+L10, and STZ+L11 groups in the (**a**) DG and (**b**) CA1 regions of the hippocampus for ATP7B (pink) and DAPI (blue). The signal intensity in the (**c**) DG and (**d**) CA1 regions was quantified using graphs, where the mean fluorescence intensity is represented by bars, with their standard error bars shown. S36

Figure S41: Regulation of ATP7B expression levels resulting from treatment with the new ligands. Representative immunofluorescence images of the Control, STZ, STZ+L9, STZ+L10, and STZ+L11 groups in the CA3 region of the hippocampus for ATP7B (pink) and DAPI (blue). S37

Figure S42: Negative control for GFAP and Aβ analysis by immunofluorescence, being (**a**) control, (**b**) STZ, (**c**) STZ + L9, (**d**) STZ + L10 and (**e**) STZ + L11 ....S38

Figure S43: Negative control for ATP7B analysis by immunofluorescence, being (**a**) control, (**b**) STZ, (**c**) STZ + L9, (**d**) STZ + L10 and (**e**) STZ + L11 ....S39

Synthesis and General Procedures

All reactions were carried out under standard, opened to air conditions. Ethyl acetate (EtOAc), and hexane were locally purchased and purified by fractional distillation. Methanol (MeOH), ethanol (EtOH), dimethyl sulfoxide (DMSO), methylene chloride (DCM), toluene and isopropanol (IPA) were purchased from Merk and used without prior purification. 3,5-dichloroaniline, acetaldehyde, *N*,*N*-dimethylacetamide, 2-picolylamine, vanillin, ethyl vanillin, cinnamaldehyde, benzaldehyde, 2,2′-(ethylenedioxy)diethylamine, 2,2′-Oxydiethylamine and 2-(pyridin-2-yl)isopropyl amine were purchased from Merck-Sigma-Aldrich and used without further purification. Thin layer chromatography (TLC) was done using Silica Gel 60 F254 plates (Merck, 0.25 mm thick) and analyzed by UV lamp (254 nm) and stains as noted. Flash chromatography was performed in glass columns using Silica Gel 60 (Aldrich, 40-63 µm). ^{1}H and ^{13}C NMR spectra were recorded, respectively, at 500 and 125 MHz on a Varian INOVA 500 spectrometer (Palo Alto, CA, USA) or at 400 and 101 MHz on a Bruker DPX-400 Avance spectrometer V using $CDCl_3$ (Sigma-Aldrich) as solvent and TMS as internal standard. Chemical shifts are reported in parts per million (ppm). Data are reported as follows: chemical shift, multiplicity (s = singlet, bs = broad singlet, d = doublet, dd = doublet of doublets, ddd = doublet of doublet of doublets, t = triplet, td = triplet of doublets, q = quartet, sept = septet, m = multiplet), coupling constant (if applicable) and integration. For mass spectrometry analysis, a 1 mL sample of the compounds (1 mg/1 mL) solubilized in MeOH was analyzed via high-resolution (5000) UPLC-MS analysis by direct infusion. The UPLC-MS analysis was performed using a UHPLC (Acquity Waters) coupled to a mass spectrometer (Q-ToF microTM Micromass, Waters) using electrospray ionization (ESI). The mobile phase was composed of H_2O:MeOH 1:9 with 0.1% formic acid (FA)

Experimental procedures and spectral data

Synthesis of imines L03-L10

Synthesis of *N,N′*-bis(4-hydroxy-3-methoxyphenyl)-2,2'-(ethylenedioxy)diethylamine (L03)

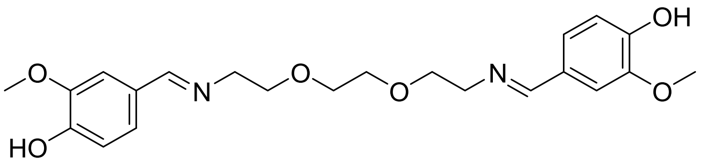

In a 50 mL round bottom flask was added a solution of vanillin (457 mg, 3 mmol) in 15 mL of EtOH. Then, 2,2′-(ethylenedioxy)diethylamine (220 μL, 1.5 mmol) was added dropwise and the reaction was continued with stirring at room temperature overnight. Evaporation of the solvent on a rotary evaporator yielded the product L03 as a yellow microcrystalline solid without further purification (691 mg, 55% yield).

^{1}H NMR (500 MHz, $CDCl_3$): δ 8.12 (s, 2H), 7.39 (s, 2H), 7.01 (d, *J* = 8.0 Hz, 2H), 6.84 (d, *J* = 8.0 Hz, 2H), 3.75 (bs, 8H), 3.72 (bs, 6H), 3.59 (s, 4H). ^{13}C NMR ($CDCl_3$, 125 MHz): δ 163.1, 149.3, 147.6, 127.9, 124.2, 114.5, 108.7, 70.7, 70.4, 60.4, 55.7. HR-ESI-MS (*m/z*) calcd. for $C_{22}H_{29}N_2O_6$ $[M+H]^+$ calcd.: 417.2026; found: 417.2049.

Synthesis of *N,N′*-bis(4-hydroxy-3-ethoxyphenyl)-2,2'-(ethylenedioxy)diethylamine (L04)

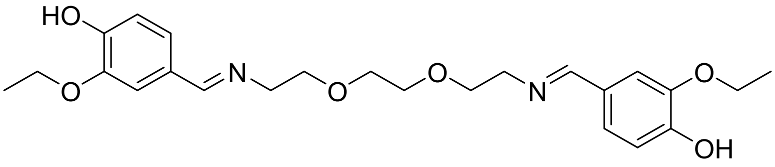

In a 50 mL round bottom flask was added a solution of ethyl vanillin (500 mg, 3 mmol) in 15 mL of EtOH. Then, 2,2′-(ethylenedioxy)diethylamine (220 μL, 1.5 mmol) was added dropwise and the reaction was continued with stirring at room temperature overnight. Evaporation of the solvent on a rotary evaporator yielded the product L04 as a yellow microcrystalline solid without further purification (858 mg, 64% yield).

^{1}H NMR (500 MHz, $CDCl_3$): δ 8.13 (s, 2H), 7.38 (d, *J* = 1.5 Hz, 2H), 7.05 (d, *J* = 8.0 Hz, 2H), 6.88 (d, *J* = 8.0, 2H), 4.07 (q, *J* = 7.0 Hz, 4H), 3.73 (bs, 8H), 3.61 (bs, 4), 1.37 (t, *J* = 7.0, 6H). ^{13}C NMR ($CDCl_3$, 125 MHz): δ 162.8, 148.7, 146.4, 128.5, 123.9, 70.8, 70.4, 64.4, 60.6, 14.7. HR-ESI-MS (*m/z*) calcd. for $C_{24}H_{33}N_2O_6$ $[M+H]^+$ calcd.: 445.2399; found: 445.2356.

Synthesis of *N,N′*-bis(3-phenyl-2-propen-1-ylidene)-2,2'-(ethylenedioxy)diethylamine (L05)

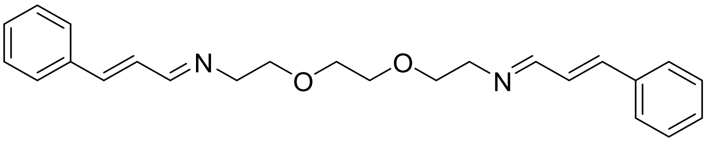

In a 50 mL round bottom flask was added a solution of cinnamaldehyde (378 μL, 3 mmol) in 15 mL of EtOH. Then, 2,2′-(ethylenedioxy)diethylamine (220 μL, 1.5 mmol) was added dropwise and the reaction was continued with stirring at room temperature overnight. Evaporation of the solvent on a rotary evaporator yielded the product L05 as a light-yellow oil without further purification (493 mg, 44% yield).

^{1}H NMR (500 MHz, $CDCl_3$): δ 8.02 (d, *J* = 7.0 Hz, 2H), 7.45 (d, *J* = 8.0 Hz, 4H), 7.36 – 7.28 (m, 8H), 6.90 (d, *J* = 8.0 Hz, 2H), 3.74 (t, *J* = 5.0 Hz, 4H), 3.69 (t, *J* = 5.0 Hz, 4H), 3.64 (bs, 4H). ^{13}C NMR ($CDCl_3$, 125 MHz): δ 164.3, 141.8, 135.7, 129.1, 128.8, 128.1, 70.87, 70.48, 60.85. HR-ESI-MS (*m/z*) calcd. for $C_{24}H_{29}N_2O_2$ $[M+H]^+$ calcd.: 377.2229; found: 377.2273.

Synthesis of *N,N′*-bis(1-phenyl-1-methyl-1-ylidene)-2,2'-(ethylenedioxy)diethylamine (L06)

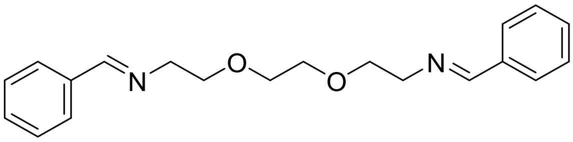

In a 50 mL round bottom flask was added a solution of benzaldehyde (306 μL, 3 mmol) in 15 mL of EtOH. Then, 2,2′-(ethylenedioxy)diethylamine (220 μL, 1.5 mmol) was added dropwise and the reaction was continued with stirring at room temperature overnight. Evaporation of the solvent on a rotary evaporator yielded the product L06 as a light-yellow oil without further purification (450 mg, 46% yield).

^{1}H NMR (500 MHz, $CDCl_3$): δ 8.25 (s, 2H), 7.70 (dd, *J* = 2.0 and 8.0 MHz, 4H), 7.38 – 7.35 (m, 6H), 3.74 (bs, 8H), 3.61 (s, 4H). ^{13}C NMR ($CDCl_3$, 125 MHZ): δ 162.8, 136.1, 130.6, 128.5, 128.2, 70.7, 70.5, 61.0. HR-ESI-MS (*m/z*) calcd. for $C_{20}H_{25}N_2O_2$ $[M+H]^+$ calcd.: 325.1916; found: 325.1907.

Synthesis of *N,N′*-bis(4-hydroxy-3-methoxyphenyl)-2,2'-oxydiethylamine (L07)

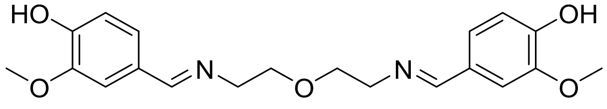

In a 25 mL round bottom flask was added a solution of vanillin (73 mg, 0.48 mmol) in 4 mL of EtOH. Then, 2,2′-Oxydiethylamine (25 μL, 0.24 mmol) was added dropwise and the

reaction was continued with stirring at room temperature overnight. Evaporation of the solvent on a rotary evaporator yielded the product L07 as a brown oil without further purification (160 mg, 90% yield).

^{1}H NMR (500 MHz, $CDCl_3$): δ 8.10 (s, 2H), 7.34 (d, *J* = 2.0 Hz, 2H), 6.95 (dd, *J* = 2.0 and 8.0 Hz, 2H), 6.83 (d, *J* = 8.0 Hz, 2H), 3.81 – 3.72 (m, 8H). ^{13}C NMR ($CDCl_3$, 125 MHz): δ 162.9, 148.8, 147.2, 128.5, 124.2, 114.2, 108.4, 70.4, 60.5, 55.8. HR-ESI-MS (*m/z*) calcd. for $C_{20}H_{25}N_2O_5$ $[M+H]^+$ calcd.: 373.1763; found: 373.1744

Synthesis of *N,N'*-bis(4-hydroxy-3-ethoxyphenyl)-2,2'-oxydiethylamine (L08)

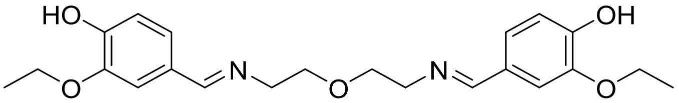

In a 25 mL round bottom flask was added a solution of ethyl vanillin (80 mg, 0.48 mmol) in 4 mL of EtOH. Then, 2,2′-oxydiethylamine (25 μL, 0.24 mmol) was added dropwise and the reaction was continued with stirring at room temperature overnight. The product L08 was obtained as a white solid, which was filtered and washed with ice-cold EtOH (71 mg, 37% yield).

^{1}H NMR (500 MHz, $CDCl_3$): δ 8.12 (s, 2H), 7.33 (d, *J* = 2.00 Hz, 2H), 7.00 (dd, *J* = 2.00 and 8.00 Hz, 2H), 6.88 (d, *J* = 8.00 Hz, 2H), 4.10 (q, *J* = 7.00 Hz, 4H), 3.79 – 3.72 (m, 8H), 1.43 (t, *J* = 7.00 Hz, 6H). ^{13}C NMR ($CDCl_3$, 125 MHz): δ 162.2, 148.4, 146.2, 128.8, 123.8, 113.9, 109.1, 70.6, 60.8. HR-ESI-MS (*m/z*) calcd. for $C_{22}H_{29}N_2O_5$ $[M+H]^+$ calcd.: 401.2076; found: 401.2029

Synthesis of *N*-hydroxy-3-methoxyphenyl-2-(pyridin-2-yl)isopropyl amine (L09)

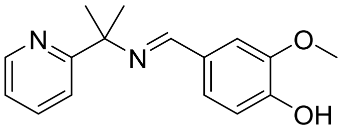

In a 25 mL round bottom flask fitted with a Dean-Stark apparatus were added vanillin (55 mg, 0.36 mmol) and 2-(pyridin-2-yl)isopropyl amine (52.5 μL, 0.38 mmol) in 2 mL of toluene. The reaction was heated at reflux until no further water was collected in the trap. Then, evaporation of the solvent on a rotary evaporator yielded the product L09 as an orange-yellow oil without further purification (50 mg, 47% yield).

^{1}H NMR (500 MHz, $CDCl_3$): δ 8.58 (d, *J* = 5.0 Hz, 1H), 8.24 (s, 1H), 7.62 (td, *J* = 2.0 and 7.0 Hz, 1H), 7.53 (d, *J* = 8.0 Hz, 1H), 7.49 (d, *J* = 2.0 Hz, 1H), 7.13 – 7.01 (m, 2H), 6.88 (d, *J* = 8.0 Hz, 1H), 3.82 (s, 3H), 1.69 (s, 6H). ^{13}C NMR ($CDCl_3$, 125 MHz): δ 166.8, 157.5, 149.0, 148.5, 147.5, 136.4, 129.1, 123.8, 121.4, 120.9, 114.5, 108.8, 64.4, 55.8, 28.5. HR-ESI-MS (*m/z*) calcd. for $C_{16}H_{19}N_2O_2$ $[M+H]^+$ calcd.: 271.1447; found: 271.1445

Synthesis of *N*-4-hydroxy-3-ethoxyphenyl -2-(pyridin-2-yl)isopropyl amine (L10)

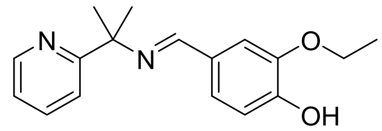

In a 25 mL round bottom flask fitted with a Dean-Stark apparatus were added ethyl vanillin (60 mg, 0.36 mmol) and 2-(pyridin-2-yl)isopropyl amine (52.5 μL, 0.38 mmol) in 2 mL of toluene. The reaction was heated at reflux until no further water was collected in the trap. Then, evaporation of the solvent on a rotary evaporator yielded the product L10 as an orange-yellow oil without further purification (57 mg, 53% yield).

^{1}H NMR (500 MHz, $CDCl_3$): δ 8.60 – 8.58 (m, 1H), 8.26 (s,1H), 7.63 (td, *J* = 2.0 and 7.0 Hz, 1H), 7.54 (dt, *J* = 2.0 and 8.0 Hz, 1H), 7.50 (d, *J* = 2.0 Hz, 1H), 7.18 (dd, *J* = 2.0 and 8.0 Hz, 1H), 7.14 – 7.11 (m, 1H), 6.94 (d, *J* = 8.0 Hz, 1H), 4.17 (q, *J* = 7.0 Hz, 2H), 1.68 (s, 6H), 1.43 (t, *J* = 7.0 Hz, 3H). ^{13}C NMR ($CDCl_3$, 125 MHz): δ 167.0, 157.1, 148.5, 148.5, 146.4, 136.2, 129.6, 123.5, 120.9, 114.2, 109.6, 64.5, 64.4, 28.5, 14.8. HR-ESI-MS (*m/z*) calcd. for $C_{17}H_{21}N_2O_2$ $[M+H]^+$ calcd.: 285.1603; found: 285.1487

Synthesis of quinoline L11

Synthesis of 5,7-dichloro-2-methylquinoline (intermediate 2)

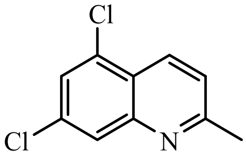

A 250 mL two-neck round-bottom flask was charged with a stir bar and a solution of 3,5-dichloroaniline (12.96 g, 80 mmol) in 60 mL of 12 M HCl. The flask was placed in an ice bath at 0 °C, with one neck attached to a condenser and the other sealed with a septum. A heart-shaped flask containing acetaldehyde (18 mL, 420 mmol) was also placed in the ice bath and sealed with a septum. The acetaldehyde was added dropwise to the 3,5-dichloroaniline solution via cannula under nitrogen pressure. After the complete addition of acetaldehyde, the reaction mixture was stirred for 15 min at 0 °C. The ice bath was then removed, and the temperature was gradually increased to 80 °C. The mixture was stirred at this temperature for 4 h. After completion, the reaction mixture was poured into ice-cold water and slowly neutralized with portions of aqueous NH_4OH. The resulting solution was extracted with DCM, and the organic phase was dried over Na_2SO_4, filtered, and concentrated under reduced pressure. The crude product was purified by silica gel flash column chromatography, eluting first with 20:1 hexane/EtOAc (to remove low-polarity byproducts) and then with 10:1

hexane/EtOAc (to isolate the product, which appeared as a reddish band on the column). Evaporation of the eluent yielded compound **2** as an orange solid (10.34 g, 61% yield).

^{1}H NMR (400 MHz, $CDCl_3$) δ 8.36 (d, *J* = 8.66 Hz, 1H), 7.92 (s, 1H), 7.52 (s, 1H), 7.35 (d, *J* = 8,63 Hz, 1H), 2.72 (s, 3H).

Synthesis of 5,7-dichloro-2-methyl-8-nitroquinoline (intermediate 3)

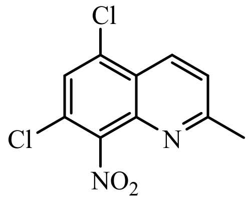

To a stirred solution of 5,7-dichloro-2-methylquinoline (**2**) (10.34 g, 48.76 mmol) in neat sulfuric acid (49 mL) in a 500 mL round-bottom flask equipped with stir bar, fuming nitric acid (16 mL) was added dropwise over 1 h while maintaining the reaction in an ice bath at 0 °C. After the addition was complete, the ice bath was removed, and the solution was stirred at ambient temperature for 3 h. The reaction mixture was then poured onto ice, and ammonium hydroxide was added dropwise until neutralization was achieved. The mixture was extracted with DCM, and the organic phase was dried over Na_2SO_4, filtered, and concentrated under reduced pressure. The crude product was purified by silica gel flash column chromatography, eluting first with 10:1 hexane/EtOAc (to remove low-polarity byproducts) and then with 7:3 hexane/EtOAc (to isolate the product). Evaporation of the solvent afforded intermediate **3** as a yellow solid (8.8 g, 70% yield).

^{1}H NMR (400 MHz, $CDCl_3$): δ 8.43 (d, *J* = 8.7 Hz, 1H), 7.50 (d, *J* = 8.7 Hz, 1H). ^{13}C NMR (101 MHz, $CDCl_3$): δ 163.73, 140.19, 133.69, 132.78, 125.93, 125.10, 124.68, 123.94, 25.54.

Synthesis of 5,7-dichloro-8-nitro-2-vinylquinoline (intermediate 4)

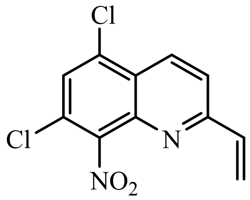

To a solution of 5,7-dichloro-2-methyl-8-nitroquinoline (**3**) (3.867 g, 15.04 mmol) in dimethylacetamide (DMA, 19 mL), charged in a 100 mL round-bottom flask equipped with a stir bar, $FeCl_3$ (73 mg, 0.45 mmol) and $K_2S_2O_8$ (8.1 g, 30 mmol) were added. The mixture was stirred at 110 °C overnight and then quenched with water. The reaction mixture was extracted with DCM, and the organic phase was dried over Na_2SO_4, filtered, and concentrated under reduced pressure. The crude product was purified by silica gel flash column chromatography,

eluting with 100% toluene. Evaporation of the eluent afforded compound **4** as a brown solid (1.961 g, 48% yield).

^{1}H NMR (400 MHz, $CDCl_3$): δ 8.51 (d, *J* = 8.9 Hz, 1H), 7.73 (d, *J* = 8.9 Hz, 1H), 7.65 (s, 1H), 6.97 (dd, *J* = 17.6, 10.8 Hz, 1H), 6.46 (d, *J* = 17.6 Hz, 1H), 5.79 (d, *J* = 10.8 Hz, 1H).

Synthesis of 2-(5,7-dichloro-8-nitroquinolin-2-yl)-*N*-(pyridin-2-ylmethyl)ethan-1-amine (intermediate 5)

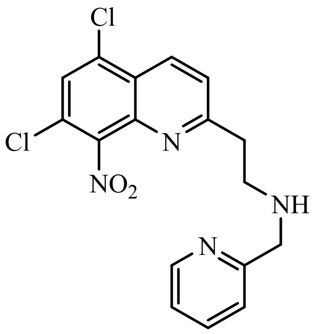

To a mixture of 5,7-dichloro-8-nitro-2-vinylquinoline (4) (1.962 g, 7.29 mmol) and K_2CO_3 (927 mg, 8.75 mmol) in 24 mL of 1,4-dioxane, charged in a 100 mL round-bottom flask equipped with a stir bar, 2-picolylamine (1.5 mL, 14.58 mmol) was added. The resulting mixture was stirred at room temperature overnight. After completion, the reaction was quenched with water and extracted with DCM. The organic phase was dried over Na_2SO_4, filtered, and concentrated under reduced pressure. The crude product was purified by silica gel flash column chromatography, eluting with 8:2:0.5 EtOAc/IPA/25% NH_4OH. Evaporation of the eluent afforded compound **5** as an orange solid (1.184 g, 43% yield).

^{1}H NMR (400 MHz, $CDCl_3$): δ 8.45 (d, *J* = 4.8 Hz, 1H), 8.36 (d, *J* = 8.7 Hz, 1H), 7.59 (m, 1H), 7.56 (s, 1H), 7.46 (d, *J* = 8.7 Hz, 1H), 7.25 (d, *J* = 7.7 Hz,1H), 7.08 (dd, *J* = 7.6, 4.8 Hz, 1H), 3.88 (s, 2H), 3.17 (t, *J* = 6.6 Hz, 2H), 3.08 (t, *J* = 6.2 Hz, 2H). ^{13}C NMR (101 MHz, $CDCl_3$): δ 165.4, 159.4, 149.2, 140.1, 136.6, 133.7, 132.9, 126.1, 125.2, 124.6, 124.2, 122.3, 122.0, 55.0, 47.5, 38.5.

Synthesis of 5,7-dichloro-2-(2-((pyridin-2-ylmethyl)amino)ethyl)quinolin-8-amine (L11)

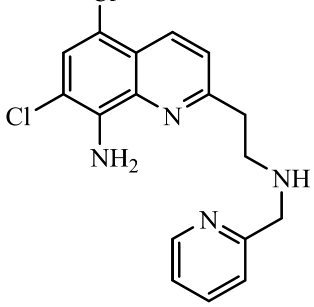

To a solution of 2-(5,7-dichloro-8-nitroquinolin-2-yl)-*N*-(pyridin-2-ylmethyl)ethan-1-amine (5) (1.184 g, 3.1 mmol) in 14 mL of EtOH, charged in a 50 mL round bottom flash equipped with stir bar, was added iron powder (526 mg, 9.4 mmol) and 2 mL of acetic acid (31 mmol), and the mixture was stirred under reflux for 4 h. The mixture was then poured into an aqueous saturated $NaHCO_3$ solution (20 mL) and extracted with DCM (3 x 10 mL). The combined organic layers were dried over Na_2SO_4, filtered, and concentrated under reduced pressure. The crude product was purified by silica gel flash column chromatography, eluting with 9:1 DCM/MeOH. Evaporation of the eluent afforded compound L11 a greenish oil (300 mg, 28% yield).

^{1}H NMR (400 MHz, $CDCl_3$): δ 8.56 (d, J = 5.0 Hz, 1H), 8.33 (d, J = 8.7 Hz, 1H), 7.66 (tt, J = 7.7, 1.4 Hz, 1H), 7.43 (s, 1H), 7.35 (d, J = 8.6 Hz, 1H), 7.33 – 7.25 (m, 1H), 7.25 – 7.16 (m, 1H), 5.59 (bs, 1H), 4.06 (s, 2H), 3.94 (bs, 2H), 3.37 – 3.27 (m, 2H), 3.28 – 3.19 (m, 2H). ^{13}C NMR (101 MHz, $CDCl_3$) δ 159.0, 157.9, 149.3, 139.8, 137.8, 136.8, 133.5, 126.8, 123.8, 122.7, 122.4, 117.5, 113.7, 54.3, 47.4, 37.1. HR-ESI-MS for $C_{17}H_{17}Cl_2N_4$ $[M+H]^+$ *m/z* 347.0822

^{1}H and ^{13}C NMR and HR-ESI-MS Spectra of compounds L03 – L10

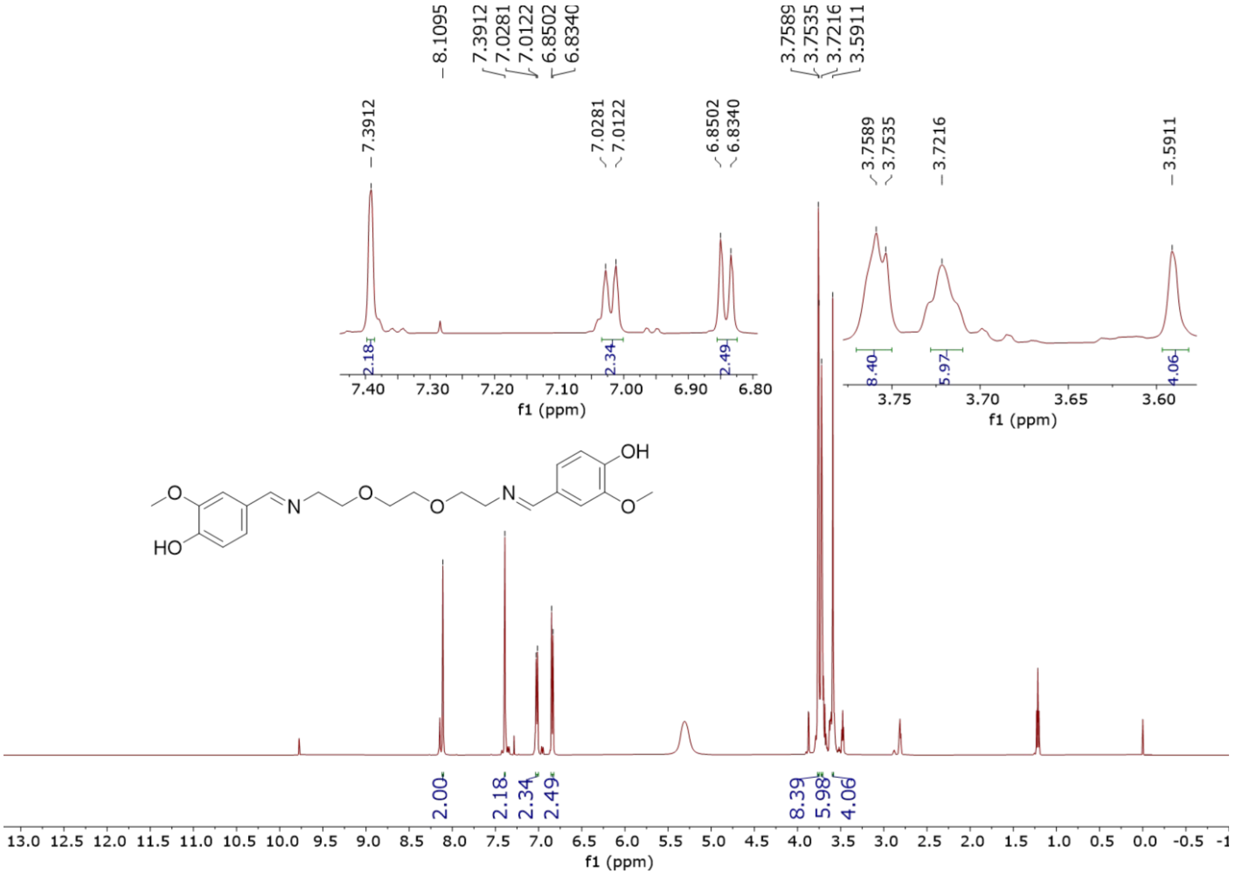

Figure S1: ^{1}H NMR Spectrum of L03 (500 MHz, $CDCl_3$)

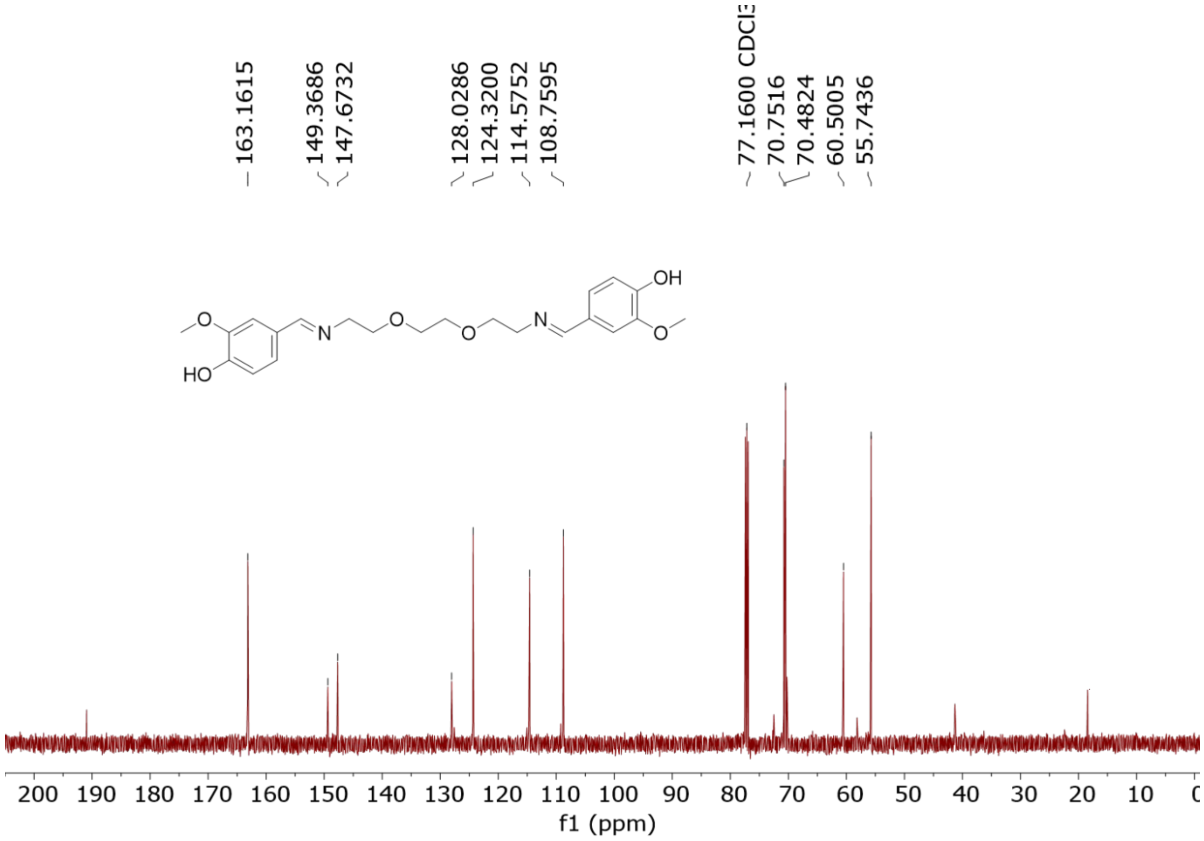

Figure S2: ^{13}C NMR Spectrum of L03 (125 MHz, $CDCl_3$)

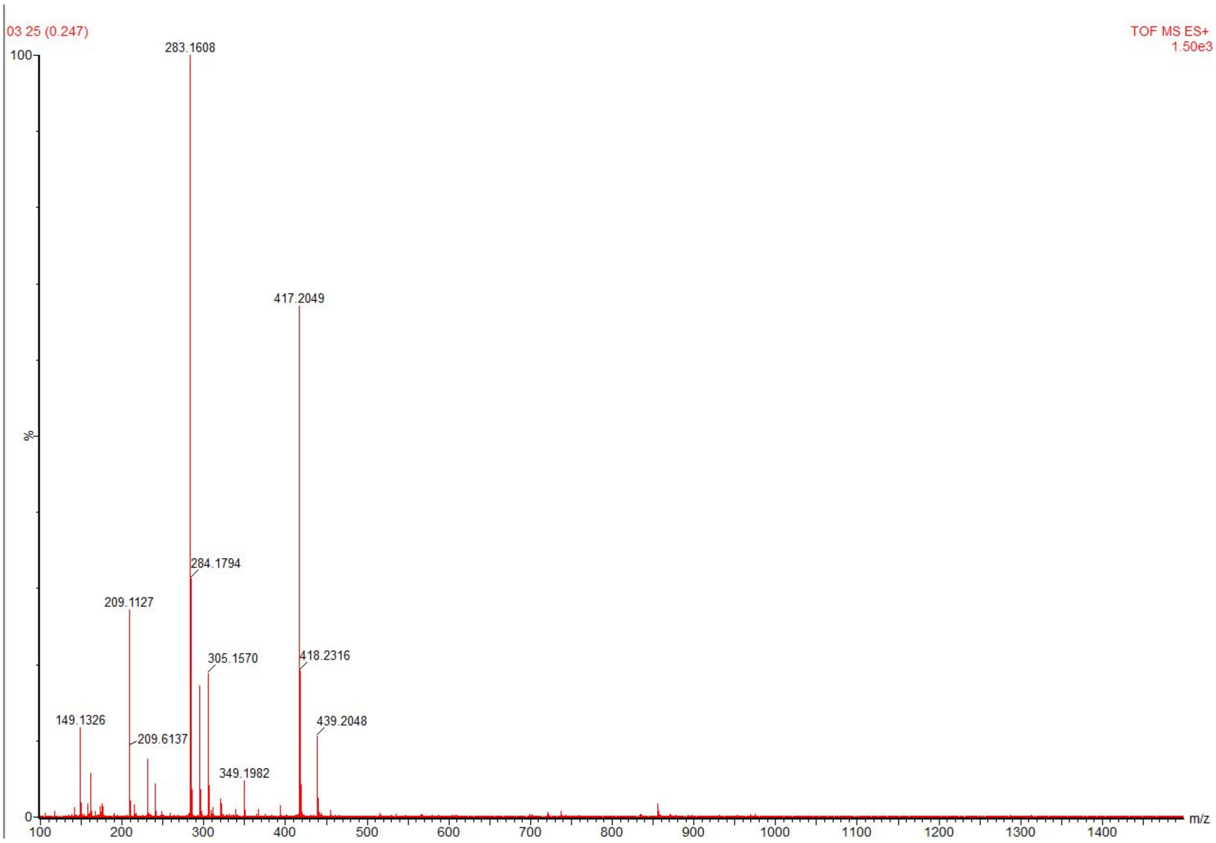

Figure S3: HR-ESI-MS Spectrum of L03

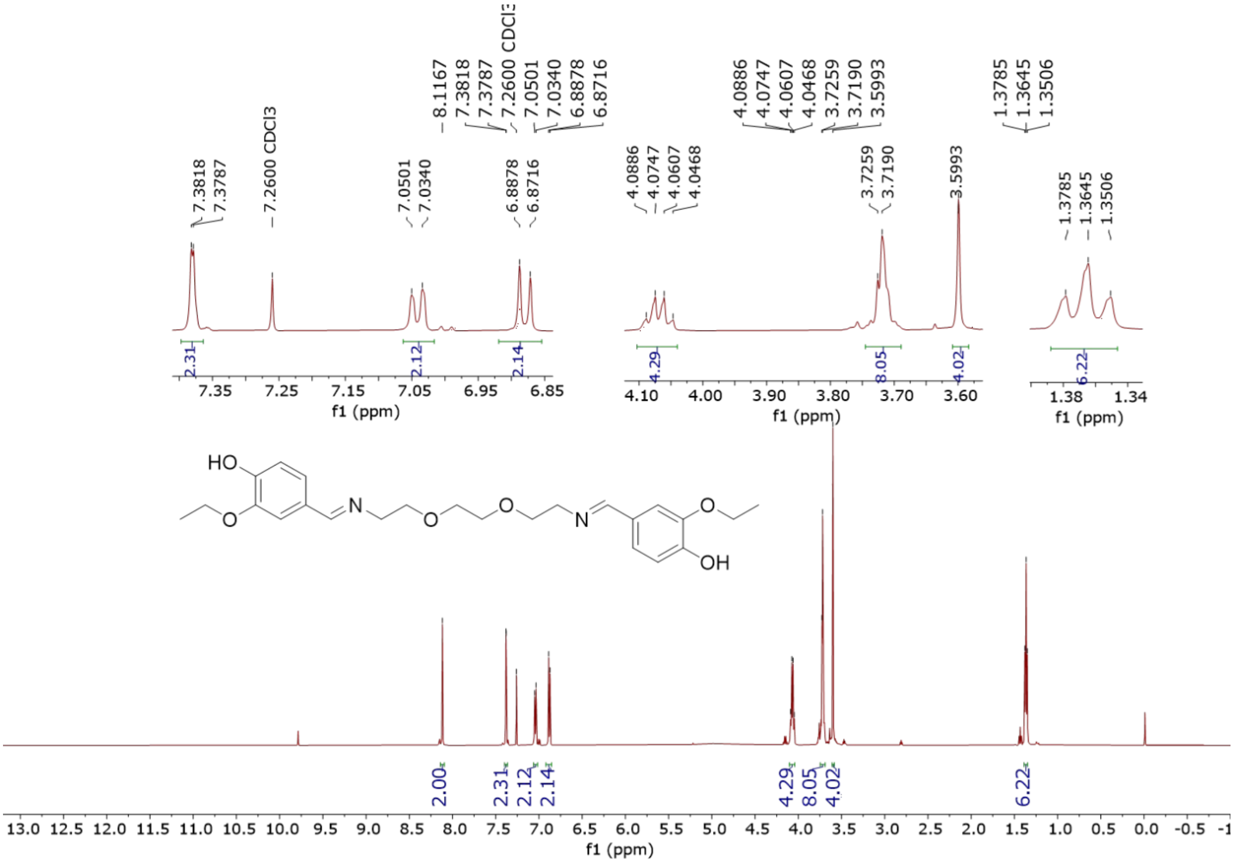

Figure S4: ^{1}H NMR Spectrum of L04 (500 MHz, $CDCl_3$)

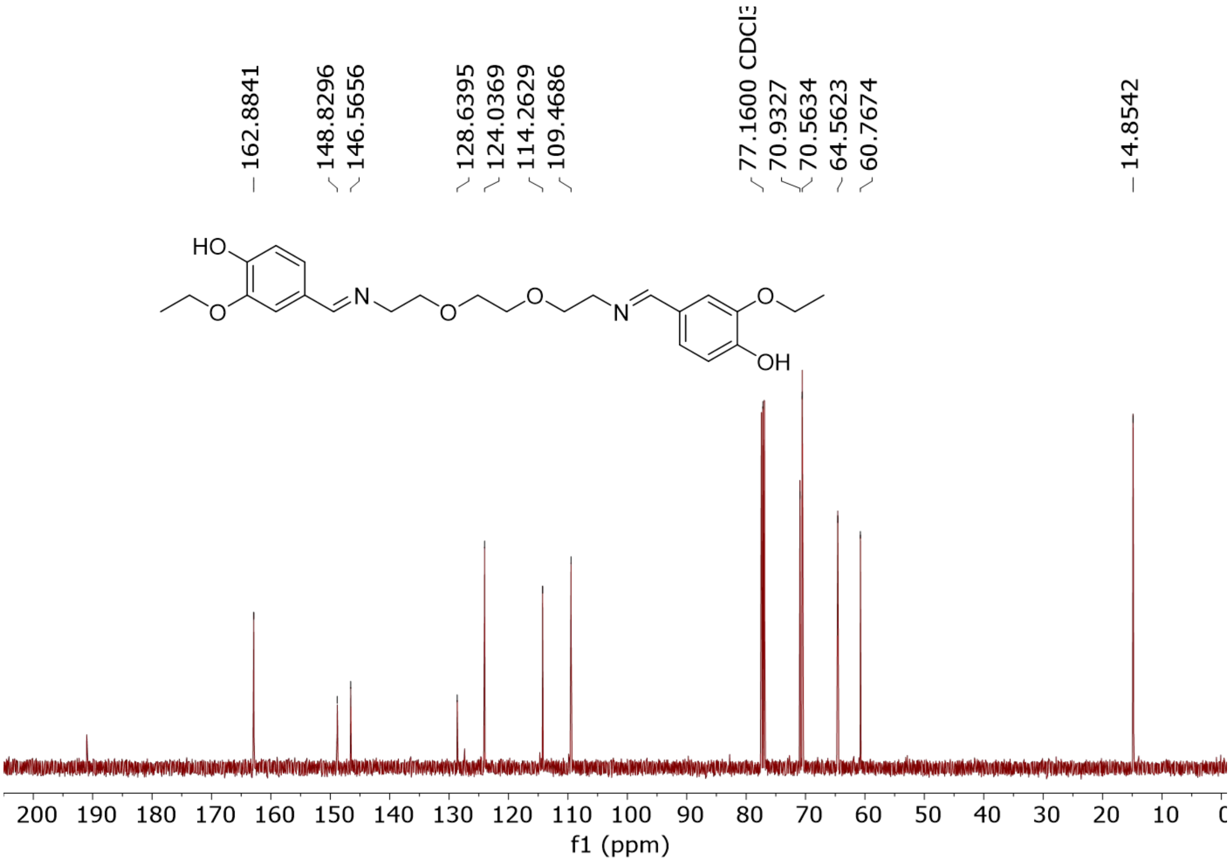

Figure S5: ^{13}C NMR Spectrum of L04 (125 MHz, $CDCl_3$)

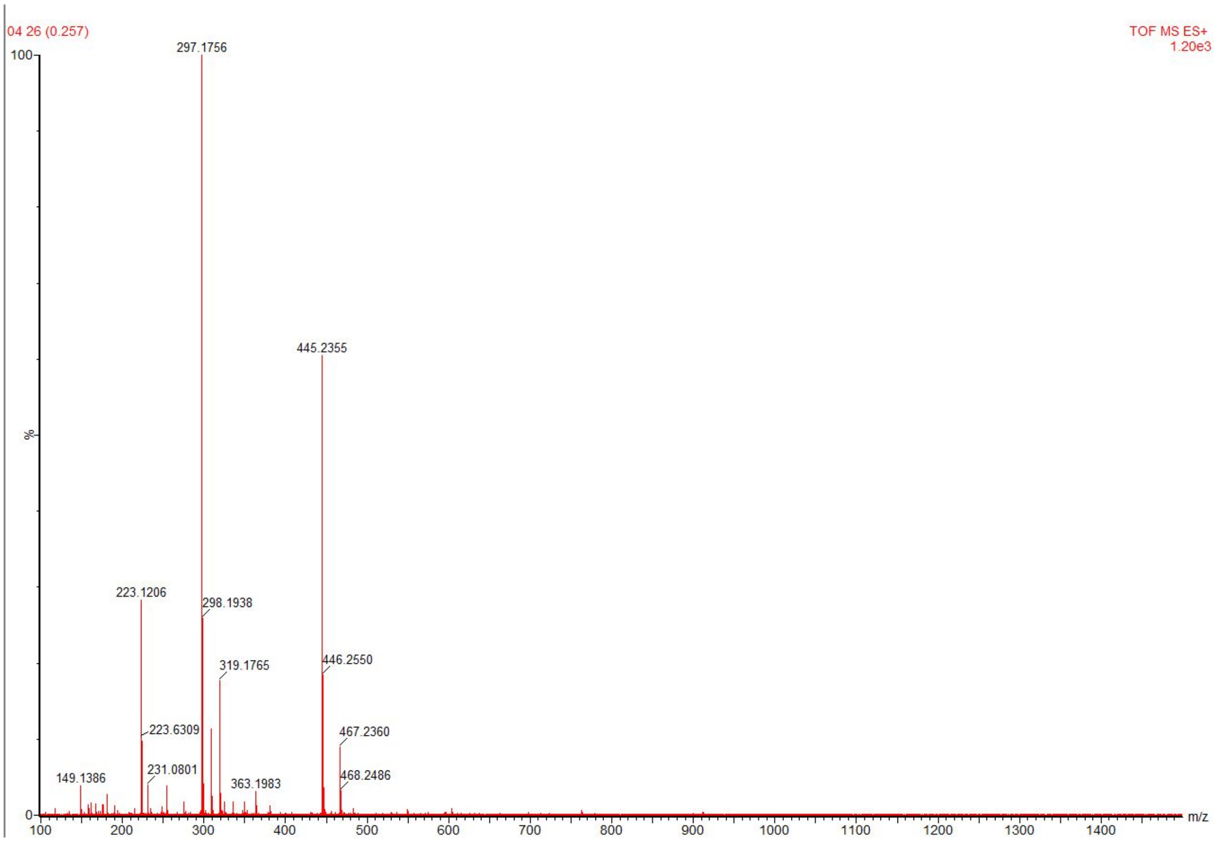

Figure S6: HR-ESI-MS Spectrum of L04

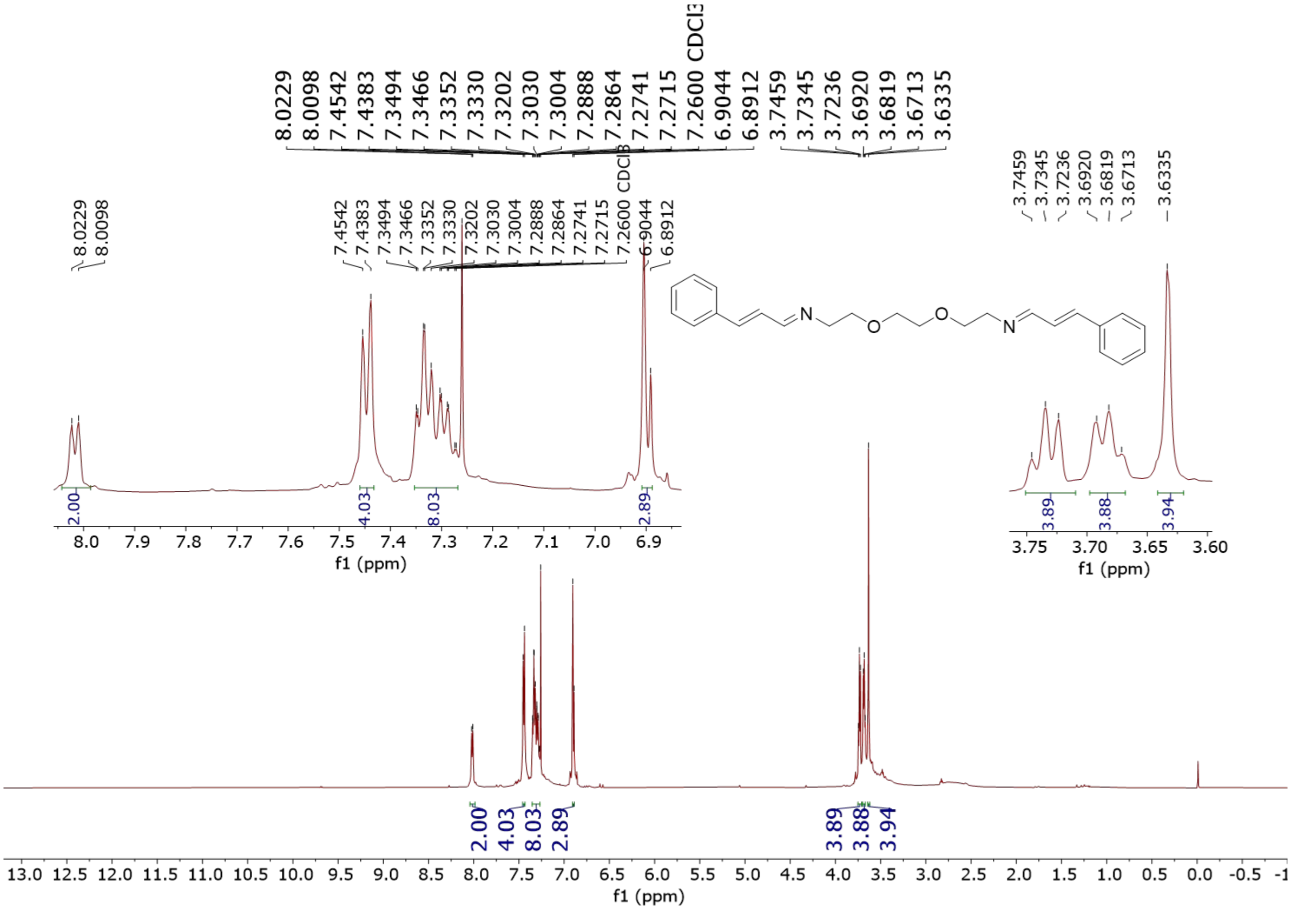

Figure S7: ^{1}H NMR Spectrum of L05 (500 MHz, $CDCl_3$)

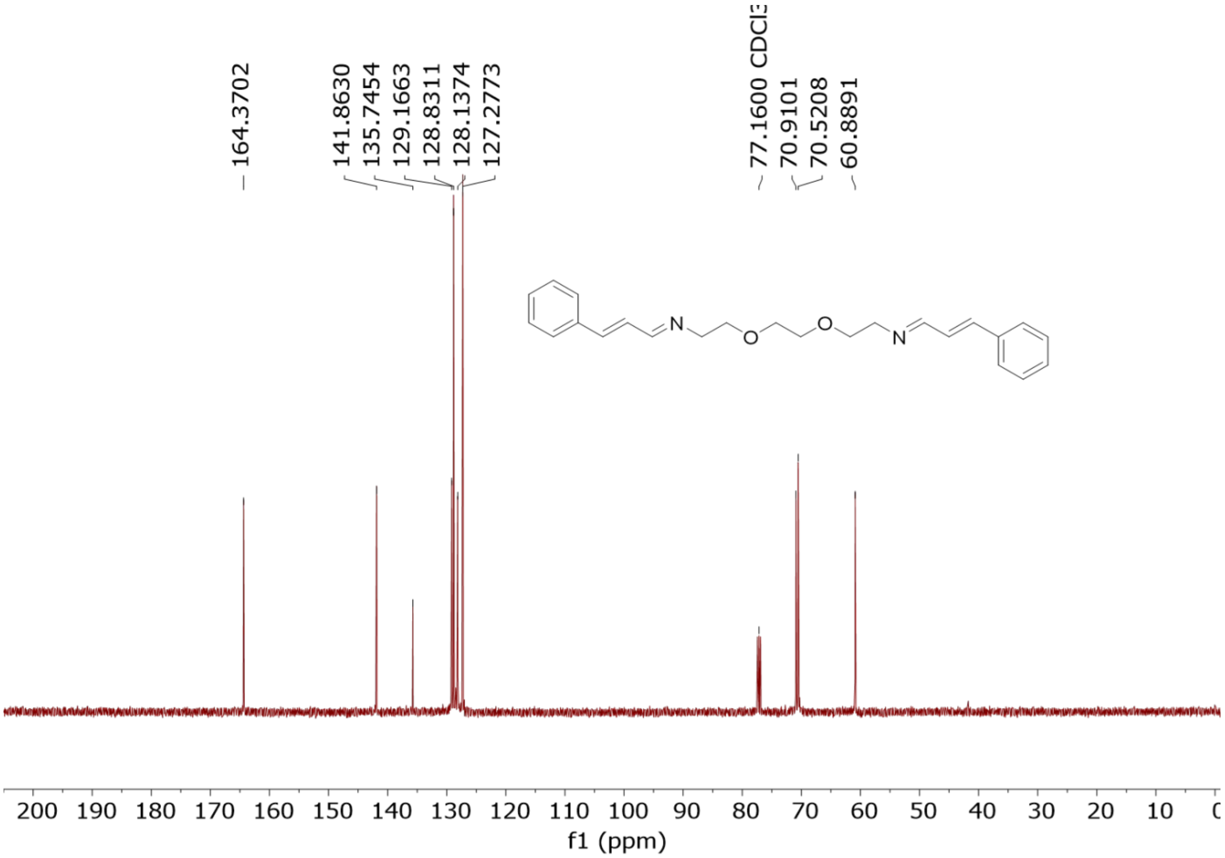

Figure S8: ^{13}C NMR Spectrum of L05 (125 MHz, $CDCl_3$)

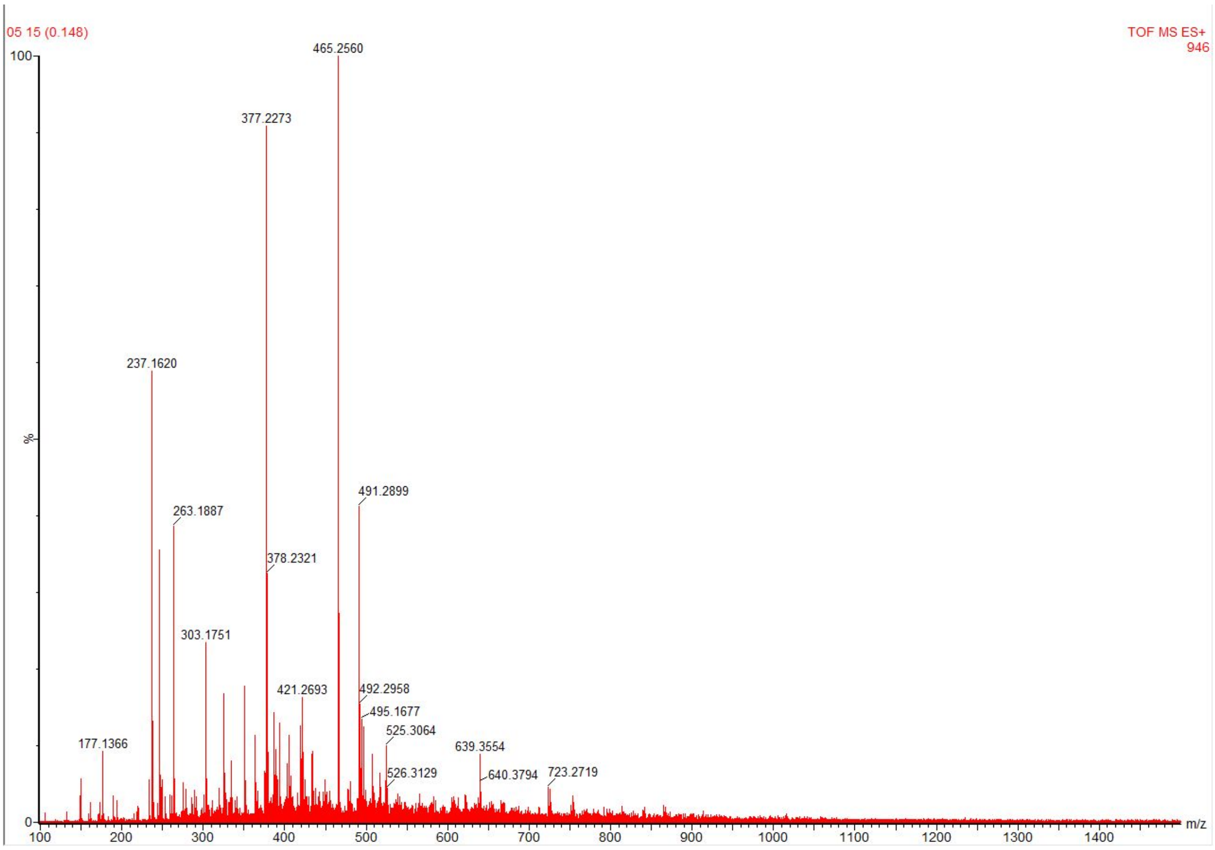

Figure S9: HR-ESI-MS Spectrum of L05

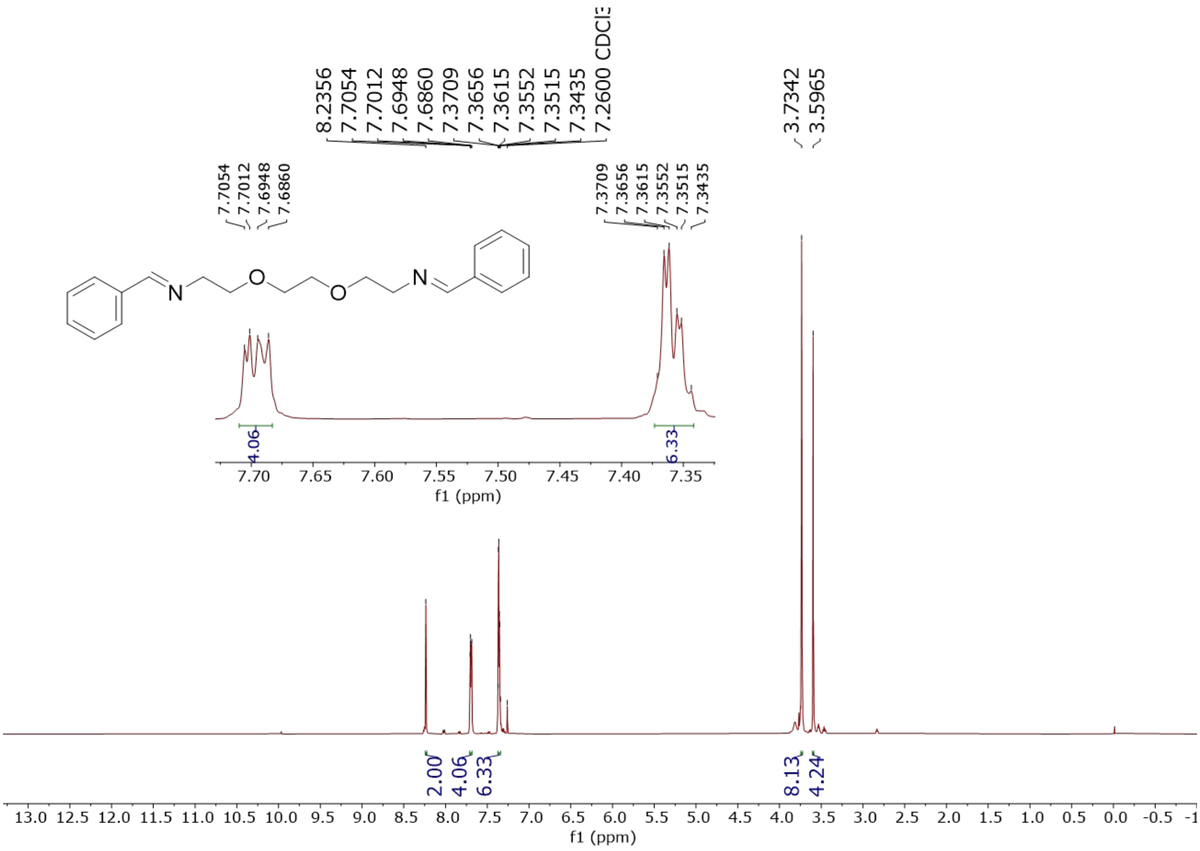

Figure S10: ^{1}H NMR Spectrum of L06 (500 MHz, $CDCl_3$)

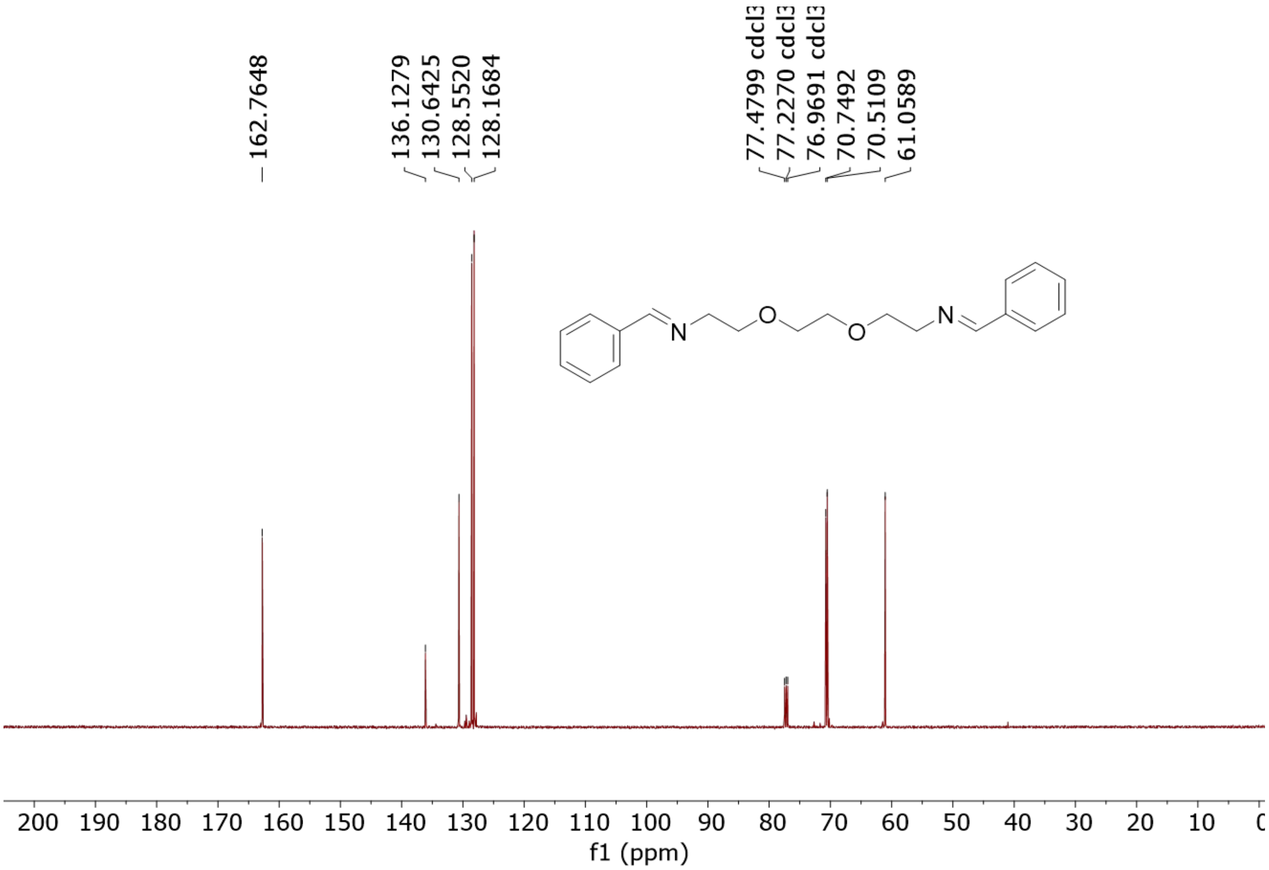

Figure S11: ^{13}C NMR Spectrum of L06 (125 MHz, $CDCl_3$)

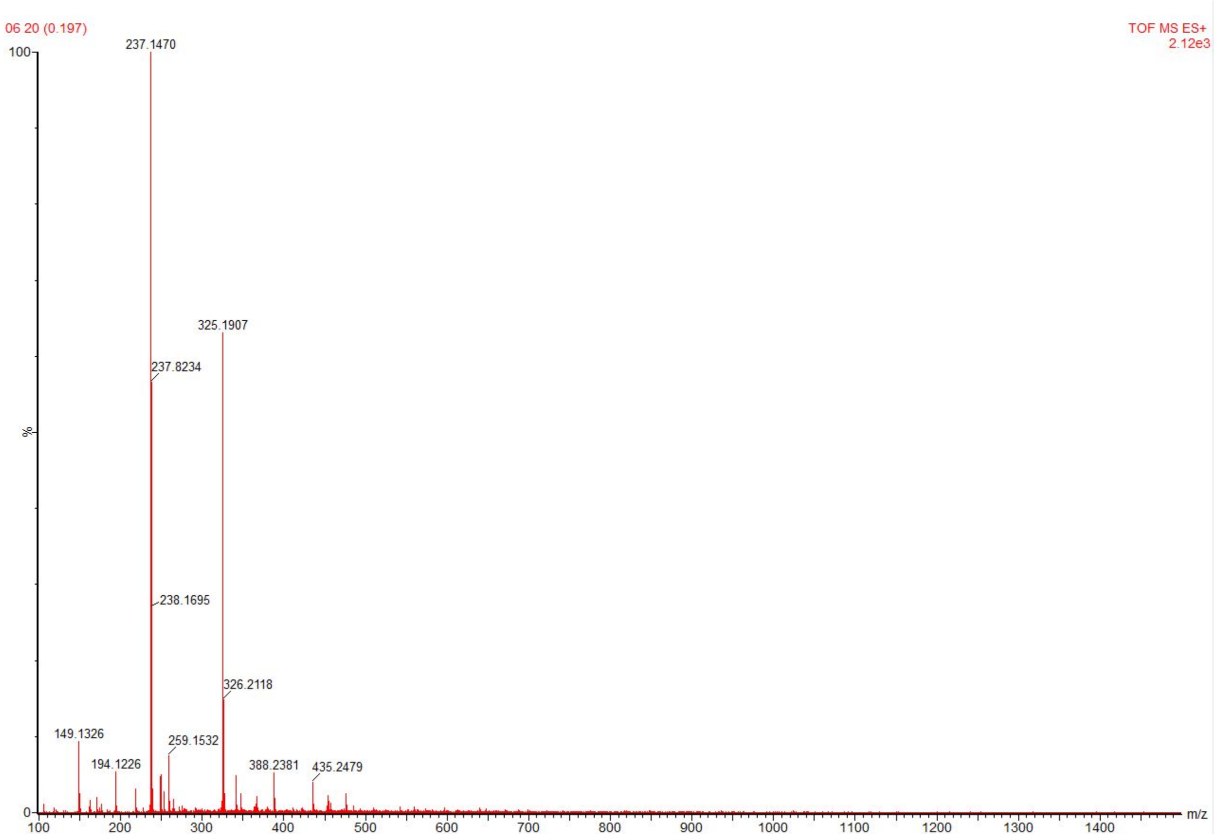

Figure S12: HR-ESI-MS Spectrum of L06

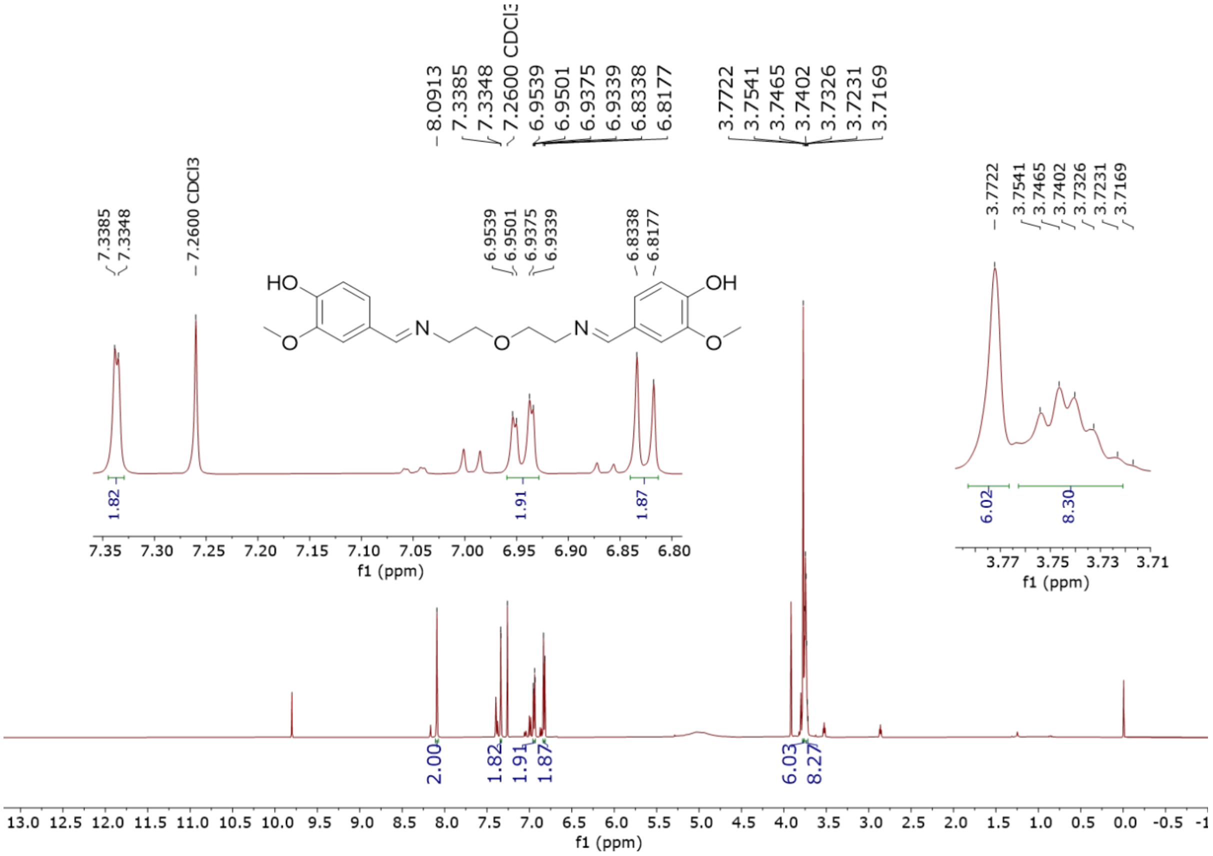

Figure S13: ^{1}H NMR Spectrum of L07 (500 MHz, $CDCl_3$)

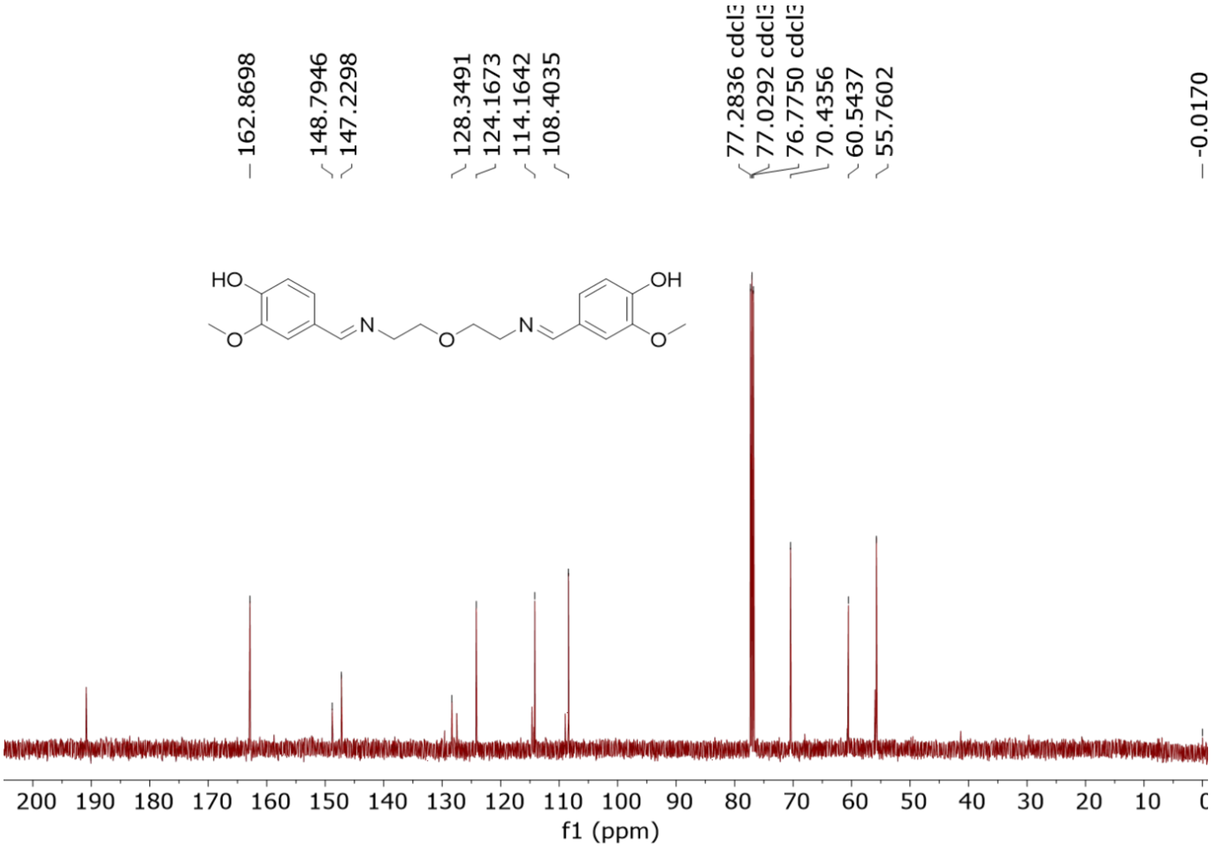

Figure S14: ^{13}C NMR Spectrum of L07 (125 MHz, $CDCl_3$)

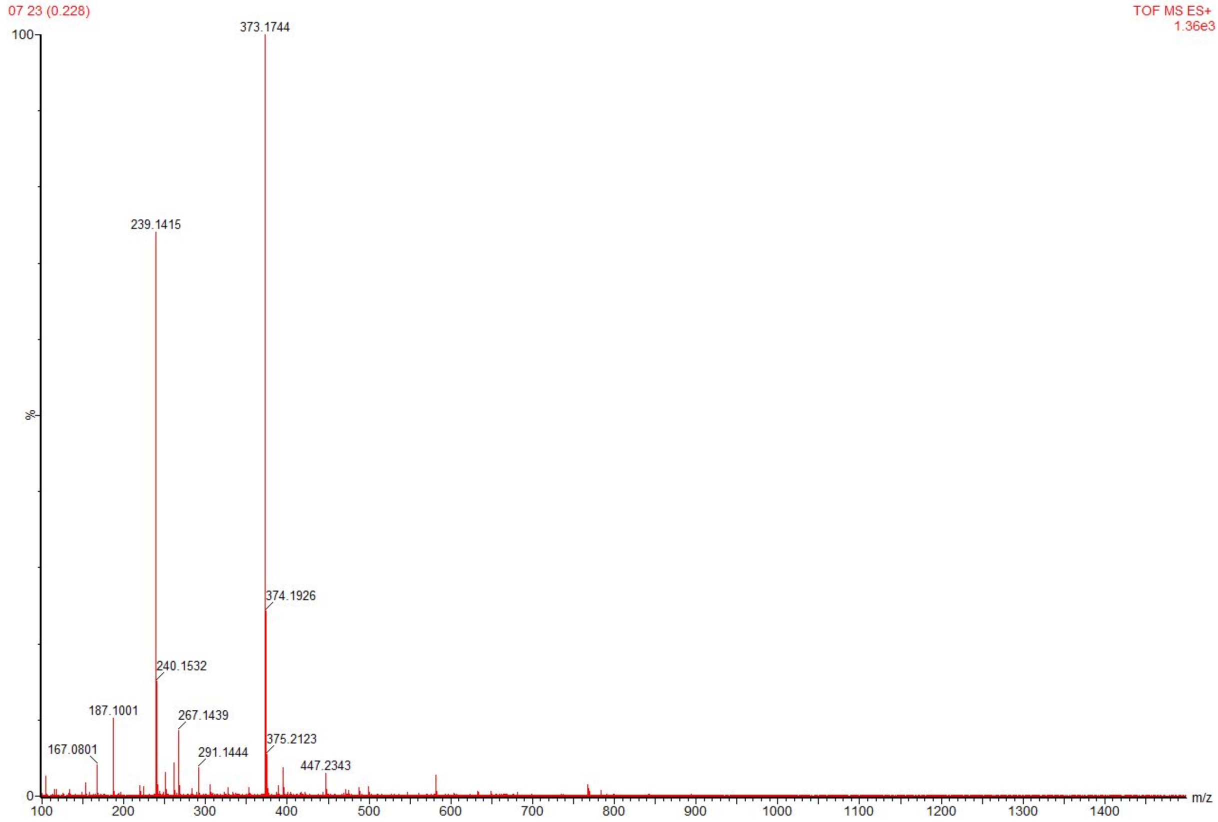

Figure S15: HR-ESI-MS Spectrum of L07

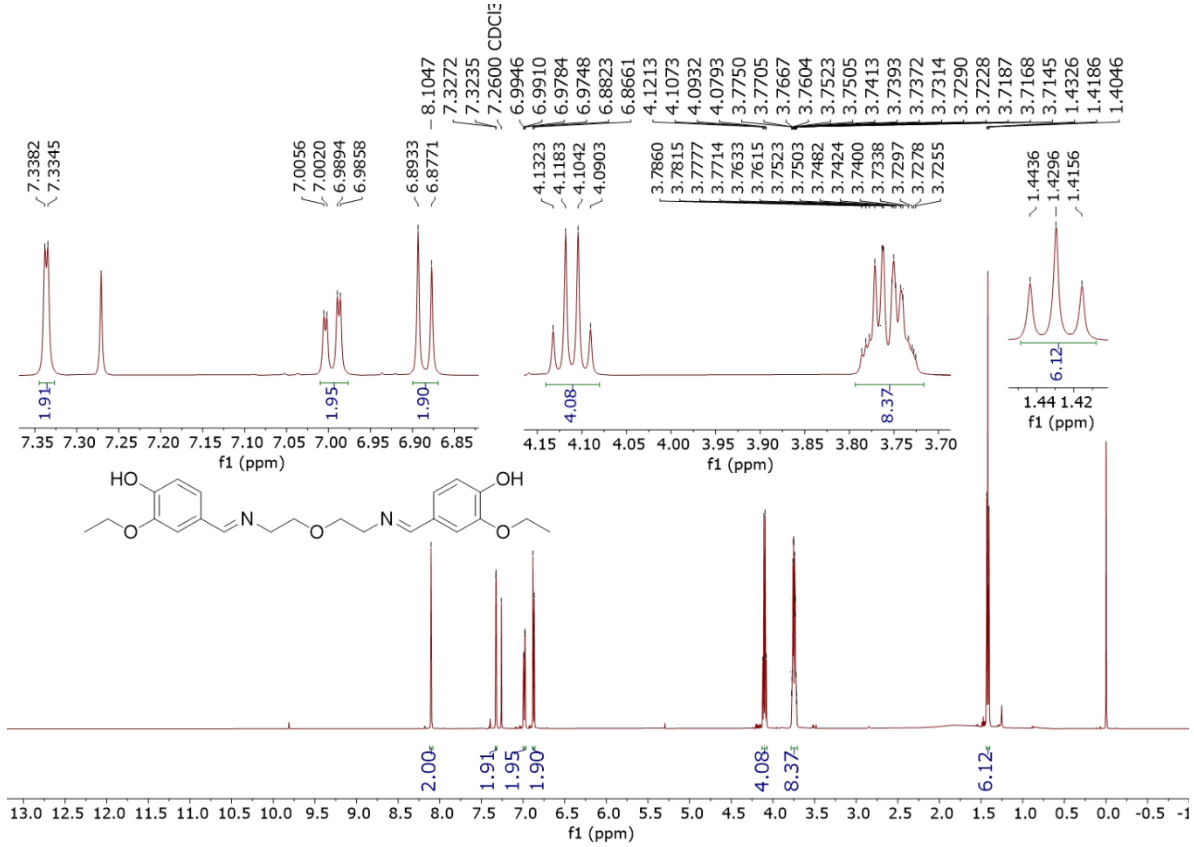

Figure S16: ^1H NMR Spectrum of L08 (500 MHz, $CDCl_3$)

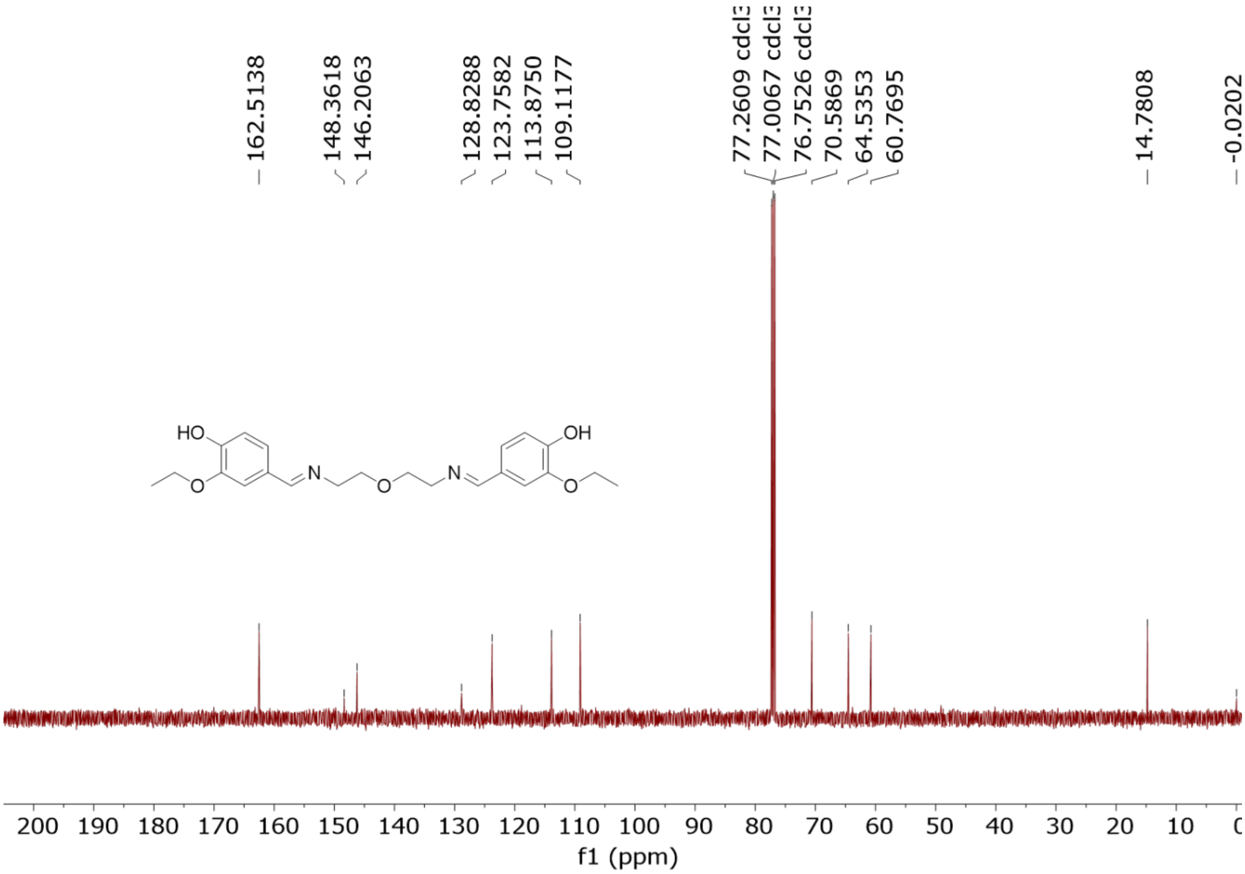

Figure S17: ^{13}C NMR Spectrum of L08 (125 MHz, $CDCl_3$)

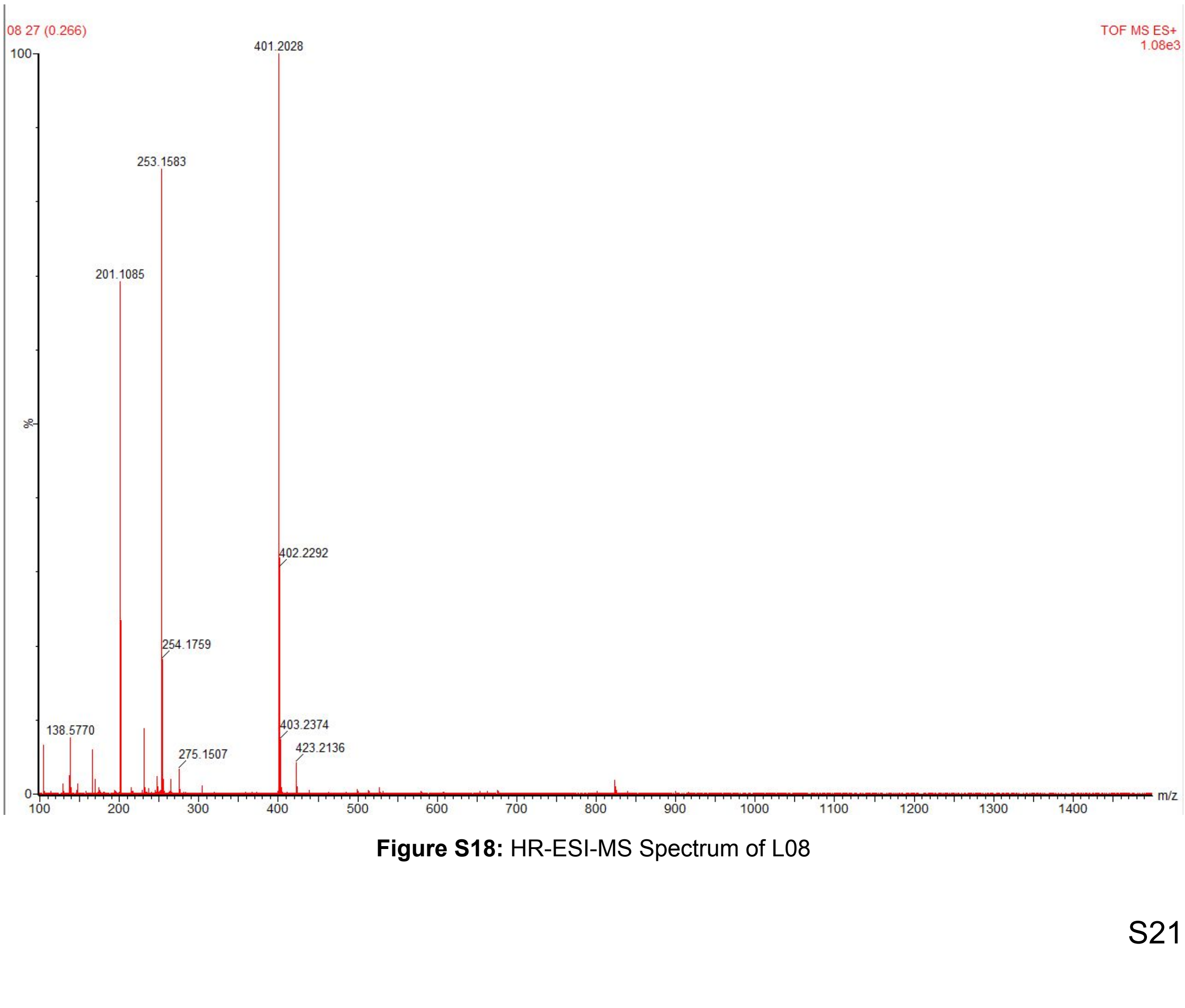

Figure S18: HR-ESI-MS Spectrum of L08

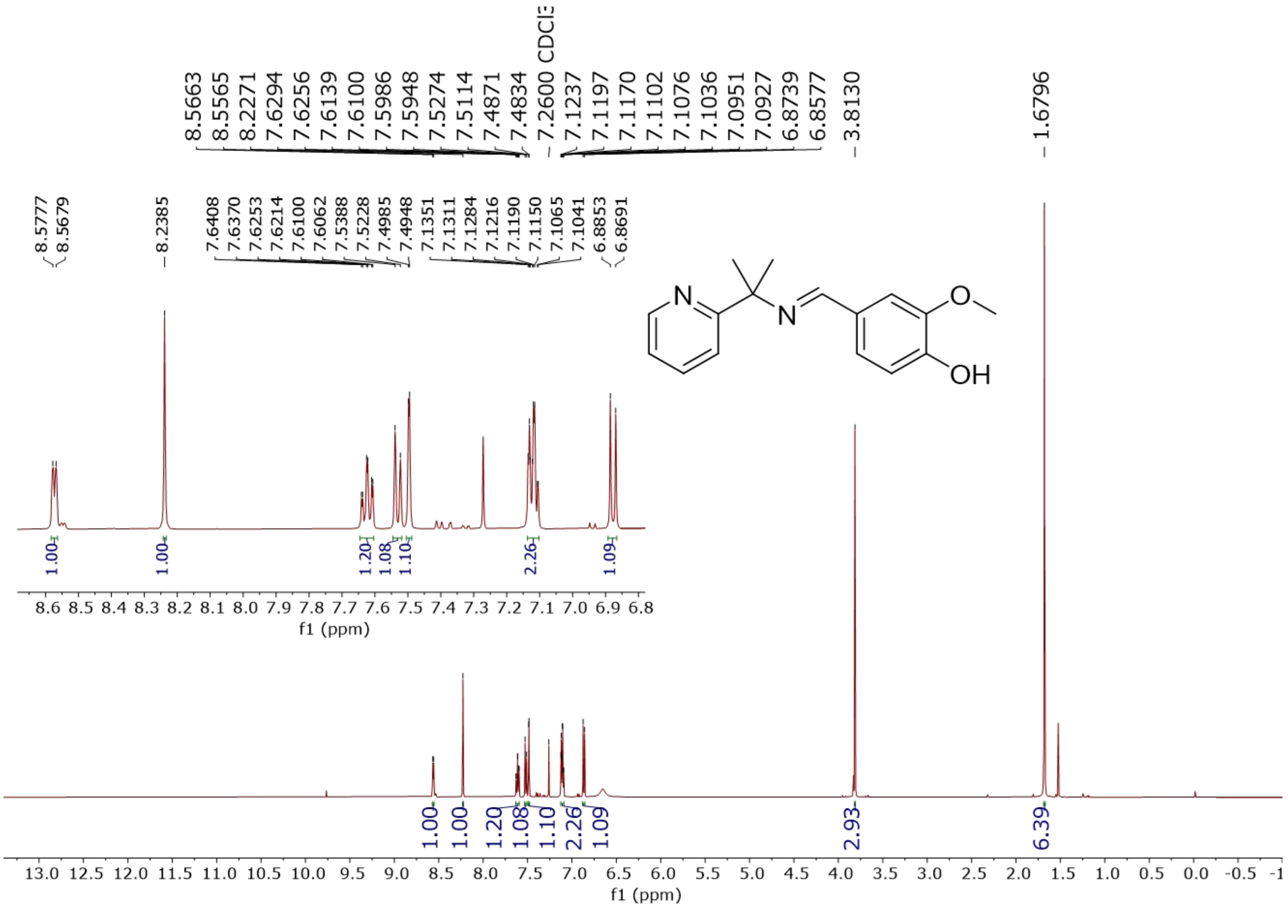

Figure S19: ^{1}H NMR Spectrum of L09 (500 MHz, $CDCl_3$)

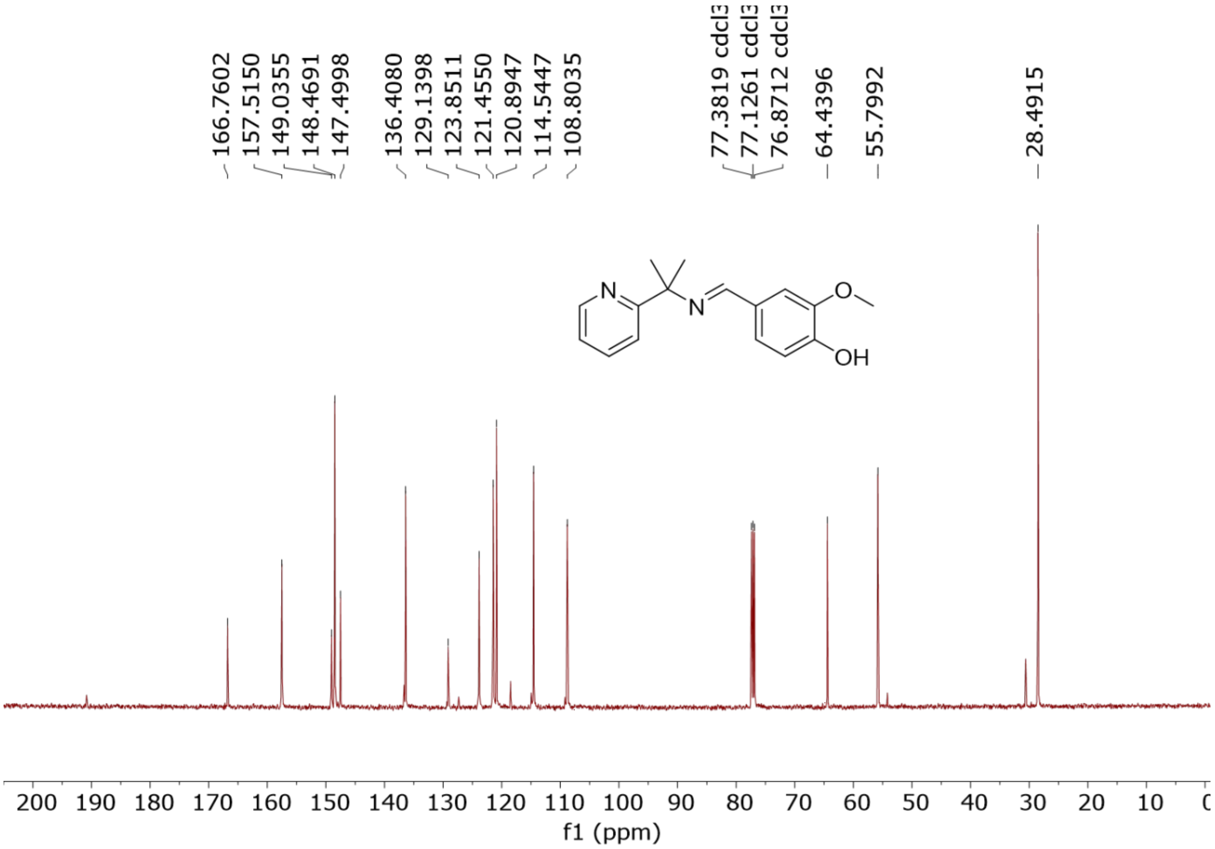

Figure S20: ^{13}C NMR Spectrum of L09 (125 MHz, $CDCl_3$)

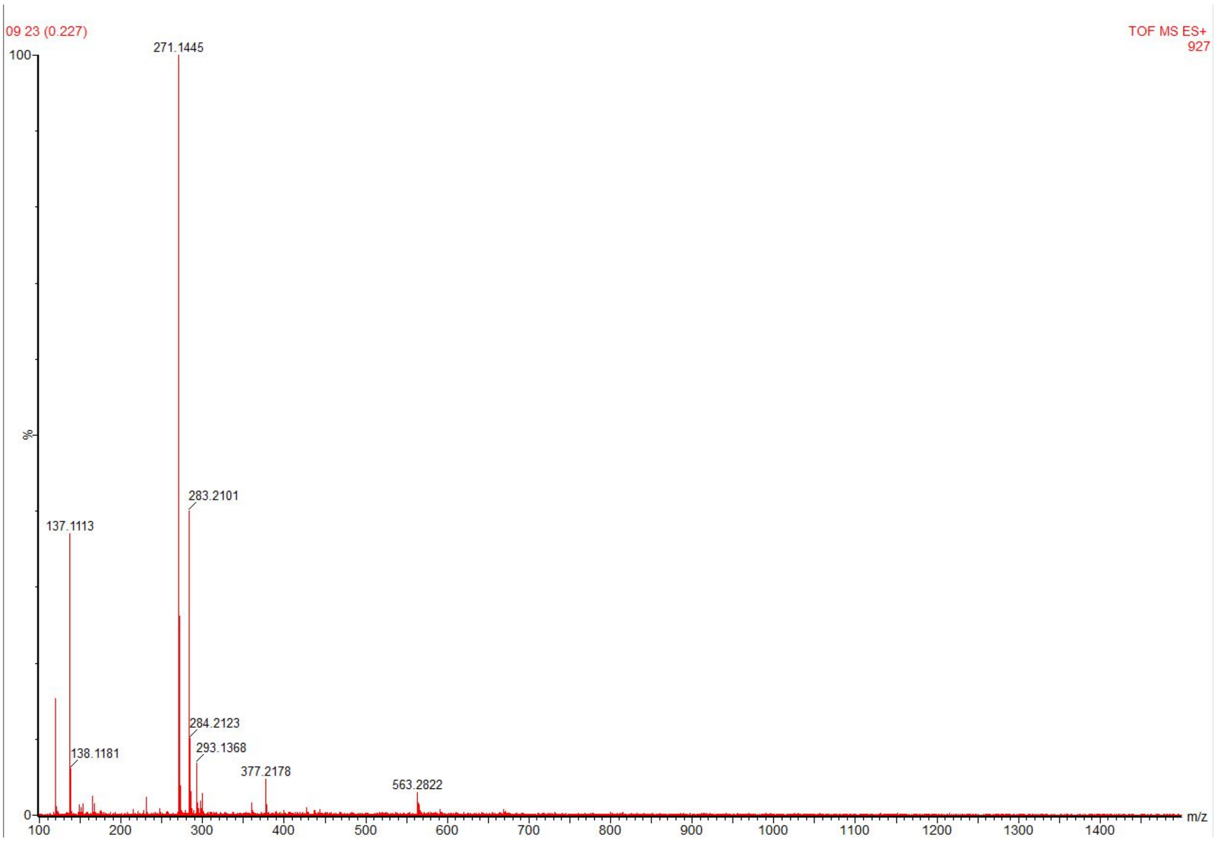

Figure S21: HR-ESI-MS Spectrum of L09

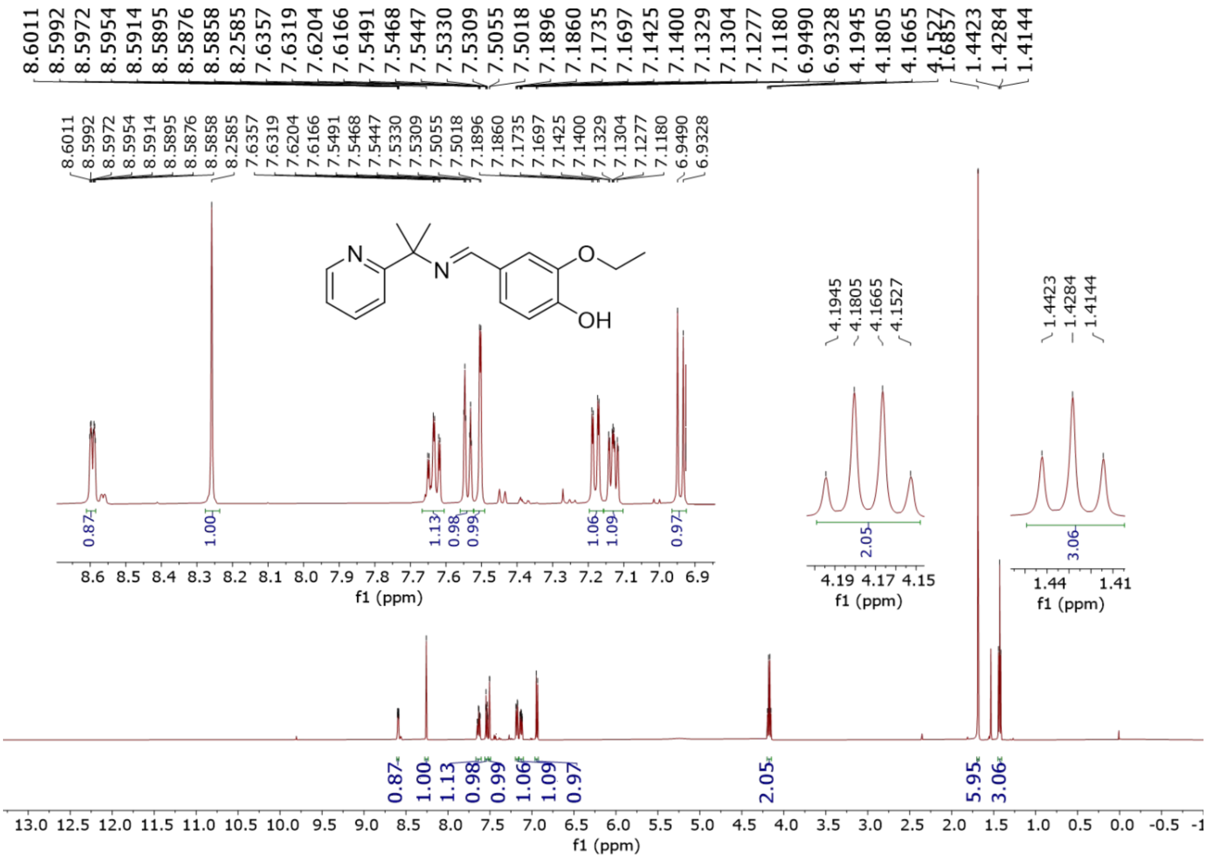

Figure S22: ^{1}H NMR Spectrum of L10 (500 MHz, $CDCl_3$)

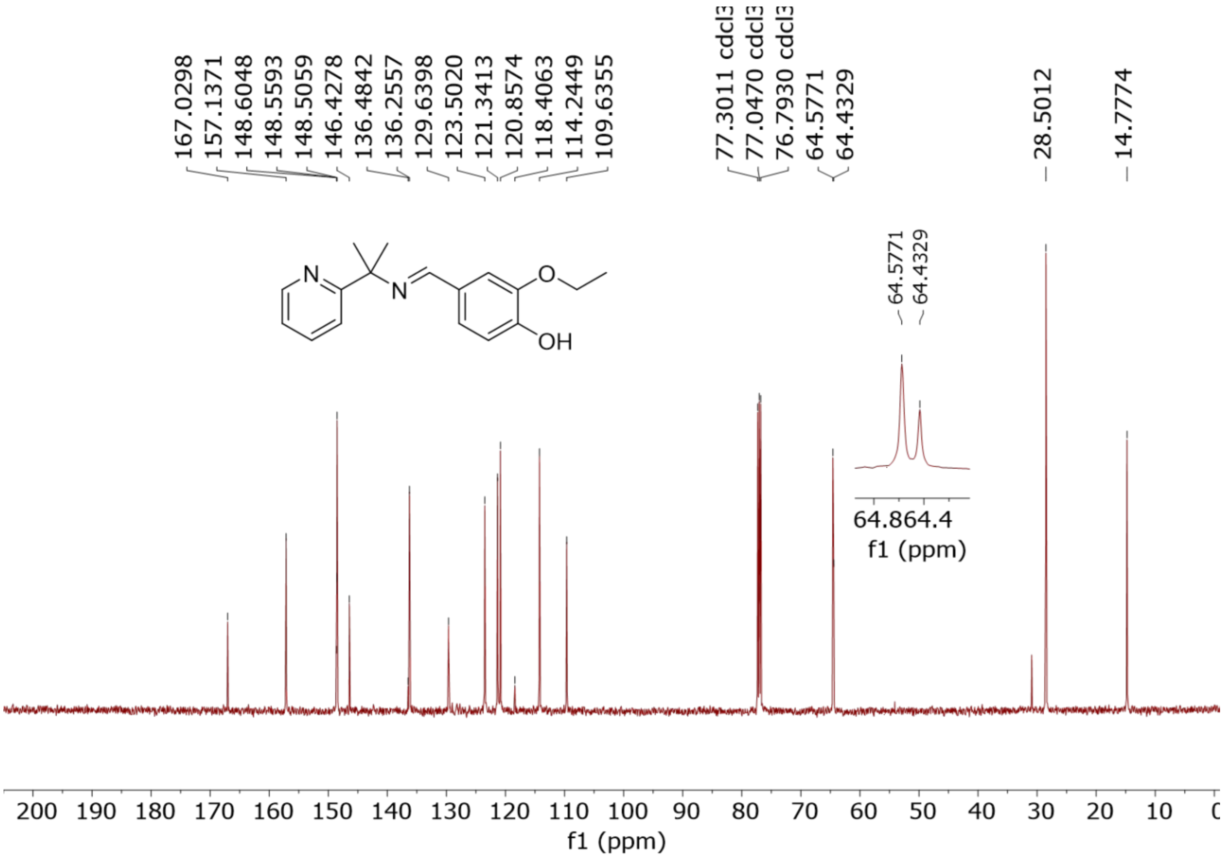

Figure S23: ^{13}C NMR Spectrum of L10 (125 MHz, $CDCl_3$)

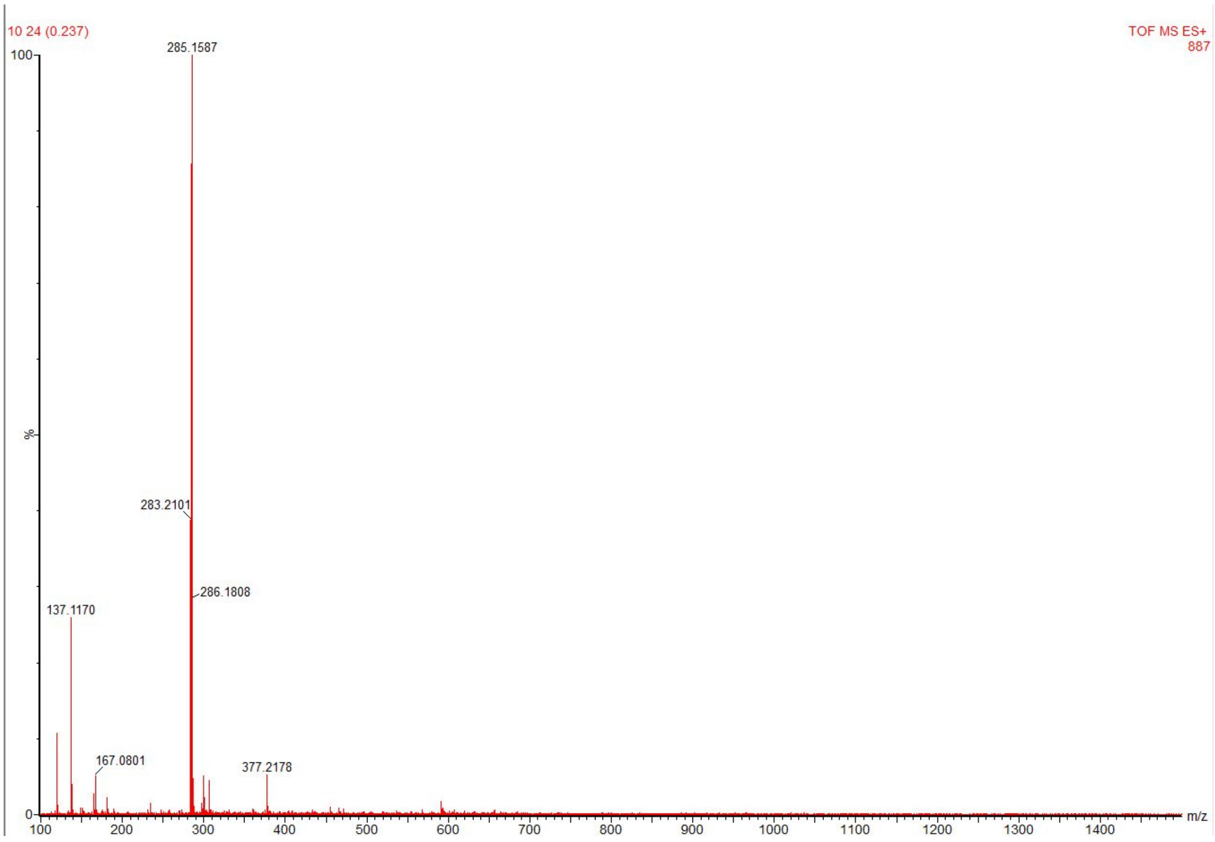

Figure S24: HR-ESI-MS Spectrum of L10

^{1}H and ^{13}C NMR Spectra of Intermediates 2 – 5 and Ligand L11

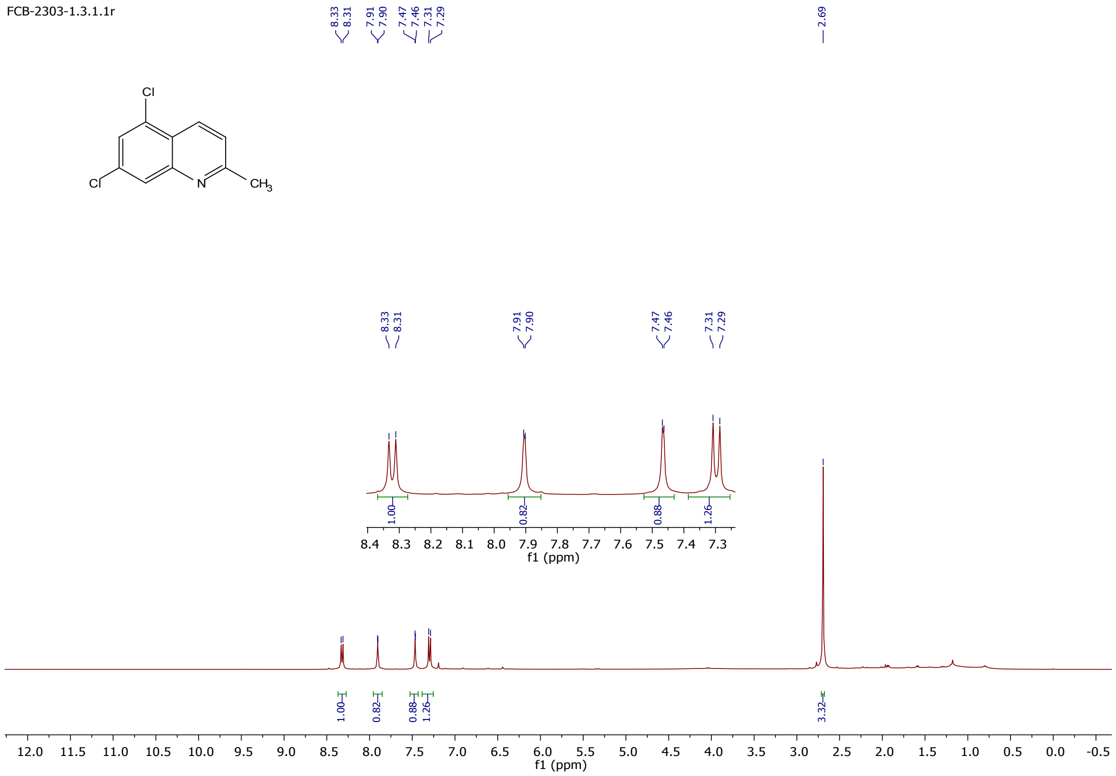

Figure S25: ^{1}H NMR (400MHz, $CDCl_3$) of intermediate 2.

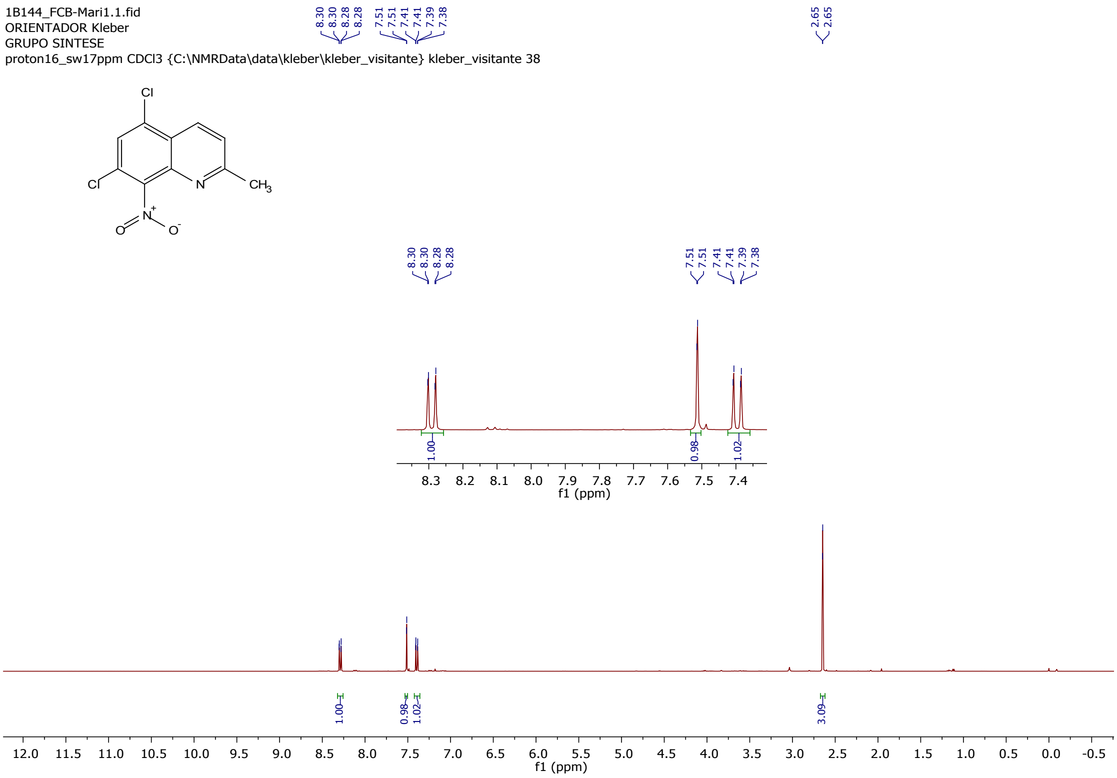

Figure S26: ^{1}H NMR (400MHz, $CDCl_3$) of intermediate 3.

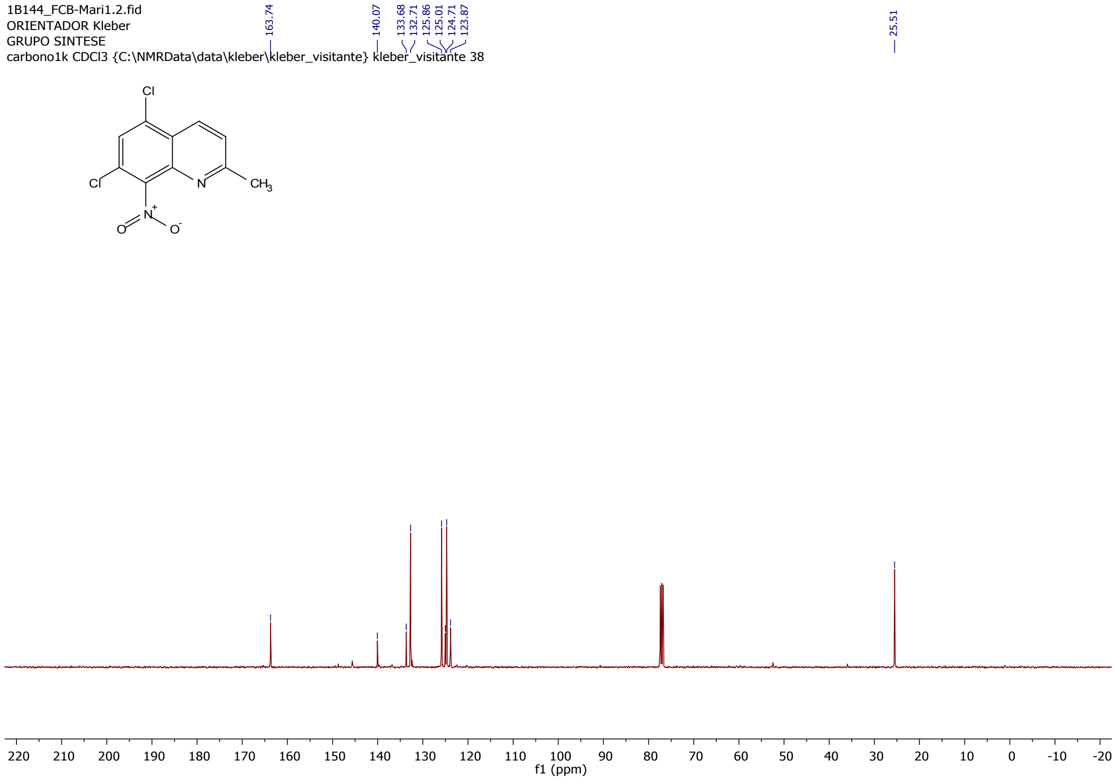

Figure S27: ^{13}C NMR (400MHz, $CDCl_3$) of intermediate 3.

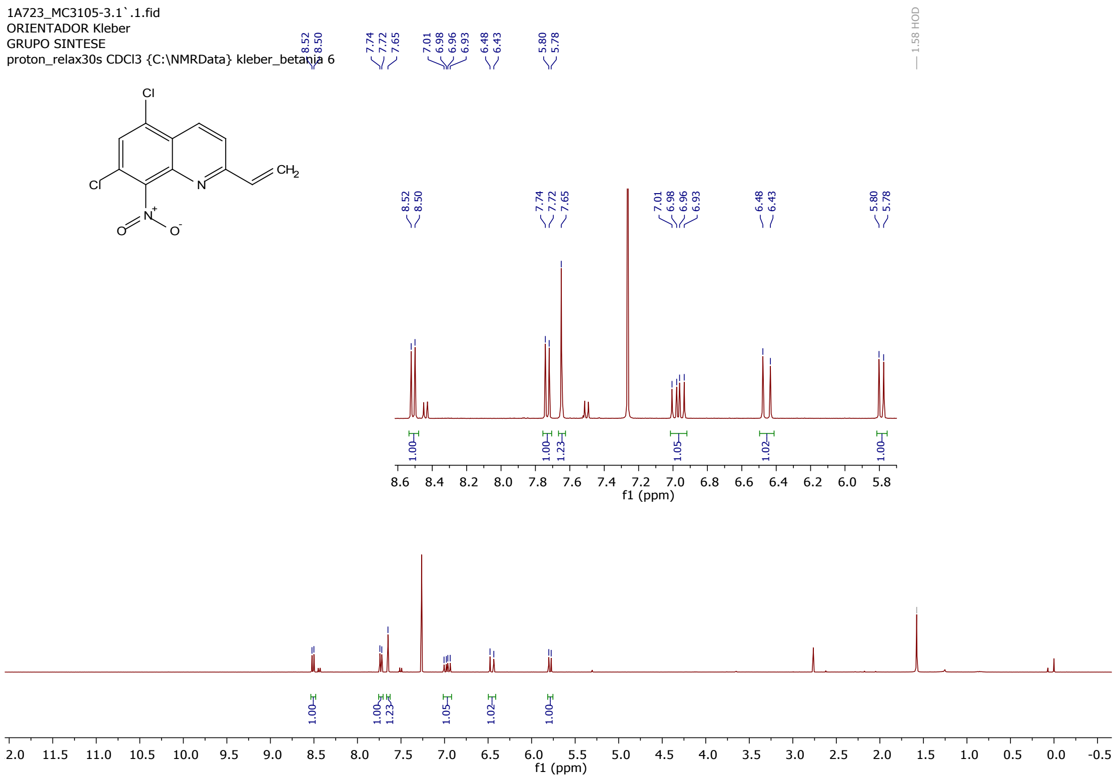

Figure S28: ^{1}H NMR (400MHz, $CDCl_3$) of intermediate 4. The signals that were not picked belong to the starting material leftover.

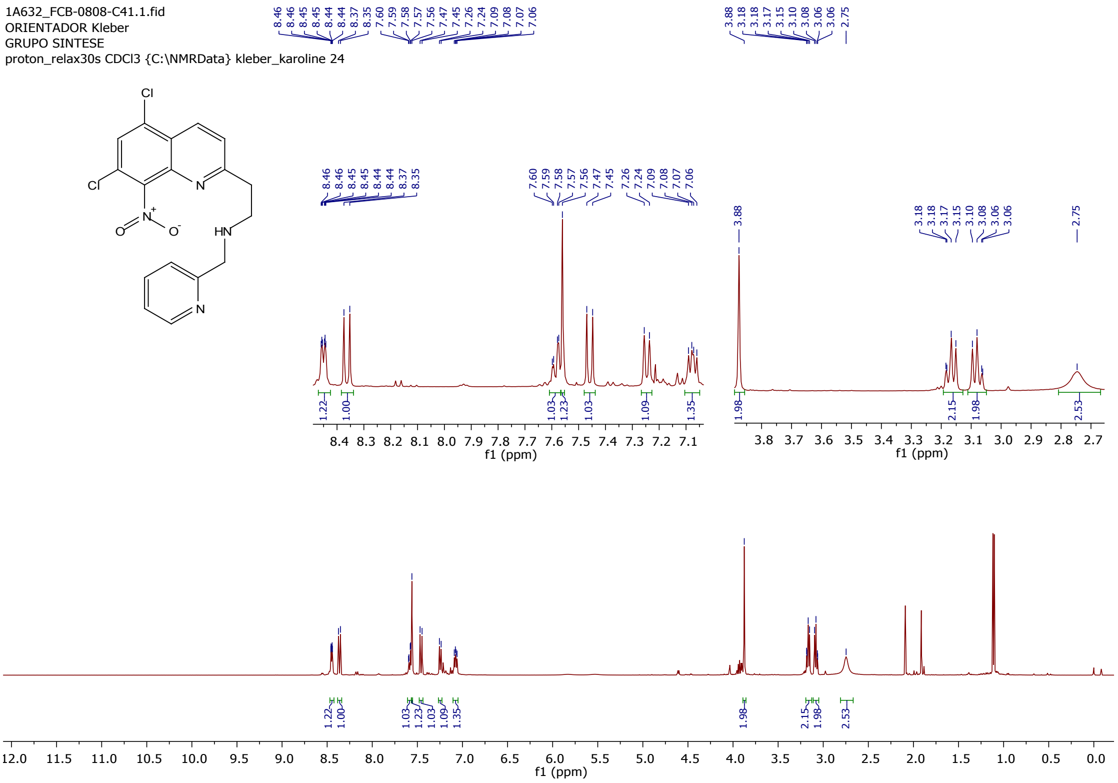

Figure S29: ^{1}H NMR (400MHz, $CDCl_3$) of intermediate 5. Signals at 3.92 and 1.13 ppm are attributed to isopropanol used in the column.

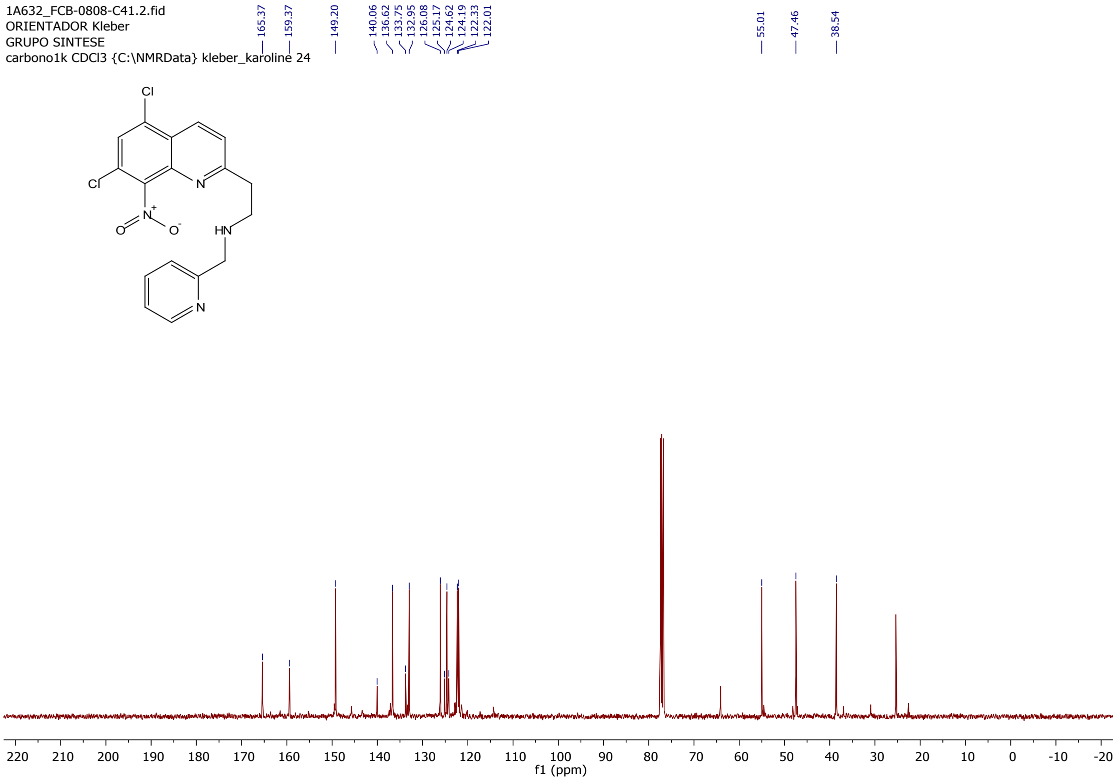

Figure S30: ^{13}C NMR (400MHz, $CDCl_3$) of intermediate 5. Signals at 64.13 and 25.20 ppm are attributed to isopropanol used in the column

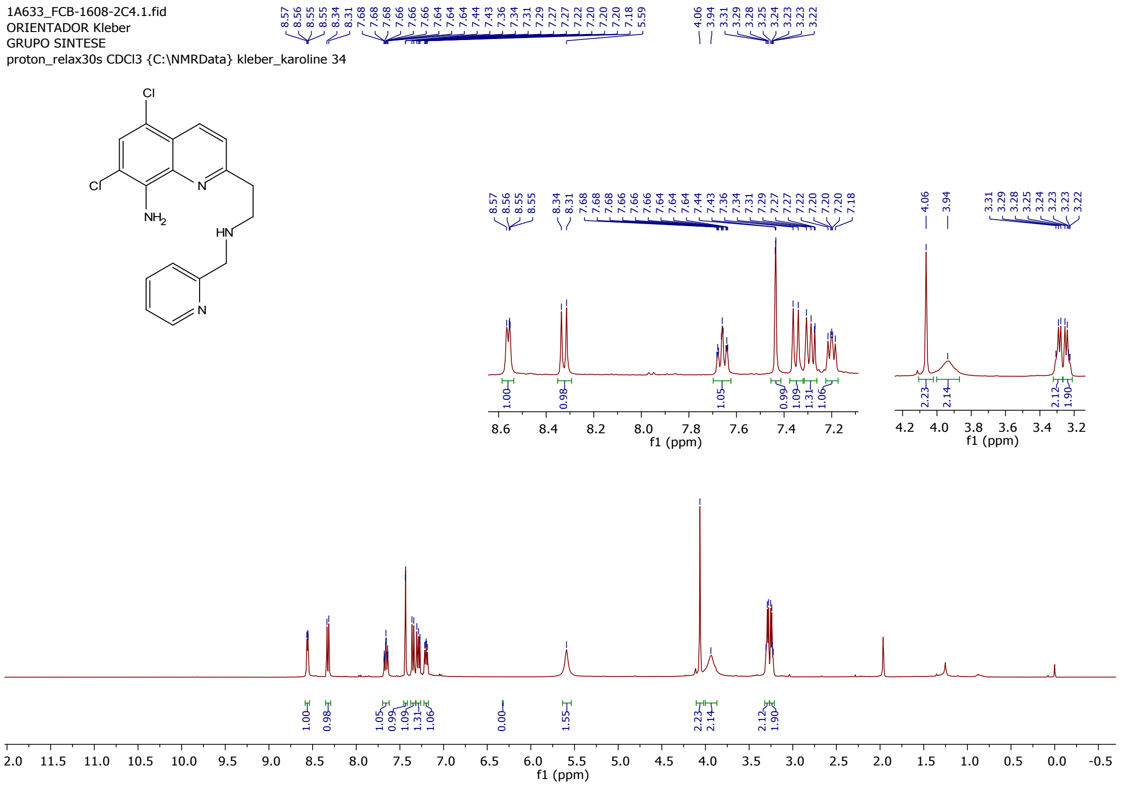

Figure S31: ^{1}H NMR (400MHz, $CDCl_3$) of L11.

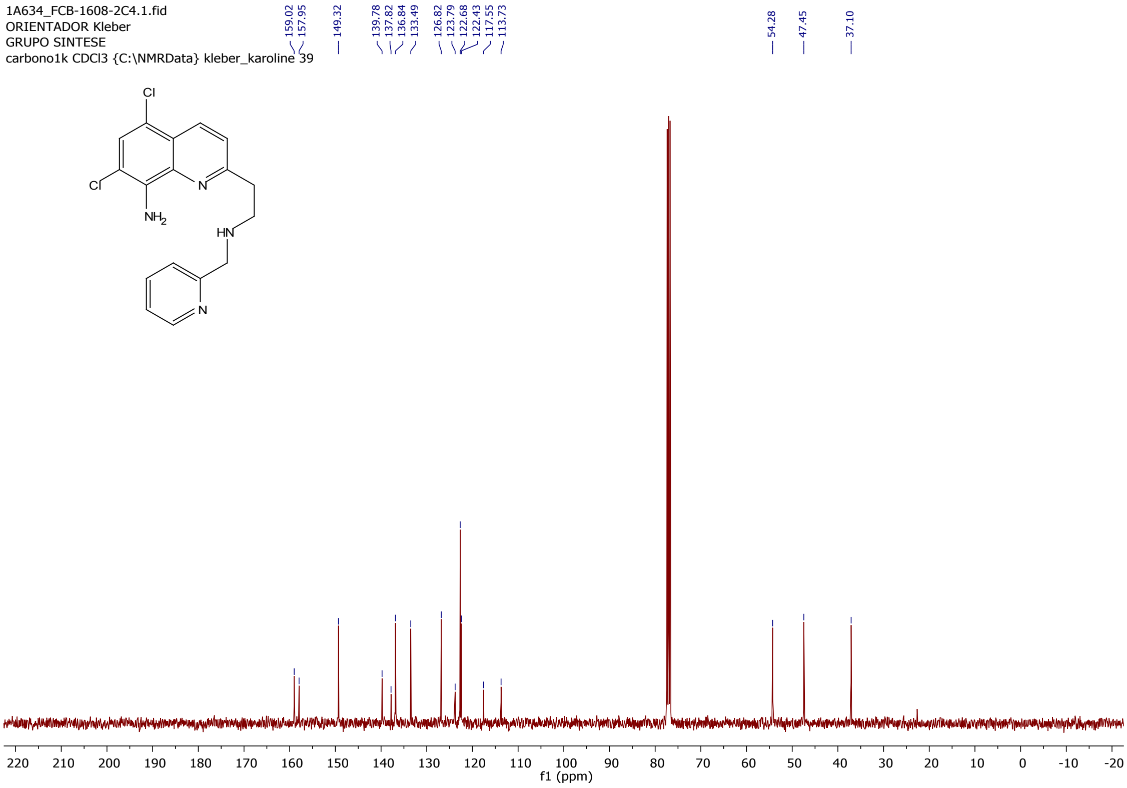

Figure S32: ^{13}C NMR (400MHz, $CDCl_3$) of L11.

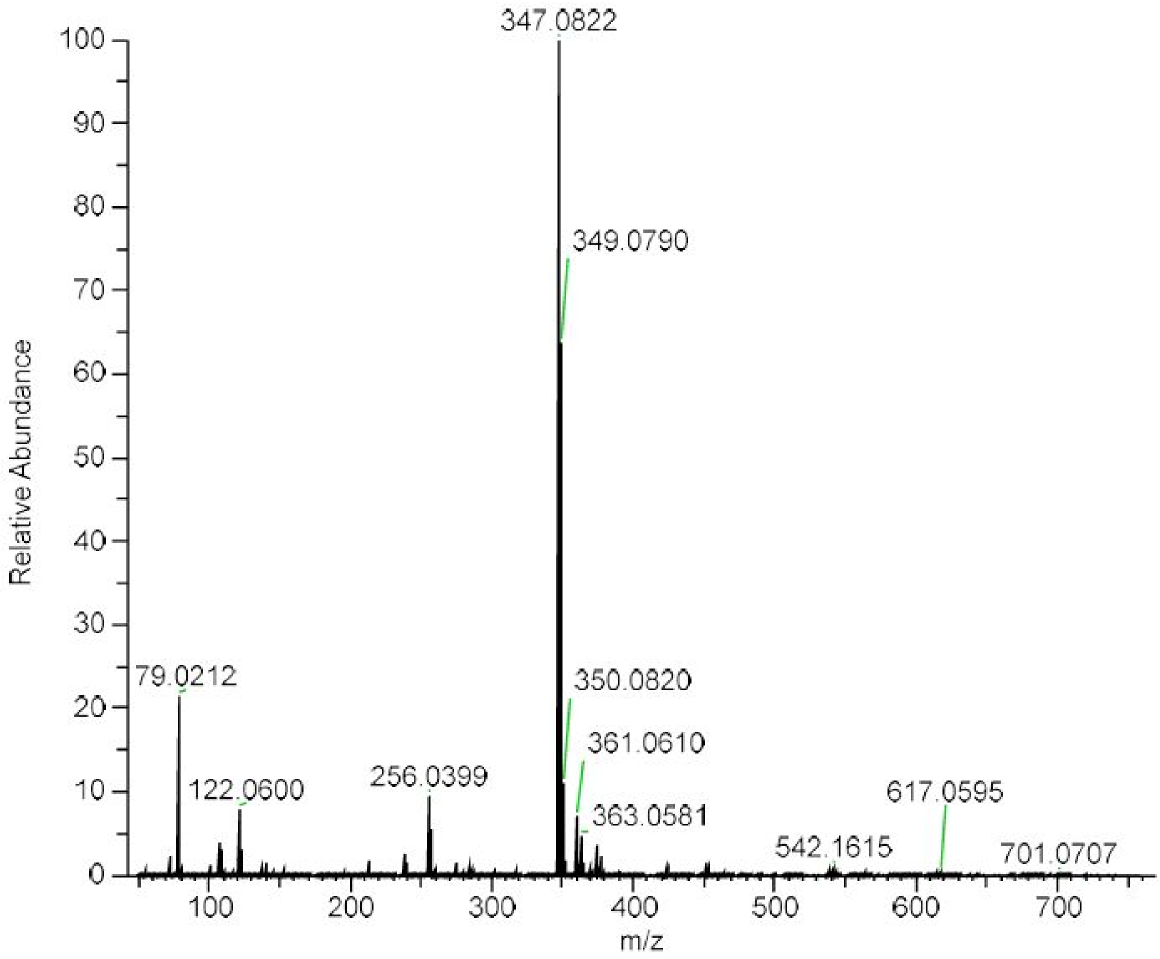

Figure S33: HR-ESI-MS Spectrum of L11

In silico assay

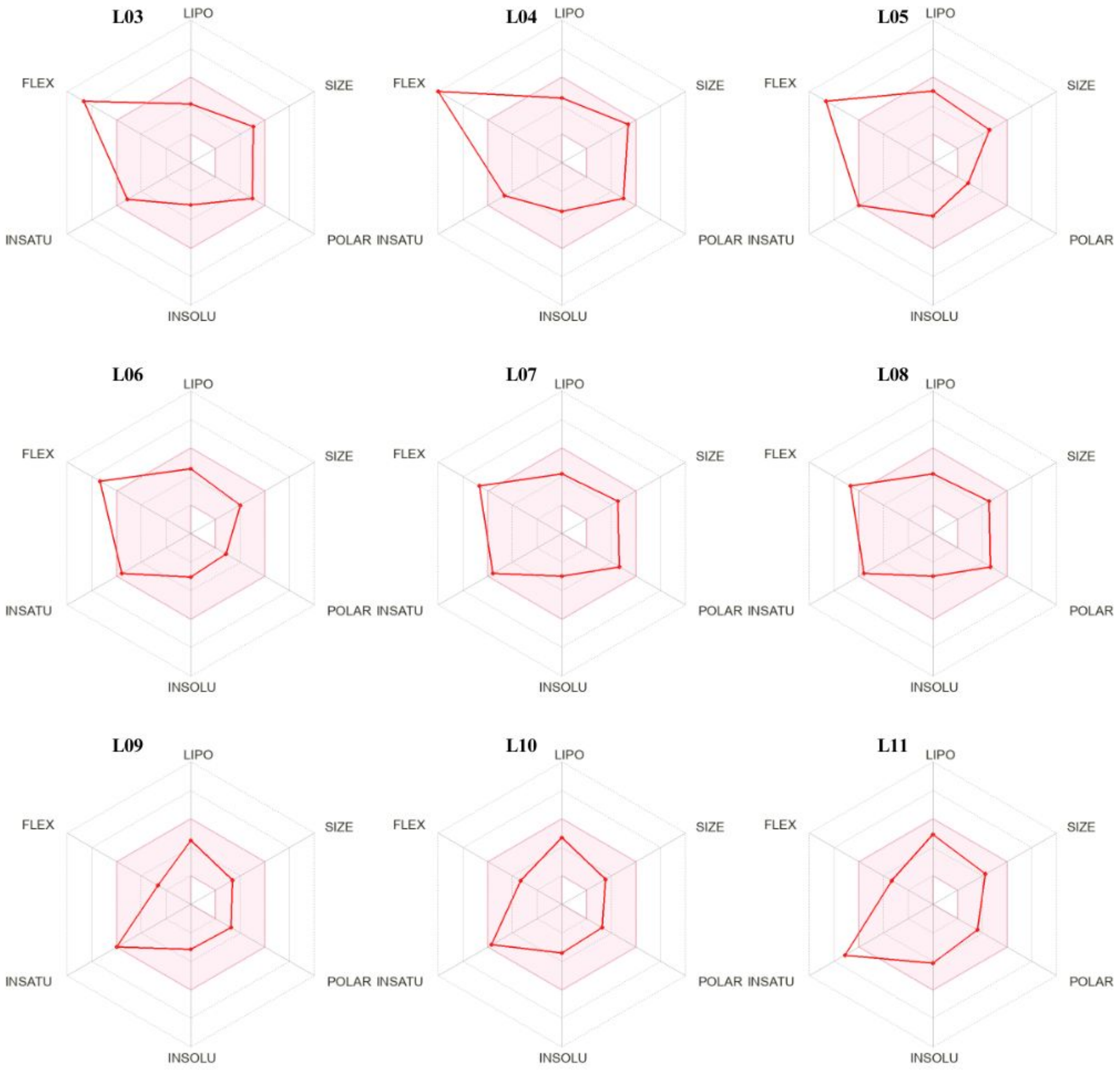

Figure S34: Panel containing the bioavailability radars of compounds L03–L11, obtained in *in silico* tests.

UV-Vis Spectra

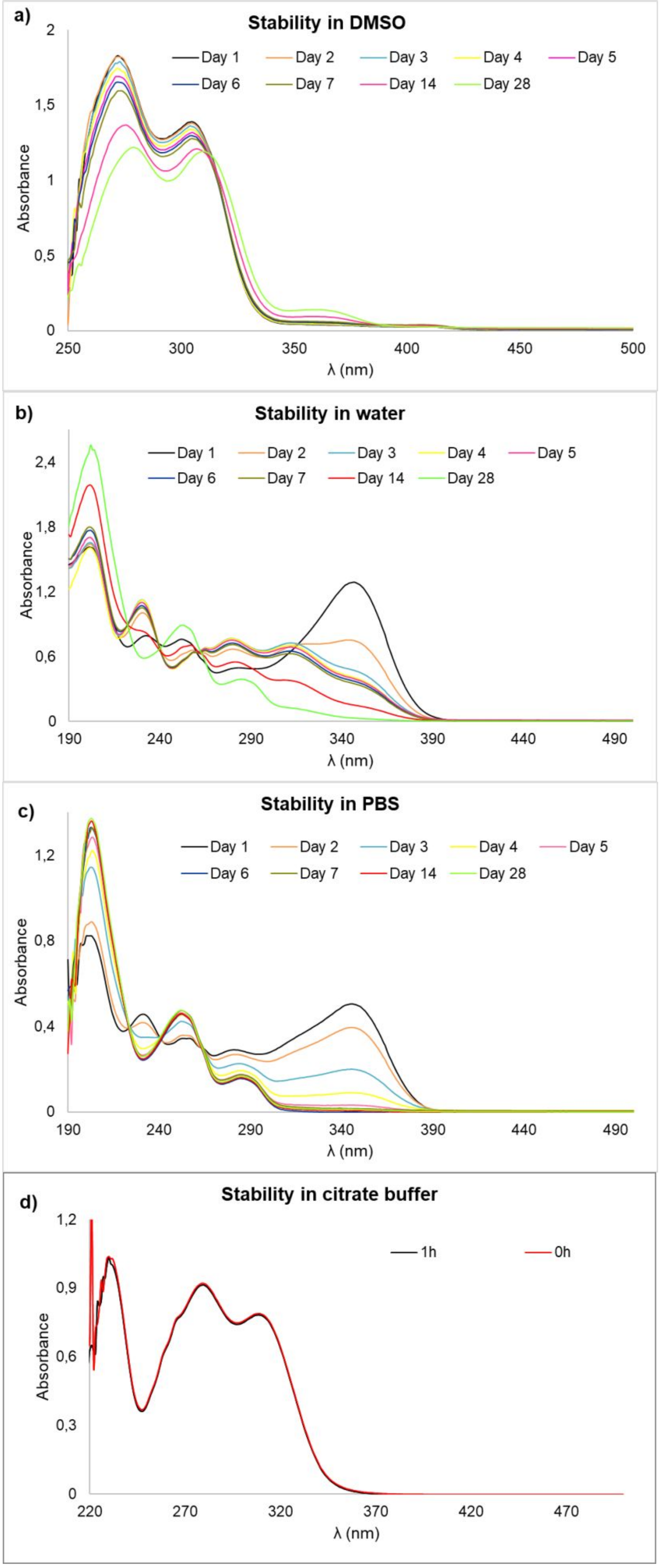

Figure S35: Stability study of the L10 ligand, by UV-Vis spectroscopy, in (**a**) DMSO, (**b**) water, (**c**) PBS and (**d**) citrate buffer

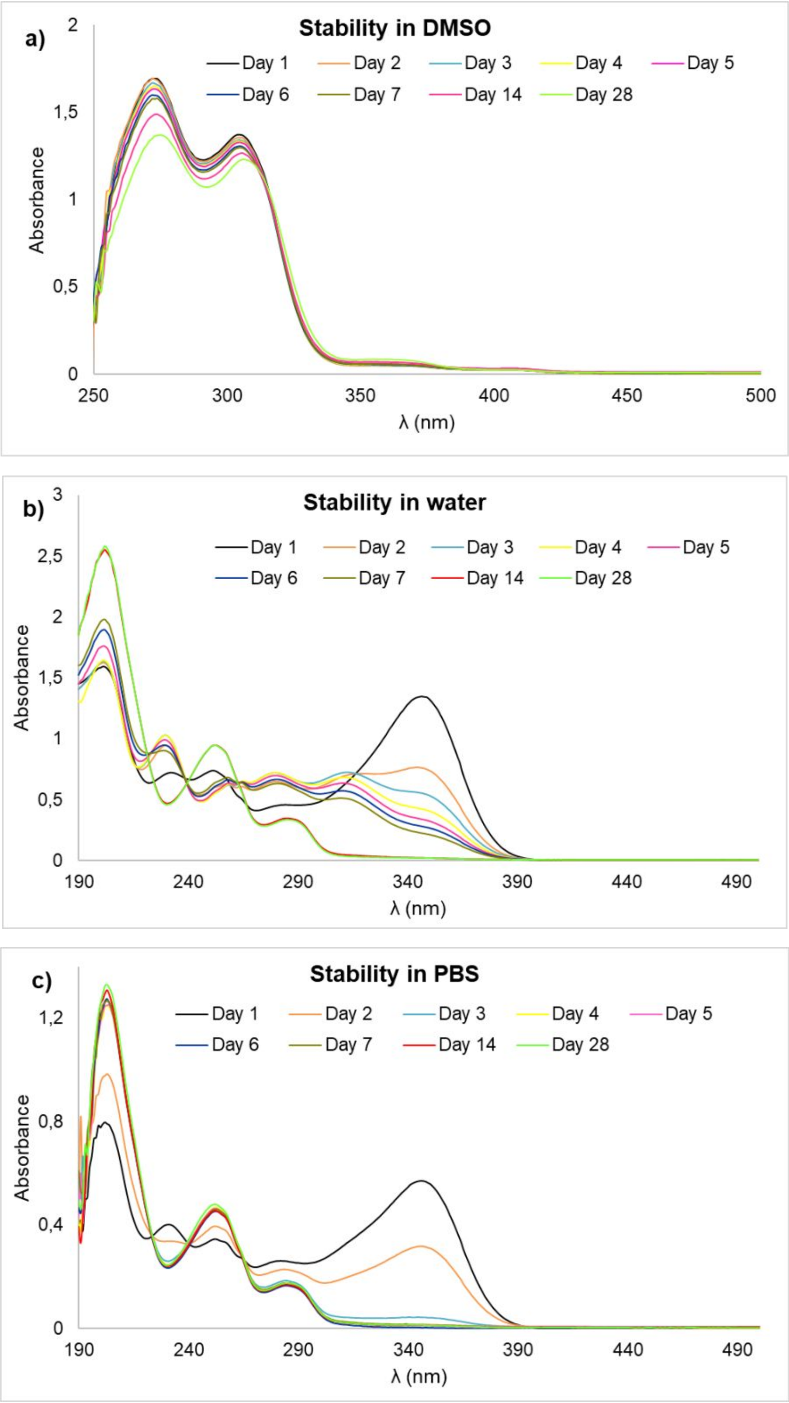

Figure S36: Stability study of the L09 ligand, by UV-Vis spectroscopy, in (**a**) DMSO, (**b**) water and (**c**) PBS

Immunofluorescence analysis

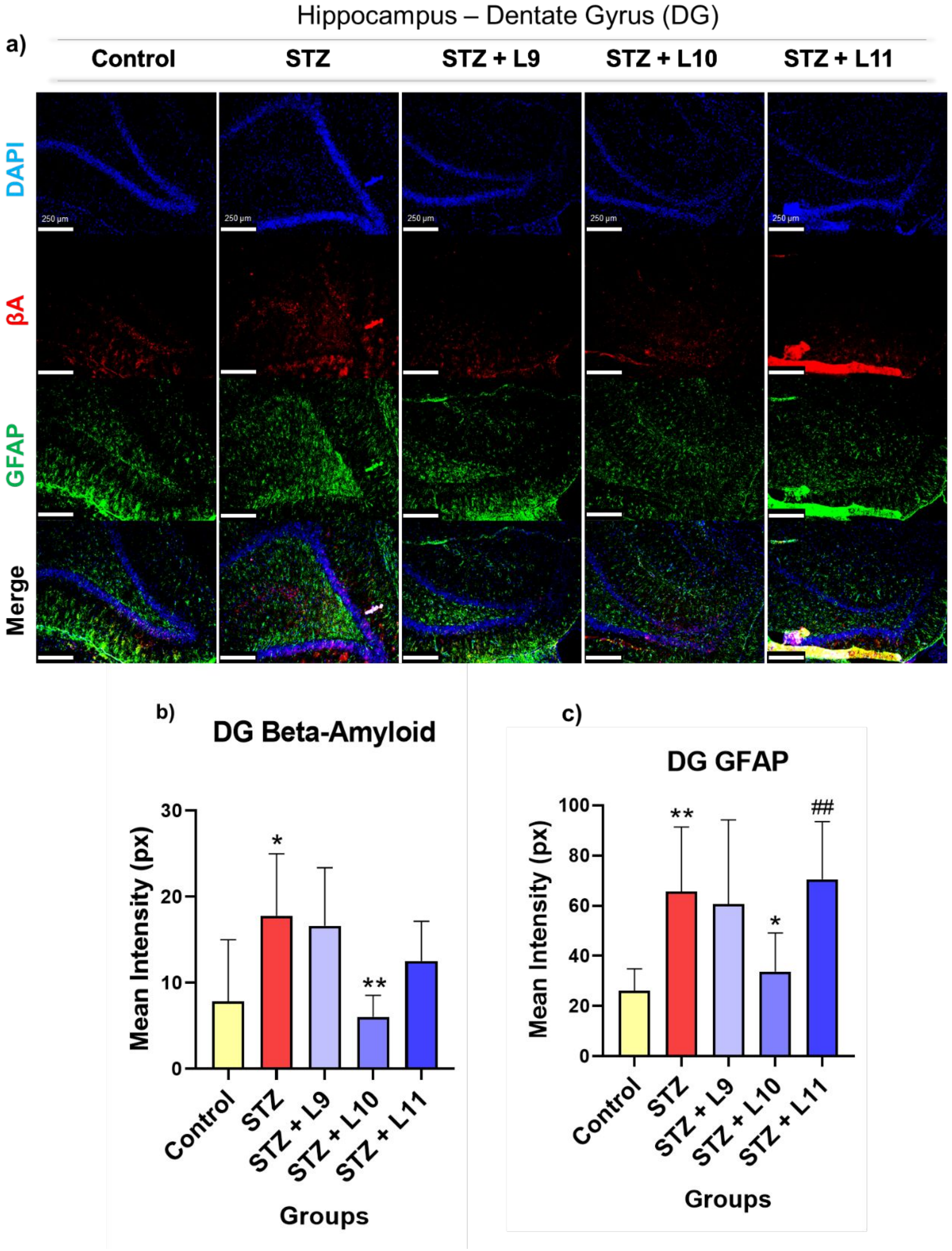

Figure S37: Improvement in the control of neuroinflammation and reduced Aβ plaques caused by treatment with the compounds. (**a**) Representative immunofluorescence images of the Control, STZ, STZ+L9, STZ+L10, and STZ+L11 groups in the DG region of the hippocampus for Aβ (red), GFAP (green), and DAPI (blue). (**b**), (**c**) Signal intensity was quantified using graphs, where the mean fluorescence intensity is represented by bars, with their standard error bars shown.

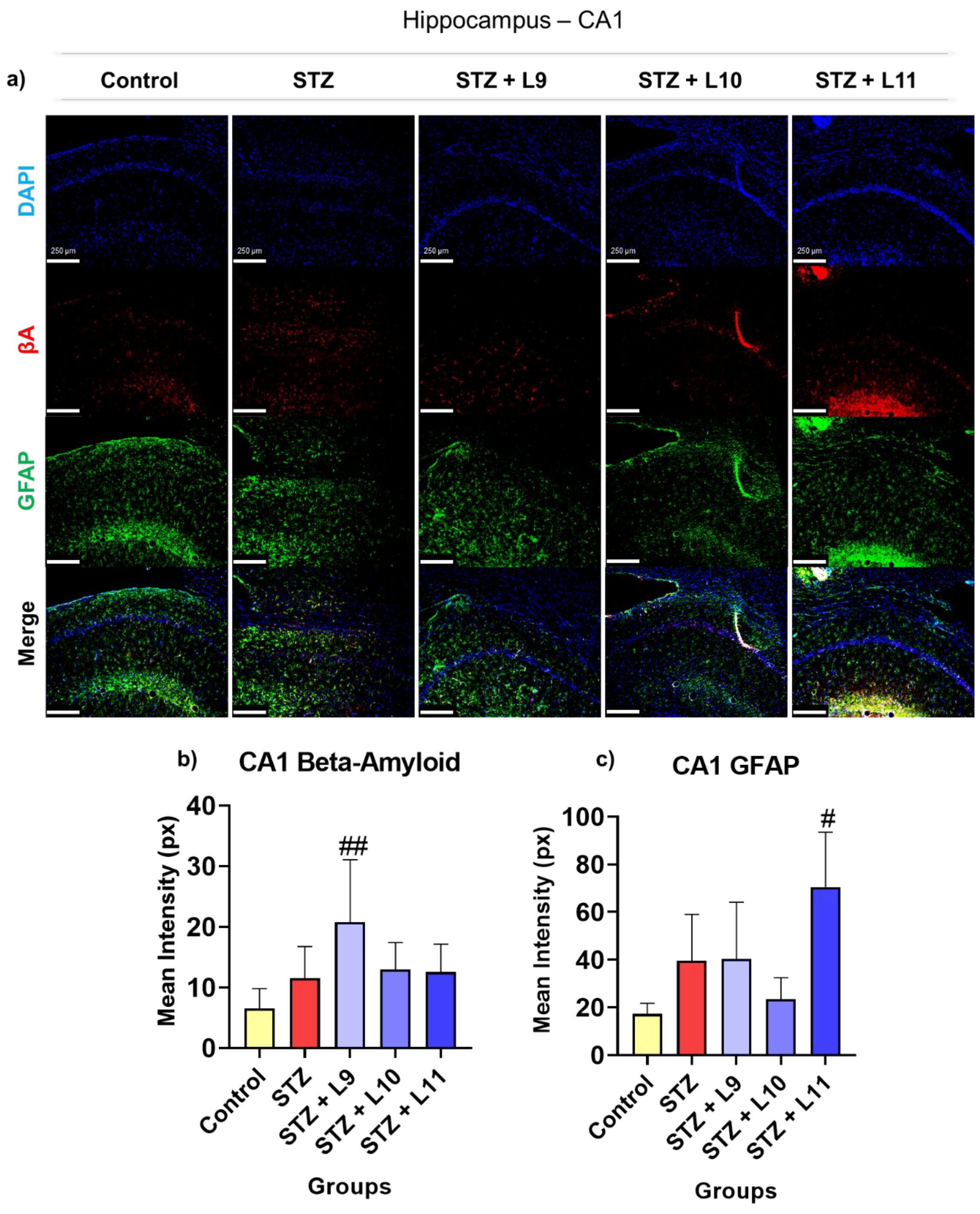

Figure S38: Improvement in the control of neuroinflammation and reduced Aβ plaques caused by treatment with the compounds. (**a**) Representative immunofluorescence images of the Control, STZ, STZ+L9, STZ+L10, and STZ+L11 groups in the CA1 region of the hippocampus for Aβ (red), GFAP (green), and DAPI (blue). (**b**), (**c**) Signal intensity was quantified using graphs, where the mean fluorescence intensity is represented by bars, with their standard error bars shown.

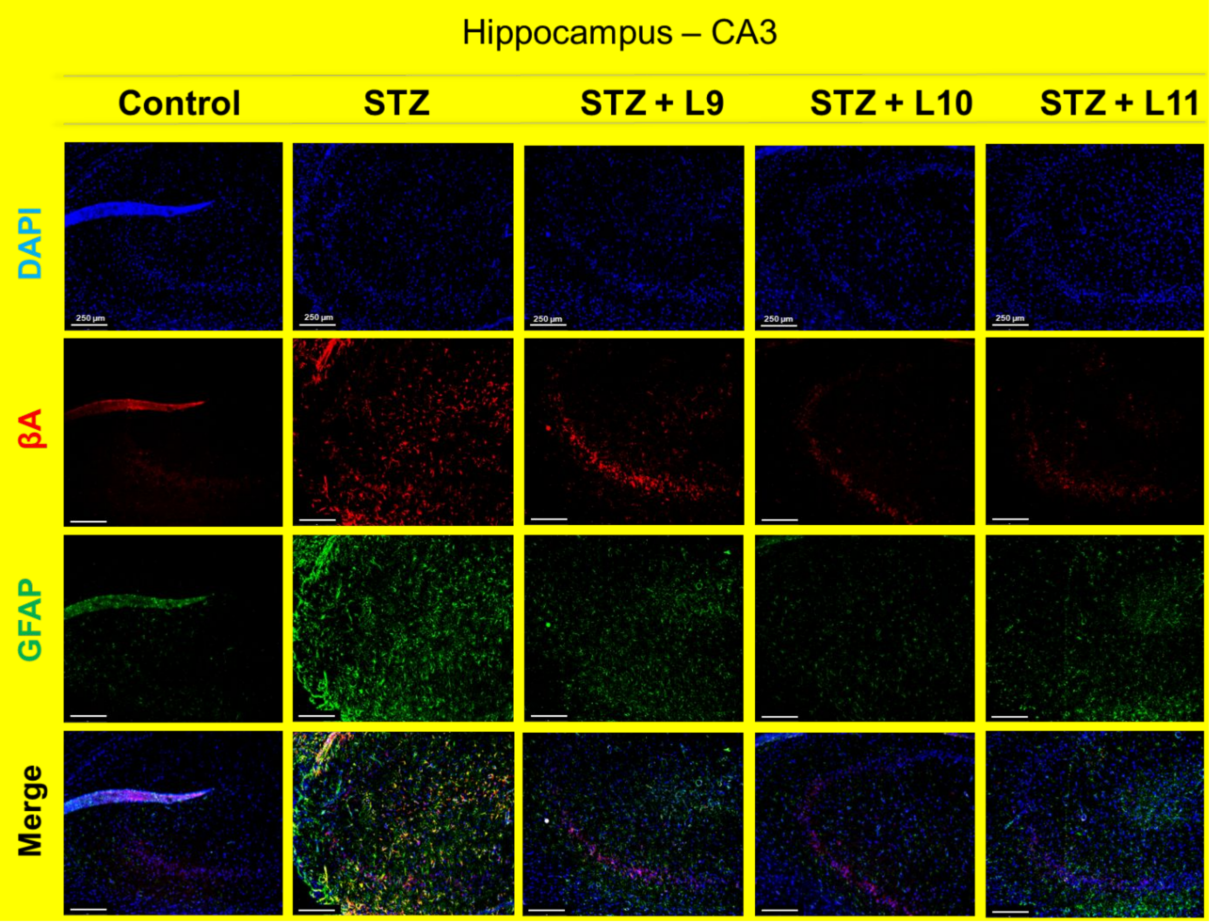

Figure S39: Improvement in the control of neuroinflammation and reduced Aβ plaques caused by treatment with the compounds. (**a**) Representative immunofluorescence images of the Control, STZ, STZ+L9, STZ+L10, and STZ+L11 groups in the CA3 region of the hippocampus for Aβ (red), GFAP (green), and DAPI (blue).

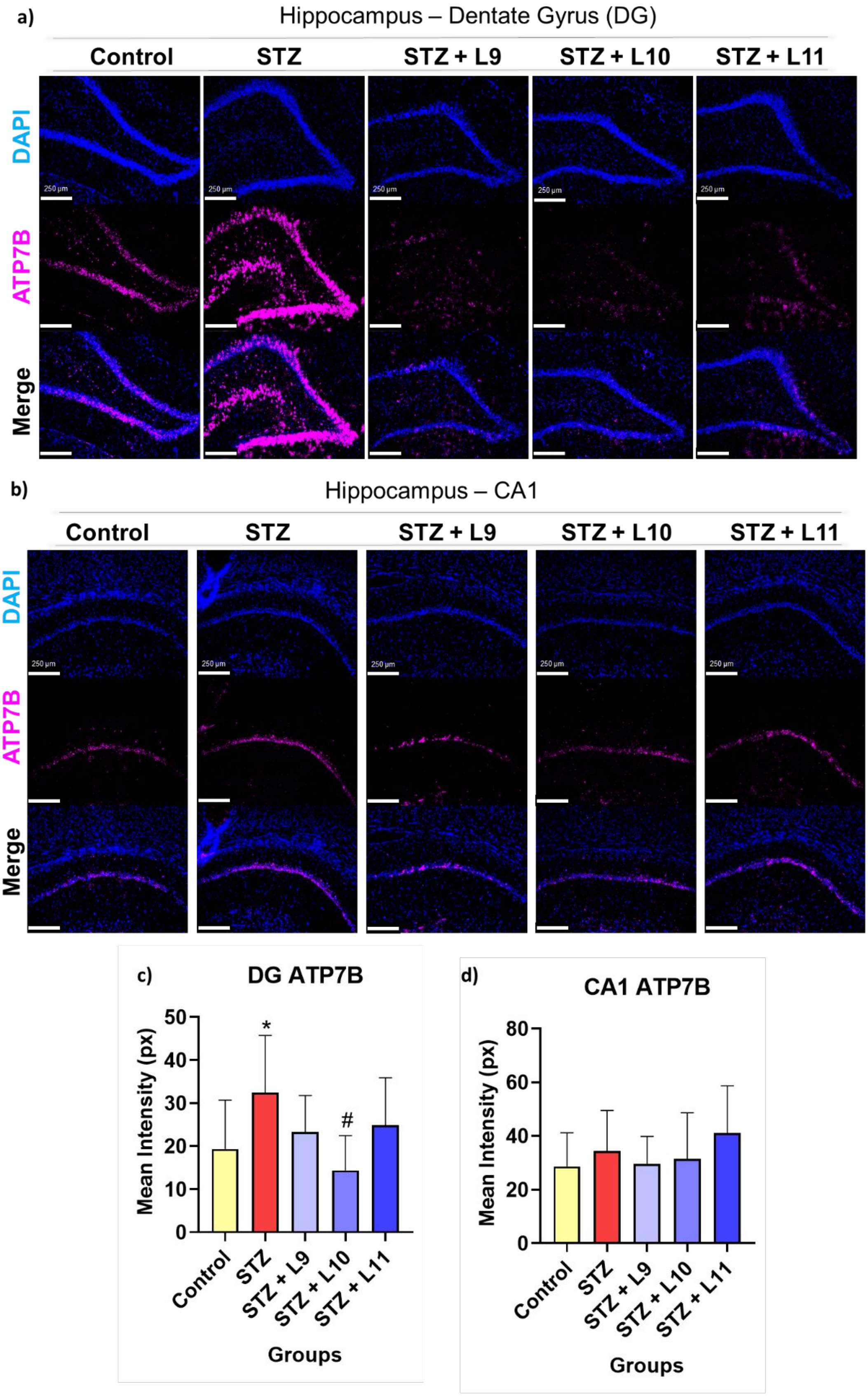

Figure S40: Regulation of ATP7B expression levels resulting from treatment with the new ligands. Representative immunofluorescence images of the Control, STZ, STZ+L9, STZ+L10, and STZ+L11 groups in the (**a**) DG and (**b**) CA1 regions of the hippocampus for ATP7B (pink) and DAPI (blue). The signal intensity in the (**c**) DG and (**d**) CA1 regions was quantified using graphs, where the mean fluorescence intensity is represented by bars, with their standard error bars shown.

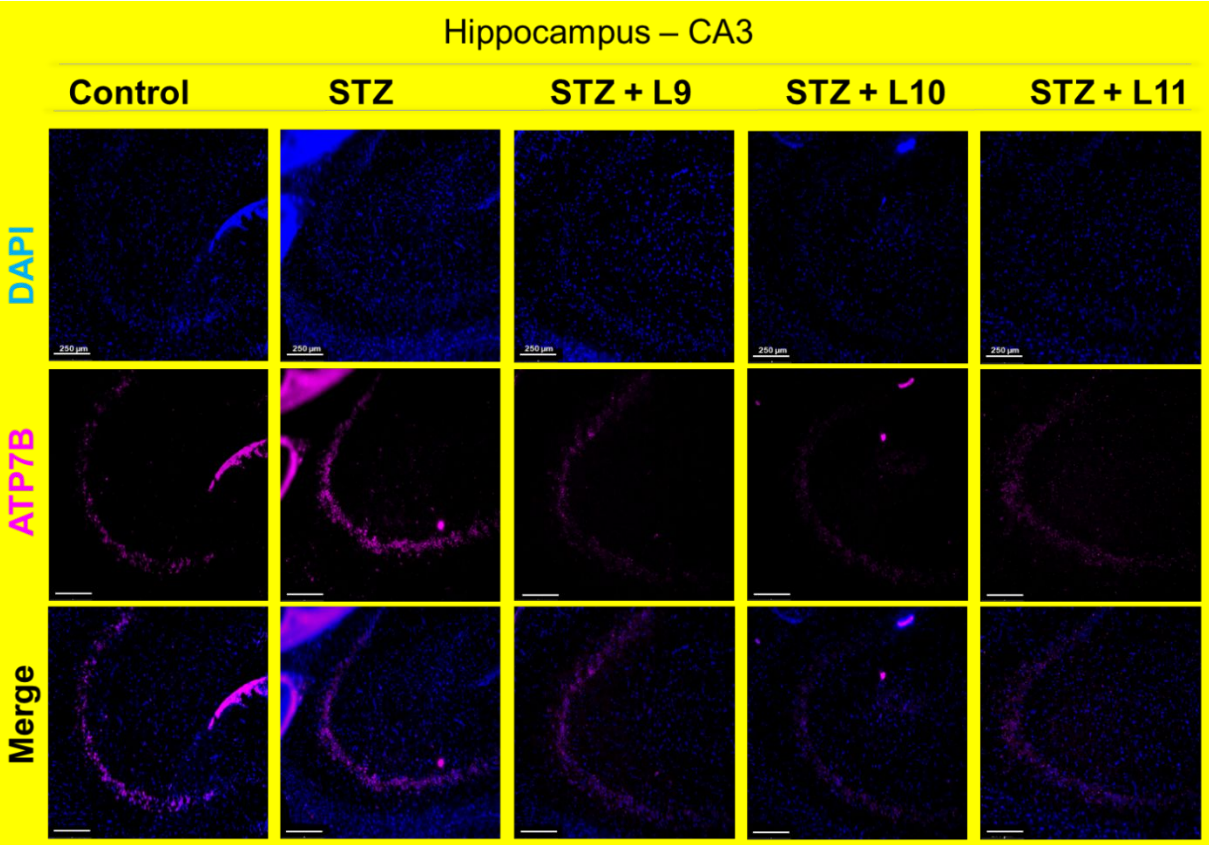

Figure S41: Regulation of ATP7B expression levels resulting from treatment with the new ligands. Representative immunofluorescence images of the Control, STZ, STZ+L9, STZ+L10, and STZ+L11 groups in the CA3 region of the hippocampus for ATP7B (pink) and DAPI (blue).

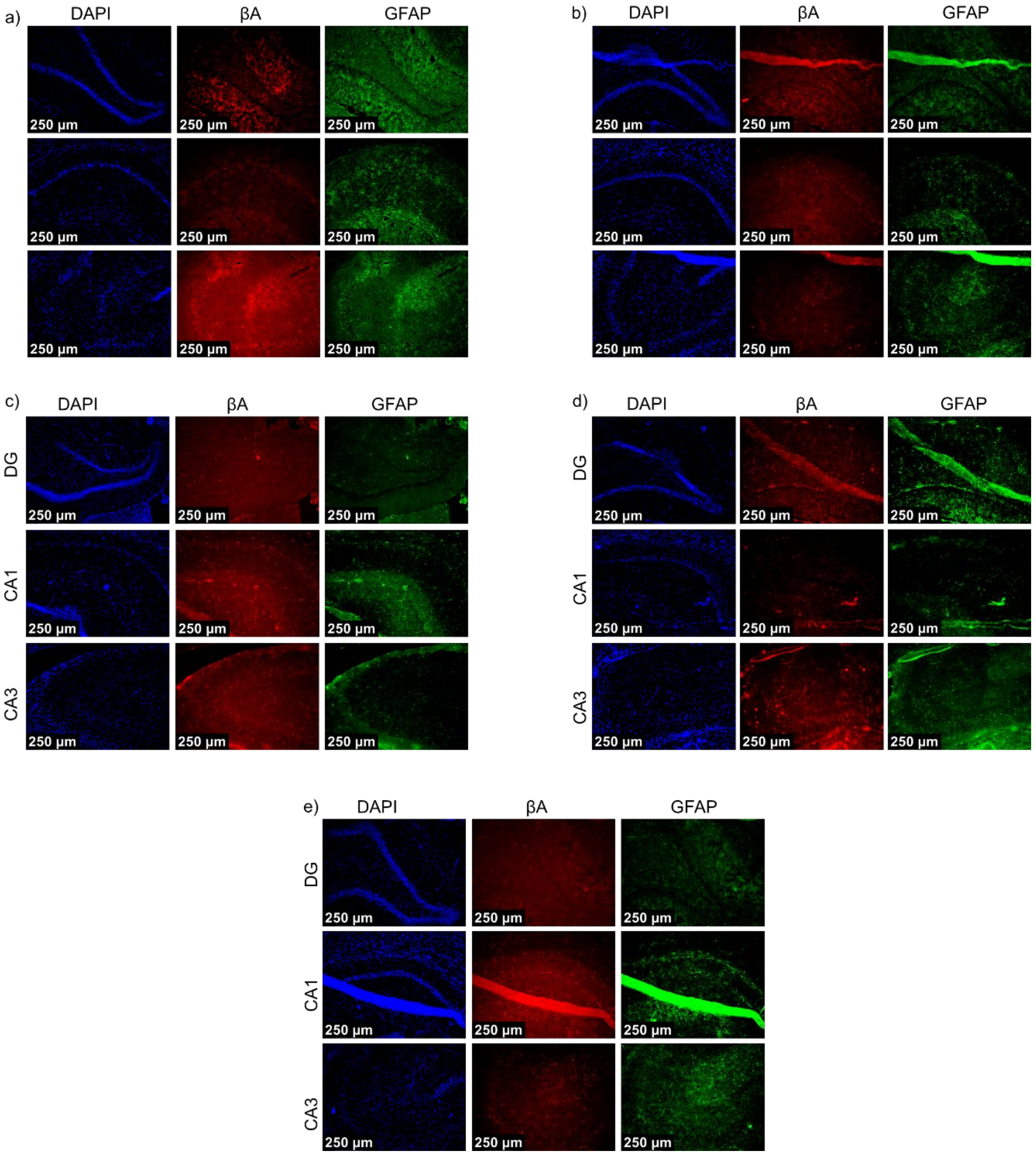

Figure S42: Negative control for GFAP and Aβ analysis by immunofluorescence, being (**a**) control, (**b**) STZ, (**c**) STZ + L9, (**d**) STZ + L10 and (**e**) STZ + L11

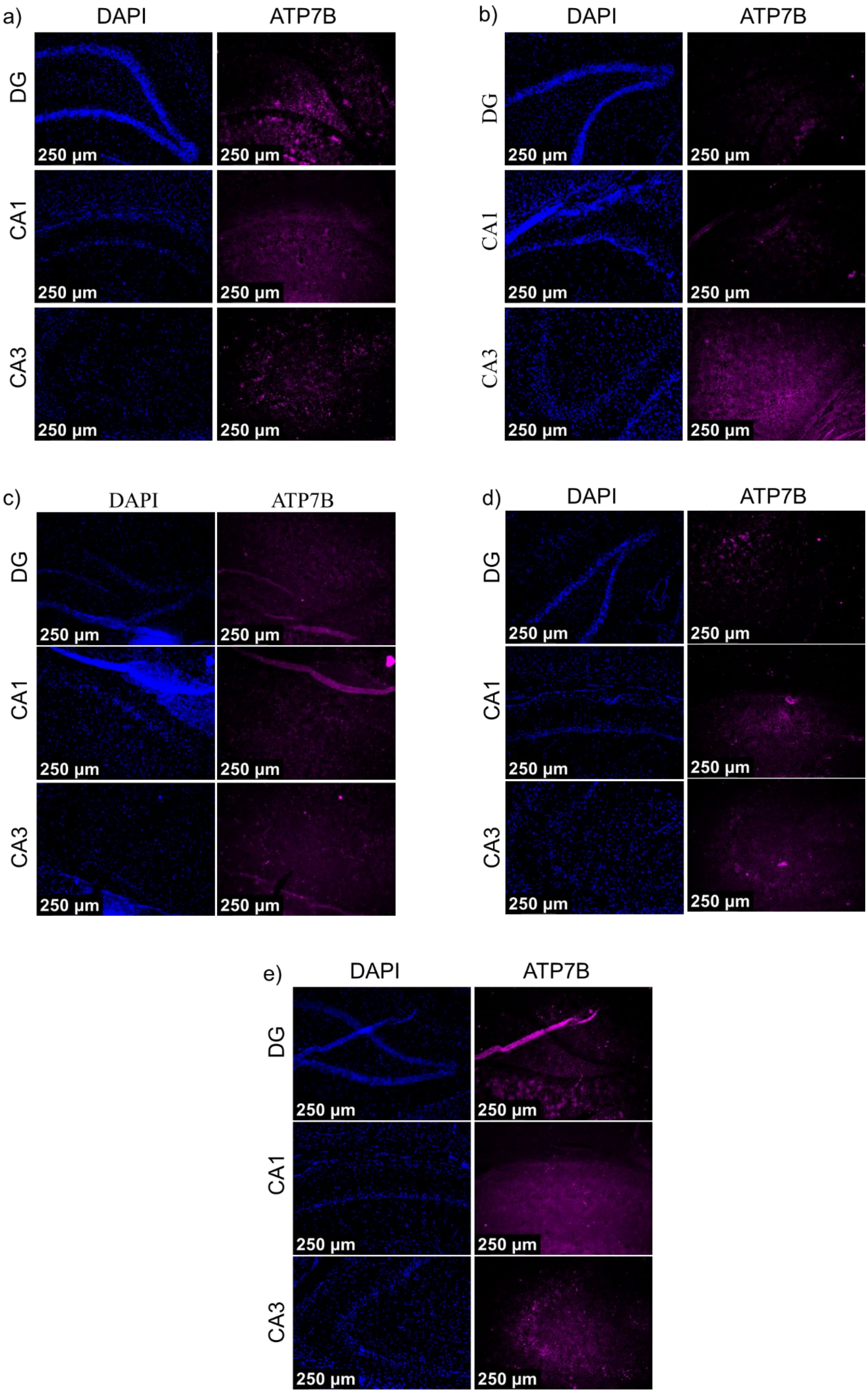

Figure S43: Negative control for ATP7B analysis by immunofluorescence, being (**a**) control, (**b**) STZ, (**c**) STZ + L9, (**d**) STZ + L10 and (**e**) STZ + L11
